# Phytochemical Compound Screening to Identify Novel Small Molecules against Dengue Virus: A Docking and Dynamics Study

**DOI:** 10.3390/molecules27030653

**Published:** 2022-01-20

**Authors:** Mst. Sharmin Sultana Shimu, Shafi Mahmud, Trina Ekwati Tallei, Saad Ahmed Sami, Ahmad Akroman Adam, Uzzal Kumar Acharjee, Gobindo Kumar Paul, Talha Bin Emran, Shahriar Zaman, Md. Salah Uddin, Md. Abu Saleh, Sultan Alshehri, Mohammed M Ghoneim, Maha Alruwali, Ahmad J. Obaidullah, Nabilah Rahman Jui, Junghwan Kim, Bonglee Kim

**Affiliations:** 1 Department of Genetic Engineering and Biotechnology, University of Rajshahi, Rajshahi 6205, Bangladesh; sharminshimu120@gmail.com; 2 Microbiology Laboratory, Department of Genetic Engineering and Biotechnology, University of Rajshahi, Rajshahi 6205, Bangladesh; shafimahmudfz@gmail.com (S.M.); gobindokumar38@gmail.com (G.K.P.); szaman@ru.ac.bd (S.Z.); salim.geb@ru.ac.bd (M.S.U.); 3 Department of Biology, Faculty of Mathematics and Natural Science, Sam Ratulangi University, Manado 95115, Indonesia; trina_tallei@unsrat.ac.id; 4 Department of Pharmacy, University of Chittagong, Chittagong 4331, Bangladesh; s.a.sami18pharm@gmail.com; 5 Dentistry Study Program, Faculty of Medicine, Sam Ratulangi University, Manado 95115, Indonesia; ahmad_adam@ymail.com; 6 Department of Pharmacy, BGC Trust University Bangladesh, Chittagong 4381, Bangladesh; talhabmb@bgctub.ac.bd; 7 Department of Pharamaceutics, College of Pharmacy, King Saud University, Riyadh 11451, Saudi Arabia; salshehri1@ksu.edu.sa; 8 Department of Pharmacy Practice, College of Pharamcy, AlMaarefa University, Ad Diriyah 13713, Saudi Arabia; mghoneim@mcst.ed (M.M.G.); mrowaili@mcst.edu.sa (M.A.); 9 Drug Exploration and Development Chair (DEDC), Department of Pharmaceutical Chemistry, College of Pharmacy, King Saud University, Riyadh 11451, Saudi Arabia; aobaidullah@ksu.ed; 10 Department of Pharmaceutical Chemistry, College of Pharmacy, King Saud University, Riyadh 11451, Saudi Arabia; 11 Department of Biochemistry and Biotechnology, University of Science and Technology, Chittagong 4202, Bangladesh; Nrjjui708@gmail.com; 12 Department of Internal Medicine, College of Korean Medicine, Kyung Hee University, Seoul 02447, Korea; akom21@khu.ac.kr; 13 Department of Pathology, College of Korean Medicine, Kyung Hee University, Hoegidong Dongdaemungu, Seoul 05253, Korea

**Keywords:** phytochemicals, Dengue virus, NS2B/NS3 protein, molecular docking, molecular dynamics

## Abstract

The spread of the Dengue virus over the world, as well as multiple outbreaks of different serotypes, has resulted in a large number of deaths and a medical emergency, as no viable medications to treat Dengue virus patients have yet been found. In this paper, we provide an in silico virtual screening and molecular dynamics-based analysis to uncover efficient Dengue infection inhibitors. Based on a Google search and literature mining, a large phytochemical library was generated and employed as ligand molecules. In this investigation, the protein target NS2B/NS3 from Dengue was employed, and around 27 compounds were evaluated in a docking study. Phellodendroside (−63 kcal/mole), quercimeritrin (−59.5 kcal/mole), and quercetin-7-*O*-rutinoside (−54.1 kcal/mole) were chosen based on their binding free energy in MM-GBSA. The tested compounds generated numerous interactions at Lys74, Asn152, and Gln167 residues in the active regions of NS2B/NS3, which is needed for the protein’s inhibition. As a result, the stable mode of docked complexes is defined by various descriptors from molecular dynamics simulations, such as RMSD, SASA, Rg, RMSF, and hydrogen bond. The pharmacological properties of the compounds were also investigated, and no toxicity was found in computational ADMET properties calculations. As a result, this computational analysis may aid fellow researchers in developing innovative Dengue virus inhibitors.

## 1. Introduction

The Dengue virus is spread by the mosquito *Aedes aegypti*, which is mostly found in tropical and subtropical areas [1]. Currently, 2.5 billion people around the world are at danger of contracting Dengue fever, with hundreds of millions of people suffering from the disease [2]. Climate change, population increase, and deforestation all contribute to the spread of Dengue fever, resulting in recurring outbreaks and more intense transmission of diverse serotypes [3]. The Dengue virus has four different serotypes, and infection with one of them does not confer protection against the other serotypes [4]. As a result, Dengue infections caused by two different serotypes carry the risk of Dengue hemorrhagic fever [5]. Despite the fact that many studies are still ongoing and that a large number of people are affected by Dengue fever on a daily basis, no effective inhibitors have been identified. As a result, a novel technique for the identification of a powerful Dengue inhibitor is required [6,7].

As a result, the Dengue virus is classified as an RNA virus with a single-stranded positive-sense RNA genome of 10.7 kb [8,9,10]. The Dengue virus RNA genome is transcribed as a single polyprotein, and subsequently co- and post-translationally cleaved into three structural proteins (Capsid (C), pre-membrane (prM), envelope (E) and seven non-structural proteins (NS1, NS2A, NS2B, NS3, NS4A, NS4B, and NS5) [11,12,13]. These structural proteins, on the other hand, mediate the proteolytic processing that is mediated by the endoplasmic reticulum-based host cell signal peptidase [14]. Cleavage occurs in the NS2A/NS2B, NS2B/NS3, NS3/NS4A, and NS4B/NS5 sites by the NS2B/NS3 protease. As a result, this viral protease from Dengue also cleaves the internal side of C, NS2A, NS3, NS4A, and C/prM. As a result, the NS2B/NS3 function as a key viral protease, which is significant for the development of antiviral drugs against the Dengue virus that can interfere with the virus’s functions [15,16].

Multiple efforts are currently underway to find efficient Dengue virus inhibitors and vaccines by targeting various structural and non-structural proteins. As a result, Dengvaxia, a Dengue vaccine, has been studied in two independent phase 3 clinical trials in Latin America and Asia, with efficacy ranging from 31.3 percent to 79 percent, demonstrating diversity in vaccine efficacy across different geographies and serotypes [17,18]. Despite various efforts by experts, there are currently no antiviral medications available on the market for the Dengue virus. Furthermore, Zingiberaceae chalcone compounds have been shown to inhibit the DENV2 protease. Various isolated substances, such as panduratin A and 4-hydroxypanduratin, also have inhibitory activity [19]. Quercetin and agathisflavone [20], dehydronaphthalene [21], benzimidazole [22], thiadiazoloacrylamide [23], and drug designing process [24,25] are some of the powerful compounds that target the NSB2/NS3 from phytochemicals.

Curdlan sulfate [26], rolitetracycline [26], fucoidan [27], balapiravir [28], suramin [29], carnosine [30], policresulen [31], dasatinib [32], duramycin [33], luteolin [34,35], and imatinib [36] are examples of DAA or direct acting antivirals that efficiently interact by targeting NS and structural proteins. DAA, on the other hand, has the ability to target viral proteins and usually has a lower level of toxicity, but it comes with the acknowledged drawback of a higher likelihood of resistance development [37]. Furthermore, numerous DAA candidates for Dengue failed in mice experiments despite showing promise in in vitro assays, and only balapiravir has been studied in human trials among many DAA candidates [38]. Furthermore, due to the lower chance of acquiring resistance, host directed antivirals, or HDAs, have a lot of potential in a broader range, but only a few candidates have made it to the clinical trial stage [39]. Cleavages of florescence energy transfer (FRET), HPLC-based detection, isothermal titration calorimetry, and surface plasmon resonance have also been used to identify various protease inhibitors [37,40,41,42].

We conducted a virtual screening working flow of phytochemicals against the viral protein target of NS2B/NS3 in this investigation. The binding affinity and MM-GBSA techniques aid in the screening of phytochemical libraries and the selection of molecules with the highest binding energy. A molecular dynamics simulation was also carried out to further evaluate the docked complexes’ binding conformations and stability.

## 2. Results

### 2.1. Molecular Docking Analysis

For improved energy against viral NS2B/NS3 protease (PDB ID: 2FOM), a total of 27 compounds were chosen, with docking scores ranging from −6.5 kcal/mol to −3.4 kcal/mol. The binding affinity of choerospondin diaplayed (−6.576 kcal/mol) to the target receptor was the highest whereas the control exhibit −5.1 kcal/mol energy. The other compounds displaying top docking scores included nortanshinone (−6.1 kcal/mol), forsythoside A (−6 kcal/mol), luteone (−5.8 kcal/mol), paeonidanin B (−5.6 kcal/mol), phellamuretin (−5.5 kcal/mol), sesamin (−5.3 kcal/mol), asarinin (−5.3 kcal/mol), 6’’-*O*-acetylliquiritin (−5.2 kcal/mol), quercimeritrin (−5.2 kcal/mol), phellodendroside (−5.1 kcal/mol), hydroxytanshinone (−5.1 kcal/mol), methylophiopogonanone A (−5 kcal/mol), narirutin (−5 kcal/mol), and quercetin-7-*O*-rutinoside (−5 kcal/mol). Therefore, the XP score was found higher for cherospondin (−5.1 kcal/mol), angustidine (−6 kcal/mol), forsythoside A (−5.8 kcal/mol), luteon (−5.9 kcal/mol), quercimeritrin (−6 kcal/mol), hydroxytanshinone (−5.4 kcal/mol), phellodendroside (−6.1 kcal/mol), quercetin-7-*O*-rutinoside (−5.9 kcal/mol), phellamuretin (−5.8 kcal/mol), and control (−5.4 kcal/mol), respectively. The majority of the compounds interacted with our target receptor’s Lys74, Leu76, Trp83, Leu85, Asn152, and Ile165 active site residues via hydrogen and hydrophobic contacts. Table 1 and Figure S1 show the docking score and amino acid interactions between the selected drugs and the Dengue virus NS2B/NS3 protease.

### 2.2. Prime MM-GBSA Simulation

**Figure 1:**
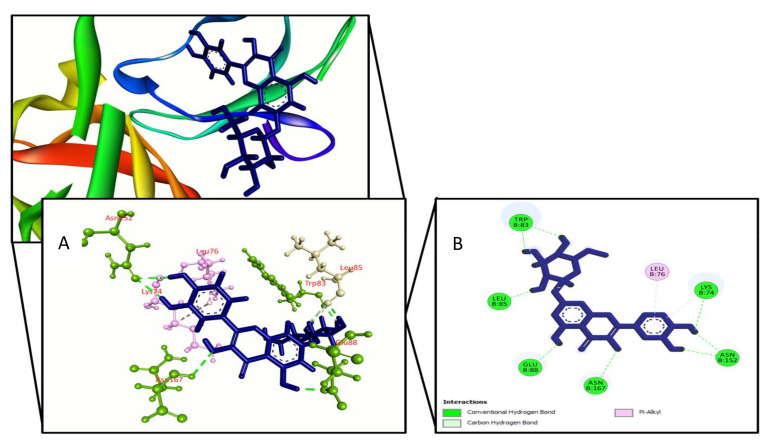
Quercimeritrin binding the Dengue virus NS2B/NS3 protease (PDB: 2FOM), (**A**) 3D representation and (**B**) 2D representation. Hydrogen bonds are displayed as green ball and stick, hydrophobic bonds (Pi–alkyl/alkyl interactions) are displayed as light pink ball and stick, carbon–hydrogen bonds are displayed as white ball and stick.

**Figure 2:**
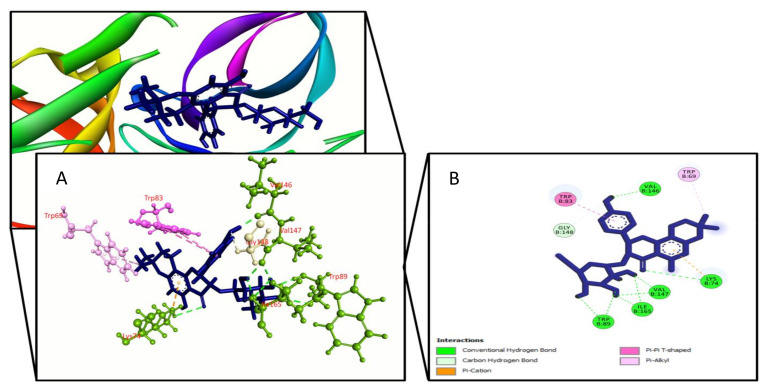
Phellodendroside binding the Dengue virus NS2B/NS3 protease (PDB: 2FOM), (**A**) 3D representation and (**B**) 2D representation. Hydrogen bonds are displayed as green ball and stick, hydrophobic bonds (Pi–Pi/Pi–sigma/amide–Pi interactions) are displayed as deep pink ball and stick, hydrophobic bonds (Pi–alkyl/alkyl interactions) are displayed as light pink ball and stick, carbon–hydrogen bonds are displayed as white ball and stick.

**Figure 3:**
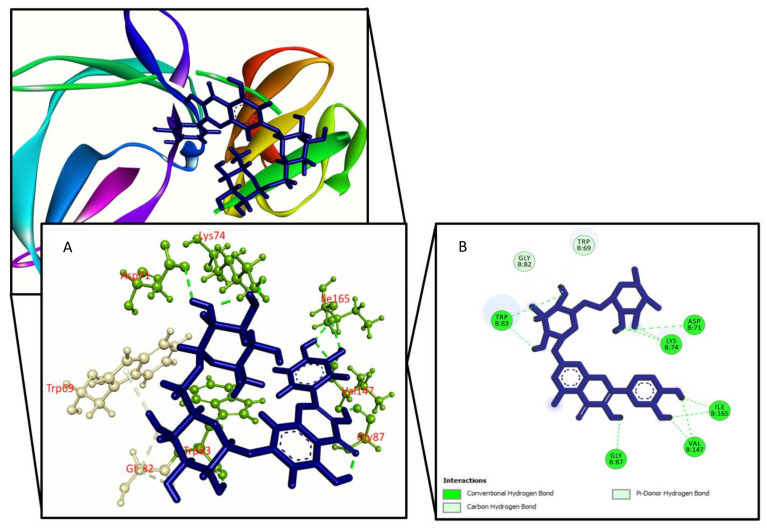
Quercetin-7-*O*-rutinoside binding the Dengue virus NS2B/NS3 protease (PDB: 2FOM), (**A**) 3D representation and (**B**) 2D representation. Hydrogen bonds are displayed as green ball and stick, carbon–hydrogen bonds are displayed as white ball and stick.

**Figure 4:**
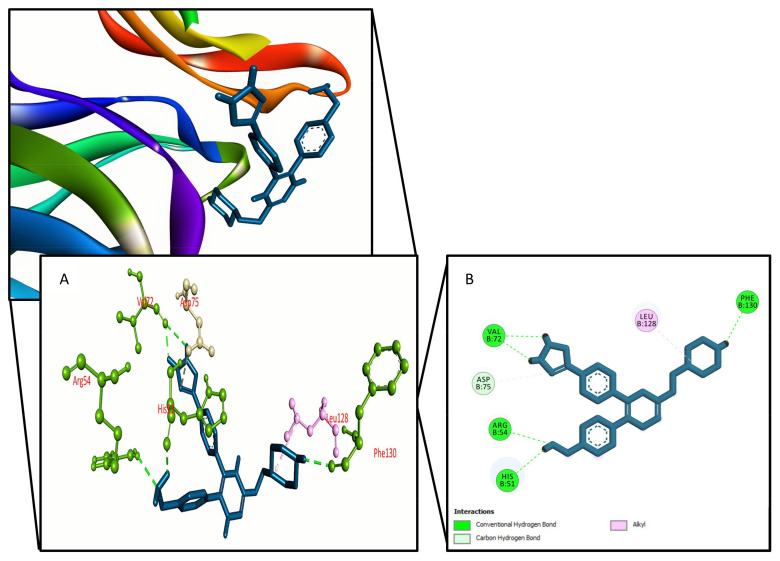
The control ligands binding interactions with the Dengue virus NS2B/NS3 protease, (**A**) 3D representation and (**B**) 2D representation.

We used Prime MM-GBSA attributes to compute the binding energy of the ligands with the Dengue virus NS2B/NS3 protease to assess the clarity of the molecular docking simulation. The binding free energy of a ligand molecule with a lower negative value suggests that it has better binding abilities. The results showed that most of the ligands’ binding energies imply a strong binding engagement with the target receptor. Phellodendroside (−63 kcal/mole), quercimeritrin (−59.5 kcal/mole), and quercetin-7-*O*-rutinoside (−54.1 kcal/mole) are the top ligand–receptor complexes, and the control exhibits a higher binding energy of −51.3 Kcal/mole. Table 1 and Figure 1, Figure 2, Figure 3 and Figure 4 show the results of the binding free energies, three compounds (Figure 1, Figure 2 and Figure 3), and a control (Figure 4) with improved amino acid interactions at the active site of the target protein, respectively.

### 2.3. ADMET Analysis

Pharmacological and toxicity evaluation are critical in assuring the efficacy and safety of lead compounds. The screened compounds were evaluated using the parameters molecular weight, h bond acceptor, h bond donor, CYP2D6 substrate, CYP3A4 substrate, CYP1A2 inhibitor, CYP2C19 inhibitor, CYP2C9 inhibitor, CYP2D6 inhibitor, CYP3A4 inhibitor, AMES toxicity, and oral acute toxicity (Table 2). Phellodendroside, quercimeritrin, and quercetin-7-*O*-rutinoside had molecular weights of 518.51, 464.38, and 610.52g/mol, respectively. There is no CYP inhibition in any of the drugs. In AMES toxicity and oral rat acute toxicity profile, the chemical displays no toxicity.

### 2.4. Molecular Dynamics Simulation

**Figure 5:**
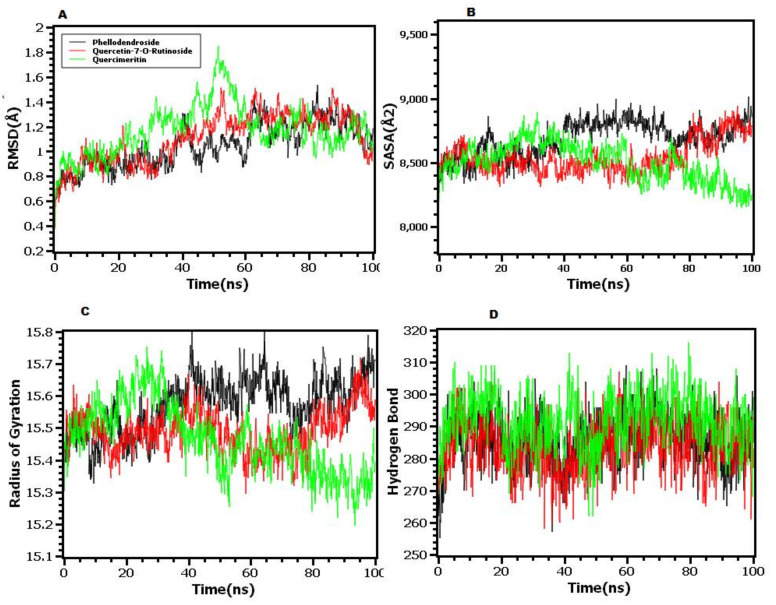
The molecular dynamics simulations study of the docked complexes: (**A**) root mean square deviations, (**B**) solvent accessible surface area, (**C**) radius of gyration, and (**D**) hydrogen bond.

To further understand the stability and stiffness of the docked complexes, molecular dynamics simulations were used. The simulated trajectories were used to calculate the root mean square deviations from the C-alpha atoms of the complexes. Figure 5A demonstrates that at the start of the simulations, phellodendroside, quercetin-7-*O*-rutinoside, and quercimeritin have an upper trend in RMSD, indicating that the complexes were still flexible. The complexes, on the other hand, had established a stable state after 20ns and had maintained their integrity until the simulations’ final images. The quercimeritin displayed a greater RMSD trend and more fluctuations at 40–55 ns periods, indicating the complex’s more flexible character; however, the complexes eventually settled into a stable state. RMSD was less than 2.5 Å for all complexes until the end of the simulations, indicating that the complexes were overall stable.

Furthermore, the solvent accessible surface area (SASA) of the simulated complexes was examined, with a larger trend in SASA indicating the expansion of the protein’s surface area and a lower SASA indicating the complex’s truncated nature. Figure 5B shows that phellodendroside had a larger SASA trend and a higher degree of departures from 60–70 and 80–100 ns compared to other complexes, indicating that when ligand binds to the protein, the surface area expands and the stability decreases. The radius of gyrations from the simulated trajectories was also determined, with the larger Rg indicating a more mobile character of the complexes and the lower Rg indicating a stiffer structure. From 30 ns to the rest of the simulation durations, the complexes of phellodendroside had a greater Rg than the other complexes, indicating that the complexes are more labile (Figure 5C). In simulation trajectories, the other complexes show smaller degrees of divergence.

**Figure 6:**
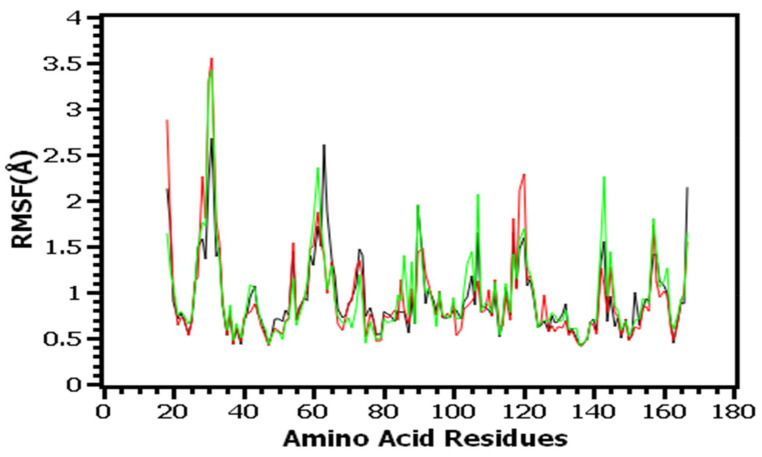
The root mean square fluctuations of the complexes were analyzed to understand the flexibility across the amino acid residues.

In determining the stable state of the complexes, the hydrogen bond plays a crucial role. Figure 5D shows that all complexes showed lower degrees of hydrogen bond patterning aberrations. The root mean square fluctuations (RMSF) of the complexes, which determine the flexibility across the amino acid residues, were also calculated. Figure 6 shows that the maximum residues had an RMSF of less than 2.5, indicating that the complexes were overall stable.

## 3. Discussion

Molecular docking is one of the most widely used methods in computational drug design for predicting the orientation of tiny molecules attached to the binding pocket of an enzyme or receptor [43]. The binding affinities of chosen drugs against Dengue virus NS2B/NS3 protease were investigated using molecular docking simulation in this work. In conjugation with the NS2B cofactor, NS3, a trypsin-like viral protease, is a crucial regulator of viral replication [44]. Potential protease inhibitors derived from Carica papaya leaves interacted with the NS3 protease active site’s Leu149 and conserved substrate-binding residue Asn152. Bioflavonoids extracted from Azadirachta indica, such as rutin, hyperoside, kaempferol-3-*O*-rutinoside, and epicatechin, interacted with the Asn152 residue of the NS3 protease active site [45]. Lys74, Leu76, Trp83, Leu85, Glu88, Ile165, Ala166, and Asn167 active site residues of the dengue NS2B/NS3 protease were found to be involved in hydrophobic and hydrogen bond formation in a recent study based on antiviral phytochemicals [46]. Similar interactions were also found for a few amino acid residues in the dengue NS2B/NS3 protease active site (Lys74, Asn152, and Gln167) in several experimentally tested synthetic compounds [47]. Three compounds were chosen for this study: quercimeritrin, phellodendroside, and quercetin-7-*O*-rutinoside, based on their amino acid interactions, docking scores, and binding free energies. Quercimeritrin interacted with the NS3 protease active site residues Lys74, Leu76, Trp83, Leu85, Glu88, Asn152, Asn167, whereas phellodendroside interacted with Trp69, Lys74, Trp83, Trp89, Val146, Val147, Gly148, Ile165. Quercetin-7-*O*-rutinoside established hydrogen bonds with the NS2B/NS3 protease residues Asp71, Lys74, Trp83, Gly87, Val147, and Ile165. Multiple hydrogen bonds were formed between the control ligand molecules and the target protein at Val72, His51, Arg54, and Phe130, as well as one hydrophobic interaction at Leu128.

Multiple interactions were also observed at Asn152 and Lys74 by benzothiazole derivatives in molecular modeling studies, and their inhibition of replications was validated in vitro and in a cell-based PR assay for distinct Dengue serotypes [48]. Benzimide also generated hydrogen-bond interactions at Asn152, and the hydrophobic side of the benziminazole ring immediately attached to engage with Lys74 and Leu76, which are identical to the interactions of the top three compounds in this study [49]. In recent fragment-based drug design efforts against Dengue virus NS2B/NS3 revealed that Asn167, Leu85, and Glu88 were important in generating non-covalent interactions with the NS2B/NS3 protein [50]. Our screened compounds’ interaction patterns were also shown to be similar for papraine A, robustune, eryvarin, sigmodin, osaji, and laburnetin [51].

Based on the findings of the previous investigations, these amino acid interactions showed that these compounds could be effective as dengue NS2B/NS3 protease inhibitors. Although molecular docking is a reliable method for predicting a small molecule’s binding poses, it still requires additional validation to appropriately rank the ligands’ affinities to the target receptor. We used Prime MM/GBSA analysis to verify the docking experiments, which is a revolutionary QM/MM property for calculating relative binding affinities using the best poses from ligand-receptor interactions [52]. Within the binding site of the Dengue virus NS2B/NS3 protease, phellodendroside has the highest MM-GBSA binding energy of approximately −63 kcal/mole. The binding free energies of quercimeritrin and quercetin-7-*O*-rutinoside were also acceptable, confirming the results of our docking experiment.

In addition, a molecular dynamics simulation investigation was carried out for further docking conformation validations [53,54,55]. For a better understanding of the docked complexes’ stable nature, numerous characteristics from simulated trajectories were evaluated. The RMSD, RMSF, Rg, hydrogen bond, and SASA define the complexes and show that they are more stable. As a result, the complexes’ pharmacological properties were investigated to assure their safety and toxicity level [56,57,58]. To be used as drug candidates, substances must have particular drug-like qualities and have a low level of toxicity [59,60,61]. In ADMET calculations, the top three compounds show no toxicity profiling and have favorable drug similarity features.

**Figure 7:**
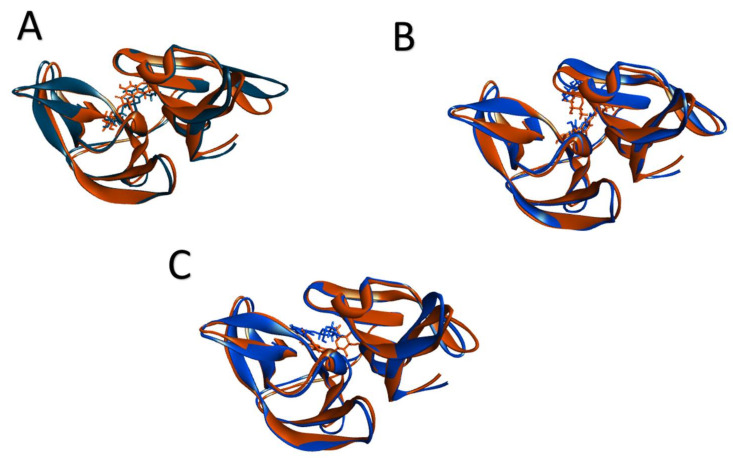
The superimposition of the pre and post-MD docked complexes; (**A**) phellodendroside, (**B**) quercetin-7-*O*-rutinoside, (**C**) quercimeritrin complexes, here blue indicates the pre-MD docked complexes, and orange indicates post-MD docked complexes.

In addition, the MM-GBSA binding free energy following molecular dynamics simulations shows that the complex; quercimeritrin, phellodendroside, and quercetin-7-*O*-rutinoside exhibited binding free energies of −51.3Kcal/mol, −54.3Kcal/mol, and −52.7Kcal/mol, respectively. The superimpositions of the pre- and post-MD docked structures revealed (Figure 7) that the phellodendroside, quercetin-7-*O*-rutinoside, and quercimeritrin complexes had RMSD of 1.186, 1.01, and 1.04, respectively, indicating that the docked complexes had less structural deviations during molecular dynamics simulations.

The top three phytochemicals may have the ability to interfere with the operation of Dengue virus NS2B/NS3 protease, according to combinatorial bioinformatics methodologies such as molecular dynamics and molecular dynamics simulations. This work also tabulated fresh phytochemical information, which will aid researchers in using additional targeted viral proteins or signaling molecules. Although the data in this work were generated exclusively through computational methods, they still have to be confirmed in a wet lab setting using several enzymatic assays.

## 4. Material and Methods

### 4.1. Ligand Retrieval and Preparation

Based on the literature research, about 2000 phytochemical substances were collected from the Pubchem database [62] (Table S1, Supplementary Materials). The structures were neutralized using Epik 2.2 at pH 7.0 ± 2.0, and the force field OPLS 2005 contained in Maestro, version 10.1 (Schrödinger suite) [63] was used to reduce them. Per ligand, up to 32 stereoisomers were preserved.

### 4.2. Protein Preparation

The RCSB PDB [64,65] provided three-dimensional crystallographic structures of the proteins utilized in this study: Dengue virus NS2B/NS3 protease (PDB ID: 2FOM) [66]. To prepare and modify crystallographic structures for molecular docking experiments, the Protein Preparation Wizard of Maestro version 10.1 (Schrödinger suite) was used. Hydrogens were added to the heavy atoms, selenomethionines were transformed to methionines, and all waters were removed. Minimization was carried out using the force field OPLS 2005, with the maximum heavy atom RMSD (root mean square deviation) set to 0.30 [67,68].

### 4.3. Virtual Screening

For virtual screening, the Glide software from the Schrodinger working flow was used, which offers different docking protocols, HTVS (high-throughput virtual screening), standard precision, and SP. The best compounds from the pool were identified using the prior techniques. Every ligand was docked against the receptor using HTVS, which results in a single posture. About half of all plant-derived chemicals were shifted from HTVS to SP, reducing the number of false positives [69,70]. Here, 1-(4-{5-[(piperidin-4-yl)methoxy]-3-[4-(1*H*-pyrazol-4-yl)phenyl]pyrazin-2-yl}phenyl)methanamine was used a control ligand molecules [71].

### 4.4. Prime MM-GBSA Simulation

Based on SP score, a total of 27 biological compounds were chosen for MM-GBSA study against Dengue virus NS2B/NS3 protease. The binding energies of the ligands with the Dengue virus NS2B/NS3 protease were calculated using the Prime MM-GBSA (Molecular Mechanics/Generalized Born Surface Area) technique. The prime module of the Schrödinger suite, Maestro version 10.1, was used for this. The MM-GBSA technique combines the OPLSAA molecular mechanics energies (EMM), a surface generalized Born (SGB) model for polar solvation (GSGB), and a non-polar solvation term (GNP) made up of non-polar solvent accessible surface area and van der Waals interactions [72]. Here, the Glide pose viewer file of the best conformation was given as the source in Prime MM-GBSA simulation [73]. The total free energy of binding:

ΔG_bind_ = G_complex_ − (G_protein_ + G_ligand_), where G = EMM + GSGB + GNP (1)

### 4.5. ADMET Analysis

SwissADME [74], admetSAR [75], and pkCSM [76] were used to calculate the pharmacological and pharmacokinetic parameters of the three compounds. As an entry system, the ligand molecules’ canonical simplified molecular input line entry system (SMILES) was used.

### 4.6. Molecular Dynamics Simulation

Molecular dynamics simulations of docked complexes were carried out using YASARA dynamics [77] and the AMBER14 force field [78]. Initial cleaning, optimization, and orientation of hydrogen bond networks were performed on docked complexes. In a cubic simulation cell with periodic boundary conditions, the TIP3P water solvation model was used [79]. The simulation cell’s physiological conditions were set at 298K, pH 7.4, and 0.9% NaCl. The particle mesh Ewalds (PME) approach was used to calculate long-range electrostatic interactions with a cutoff radius of 8.0 [80,81,82]. The steepest gradient techniques were used to implement the initial energy minimization procedure using the simulated annealing approach (5000 cycles). The simulation’s time step was set to 2.0 fs [83]. After every 100 ps period, the simulation trajectories were saved. The simulation was extended for 100 ns by using a constant pressure and Berendsen thermostat, and trajectories were used to compute the root mean square deviation (RMSD), root mean square fluctuations (RMSF), solvent accessible surface area (SASA), radius of gyration (Rg), and hydrogen bond [84,85,86,87].

## 5. Conclusions

To select the effective inhibitors from the compounds, this study used structure-based screening of phytochemicals. As a result, hundreds of phytochemicals have been identified in plants, particularly in Asian species. Based on greater binding affinity and MM-GBSA binding free energy, the top three compounds were chosen: phellodendroside (−63 kcal/mole), quercimeritrin (−59.5 kcal/mole), and quercetin-7-*O*-rutinoside (−54.1 kcal/mole). As a result, a molecular dynamics analysis was carried out to confirm the structural stability and binding pose. The toxicity and carcinogenicity of the tested molecules shows no possible adverse effect of the compounds. This study relies primarily on computational screening and pipelines; nonetheless, these studies must be confirmed in the wet lab using several enzymatic assays.

## Figures and Tables

**Table 1:** The binding energy and interacting residues of the best 27 phytochemicals, the interactions were analyzed in the Discovery Studio and Pymol package.

Compound Name	Docking Score (Kcal/mol)	XP Score (Kcal/mol)	ΔG_bind_ (kcal/mol)	Hydrogen BondInteractions	Hydrophobic Bonds (Pi–Alkyl/Alkyl)	Hydrophobic Bonds (Pi–Pi/Pi–Sigma/Pi–Cation/Pi–Anion/Pi–Amide)	Hydrophobic Bonds (Pi–Sulfur/Carbon–Hydrogen)
Rutaecarpine	−4.2	−3.7	−39.8	Trp83	Leu76	Ile165	-
Sesamin	−5.3	−4	−45.1	Lys74, Leu149	Leu76	-	Leu149, Ala164
Choerospondin	−6.3	−5.1	−47.9	Lys74, Glu88, Leu149, Asn152, Ala164	Leu76	Trp83, Ile165	-
Evodiamine	−4.7		−39	Leu85	Leu76, Trp83, Leu85	Glu88	Leu85, Gly87, Val146
		−3.1					

Narirutin	−5	−3.6	−30.7	Lys74, Trp83, Leu85, Gly87, Glu88, Asn152	-	-	Leu85, Glu86
Angustidine	−4.6	−6	−45.1	Trp83, Leu149	Leu76, Ala166	-	Gly87
Forsythoside A	−6	−5.8	−34.6	Glu43, Lys74, Glu86, Gly87, Glu88, Val146	Val147	-	Trp83
Luteone	−5.8	−5.9	−51.1	Lys74, Leu85, Asn152, Asn167	-	Trp83, Ile165	Val147
**Quercimeritrin**	**−5.2**	**−6**	**−59.5**	**Lys74, Trp83, Leu85, Glu88, Asn152, Asn167**	**Lys74, Leu76**	**-**	**Trp83, Leu85**
Hydroxytanshinone	−5.1	−5.4	−49.6	Trp83, Gly87, Leu149	Leu76, Ala164	Ile165	Asn152
Methylophiopogonanone A	−5	−5.1	−49.1	Trp83, Asn152, Ala164	Leu76	Ile165	Gly148, Ile165
Tanshinlactone	−4.6	−3.5	−40.2	Trp83	Leu76, Ala164	-	Glu88, Gly148
Asarinin	−5.3	−4.2	−35.9	Lys74, Leu149, Asn167	Lys74, Leu76	-	Trp83, Gly148, Ala164, Ile165
Piperitylhonokiol	−4.6	−4.7	−52.5	Leu149, Ala164, Ile165	Trp69, Lys74, Leu76	Lys74, Trp83, Ile165	-
Sanjoinine B	−4.3	−4.7	−48.3	Glu88, Asn167	Trp83, Ala166	-	Ile165, Ala166
Sanjoinine D	−3.7	−5	−48.4	Lys74, Glu88	Trp83, Ala166	-	Glu88
Scutianine D	−3.4	−5.1	−44.4	Lys74, Asn167	Trp83	Lys74, Trp83	Trp83, Ile165, Ala166
Scutianine C	−4.1	−4.6	−42.9	Lys74, Asn167	Ala166	Glu88, Lys90	Ala166
6’’-*O*-Acetylliquiritin	−5.2	−5.4	−52.2	Lys74, Glu88, Asn152	Lys74, Leu76	Lys74, Trp83, Ile165	Glu88, Gly148
**Phellodendroside**	**−5.1**	**−6.1**	**−63**	**Lys74, Trp89, Val146, Val147, Ile165**	**Trp69**	**Lys74, Trp83**	**Gly148, Ile165**
**Quercetin-7-*O*-Rutinoside**	**−5**	**−5.9**	**−54.1**	**Asp71, Lys74, Trp83, Gly87, Val147, Ile165**	**-**	**-**	**Trp69, Gly82**
(*S*)-Suspensaside	−4.7	−3.5	−28.2	Lys74, Trp83, Gly87, Glu88, Asn167	-	-	Trp69, Leu85, Glu86, Glu88, Val147, Gly148
Paeonidanin B	−5.6	−5.3	−47.1	Lys74, Leu85, Gly87, Asn152, Ala164, Asn167	Lys74, Leu76	-	Leu85, Gly87, Gly148
Phellamuretin	−5.5	−5.8	−42.9	Lys74, Trp89, Lys90, Asn167	Ala166	Glu88	Trp83
Nortanshinone	−6.1	−5.3	−44.1	Trp83, Leu149, Asn167	Leu76, Ala164	Ile165	Leu85
Sec-*O*-Glucosylhamaudol	−4.4	−4.6	−45.7	Trp83, Leu85, Leu149	Leu76, Ala164	Ile165	Trp83, Leu85, Gly148
Benzoyloxypaeoniflorin	−4.9	−5.5	−37.8	Lys74, Trp83, Gly87, Asn152	Lys74, Leu76, Ala166	Ile165	-
**Control**	**−5.1**	**−5.4**	**−51.3**	**Phe130, Val72** **Arg54, His51**	**Leu128**	**-**	**-**

**Table 2:** The pharmacological and toxicity profiling of the screened compounds from SwissADME, ADMETSAR and PKCSM tools.

Parameter	Phellodendroside	Quercimeritrin	Quercetin-7-*O*-Rutinoside
**Molecular weight**	518.51g/mol	464.38g/mol	610.52g/mol
**H bond acceptor**	11	12	16
**H bond donor**	6	8	10
**CYP2D6 substrate**	No	No	No
**CYP3A4 substrate**	No	No	No
**CYP1A2 inhibitor**	No	No	No
**CYP2C19 inhibitor**	No	No	No
**CYP2C9 inhibitor**	No	No	No
**CYP2D6 inhibitor**	No	No	No
**CYP3A4 inhibitor**	No	No	No
**AMES toxicity**	No	No	No
**Oral rat acute toxicity (LD50)**	2.957 (mol/kg)	2.20 (mol/kg)	2.53 (mol/kg)

## Data Availability

The data presented in this study are available in this article and supplementary material.

## Supplementary Materials

The following are available online, Figure S1: 2D representation of the selected compounds against Dengue Virus NS2B/NS3 Protease (PDB: 2FOM). (A) Rutaecarpine, (B) Sesamin, (C) Choerospondin, (D) Evodiamine, (E) Narirutin, (F) Angustidine, (G) Forsythoside A, (H) Luteone, (I) Quercimeritrin, (J) Hydroxytanshinone, (K) Methylophiopogonanone A, (L) Tanshinlactone, (M) Piperitylhonokiol, (N) Sanjoinine B, (O) Sanjoinine D, (P) Scutianine D, (Q) Scutianine C, (R) 6’’-*O*-Acetylliquiritin, (S) Phellodendroside, (T) Quercetin-7-*O*-Rutinoside, (U) (*S*)-Suspensaside, (V) Paeonidanin B, (W) Phellamuretin, (X) Nortanshinone, (Y) Sec-*O*-Glucosylhamaudol, (Z) Benzoyloxypaeoniflorin, and (AA) Asarinin. Table S1: Selected plants, their compounds and CID of each compound.

## Sample Availability

Samples of the compounds are available from the authors.
