# Supplementary material for: Phytochemical Compound Screening to Identify Novel Small Molecules against Dengue Virus: A Docking and Dynamics Study
Source: Molecules. 2022 Jan 20;27(3):653. doi: 10.3390/molecules27030653 (PMC8840231; doi:10.3390/molecules27030653)
Supplement: Supplementary file 1 [file molecules-27-00653-s001.zip › molecules-1533007-supplementary.pdf]

molecules MDPI

*Supplementary Materials*

# Phytochemical Compound Screening to Identify Novel Small Molecules Against Dengue Virus: A Docking and Dynamics Study

**Mst. Sharmin Sultana Shimu [1,†], Shafi Mahmud [2,†], Trina Ekwati Tallei [3], Saad Ahmed Sami [4], Ahmad Akroman Adam [5], Uzzal Kumar Acharjee [1,*], Gobindo Kumar Paul [2], Talha Bin Emran [6], Shahriar Zaman [2], Md. Salah Uddin [2] , Md. Abu Saleh [2,*], Sultan Alshehri[7], Mohammed M Ghoneim[8], Maha Alruwali[8], Ahmad J. Obaidullah[9,10], Nabilah Rahman Jui[11], Junghwan Kim[12], and Bonglee Kim[13]**

[1] Genetic Engineering and Biotechnology, University of Rajshahi, Rajshahi-6205, Bangladesh; sharminshimu120@gmail.com.
[2] Microbiology Laboratory, Genetic Engineering and Biotechnology, University of Rajshahi, Rajshahi-6205, Bangladesh; shafimahmudfz@gmail.com (S.M.); gobindokumar38@gmail.com (G.K.P.); szaman@ru.ac.bd (S.Z.); salim.geb@ru.ac.bd (M.S.U.);
[3] Department of Biology, Faulty of Mathematics and Natural Science, Sam Ratulangi University, Manado, North Sulawesi 95115, Indonesia; trina_tallei@unsrat.ac.id
[4] Department of Pharmacy, University of Chittagong, Chittagong, Bangladesh; s.a.sami18pharm@gmail.com
[5] Dentistry Study Program, Faculty of Medicine, Sam Ratulangi University, Indonesia; ahmad_adam@ymail.com
[6] Department of Pharmacy, BGC Trust University, Chittagong, Bangladesh; talhabmb@bgctub.ac.bd
[7] Department of Pharamaceutics, College of Pharmacy, King Saud University, Riyadh 11451, Saudi Arabia
[8] Department of Pharmacy Practice, College of Pharamcy, AlMaarefa University, Ad Diriyah 13713, Saudi Arabia
[9] Drug Exploration and Development Chair (DEDC), Department of Pharmaceutical Chemistry, College of Pharmacy, King Saud University, Riyadh 11451, Saudi Arabia.
[10] Department of Pharmaceutical Chemistry, College of Pharmacy, King Saud University, Riyadh 11451, Saudi Arabia
[11] Department of Biochemistry and Biotechnology, University of Science and Technology Chittagong, Bangladesh
[12] Department of Internal Medicine, College of Korean Medicine, Kyung Hee University, Seoul, 02447, Republic of Korea; akom21@khu.ac.kr (J.K.)
[13] Department of Pathology, College of Korean Medicine, Kyung Hee University, Hoegidong Dongdaemungu, Seoul, 05253, Republic of Korea. bongleekim@khu.ac.kr (B.K.)
† These authors contributed equally to this work.
* Correspondence: ukarcc@gmail.com (U.K.A.); saleh@ru.ac.bd (M.A.S.); bongleekim@khu.ac.kr (B.K.)

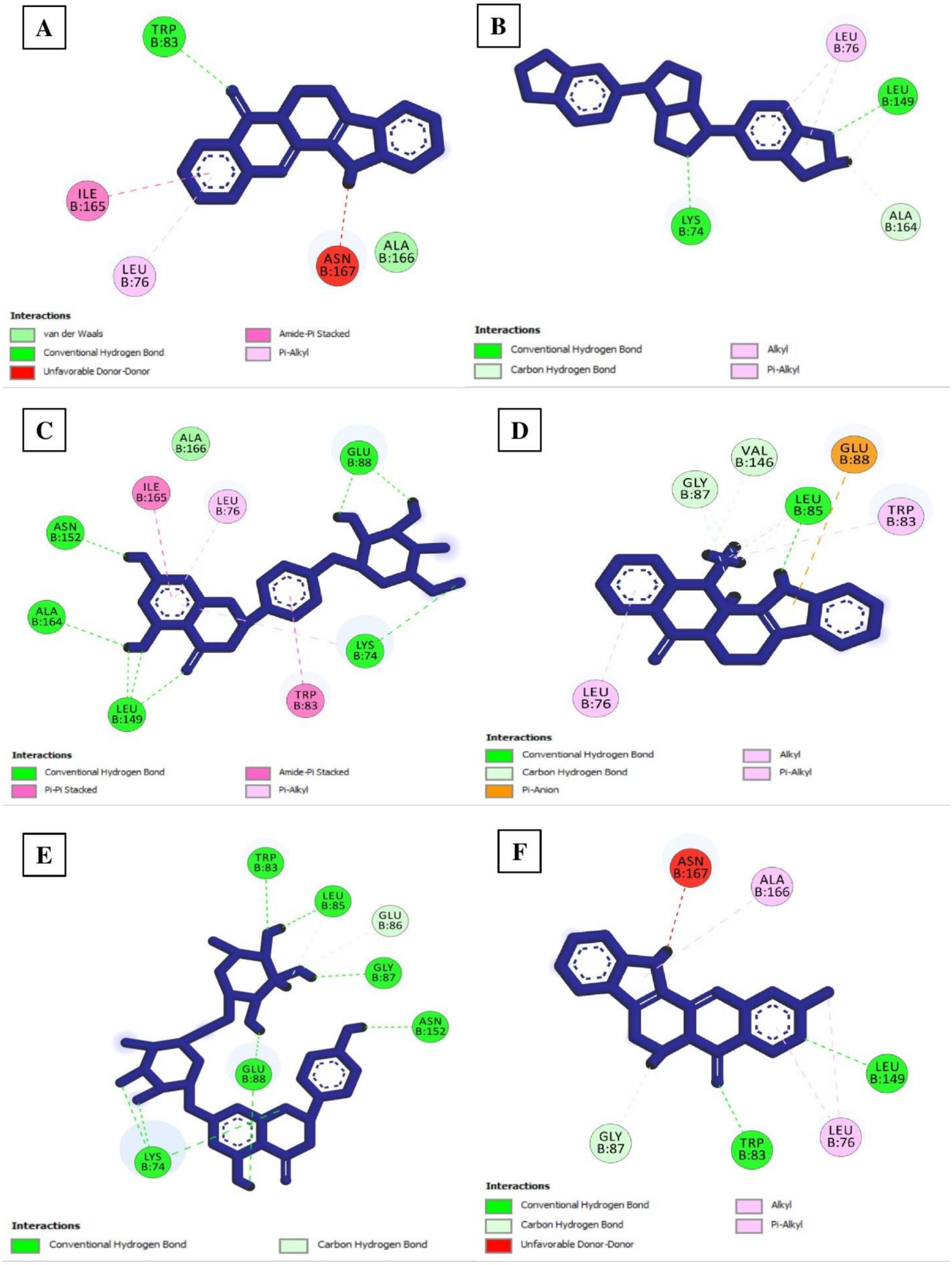
A
TRP B:83
ILE B:165
LEU B:76
ASN B:167
ALA B:166
Interactions
van der Waals
Conventional Hydrogen Bond
Unfavorable Donor-Donor
Amide-Pi Stacked
Pi-Alkyl
B
LEU B:76
LEU B:149
LYS B:74
ALA B:164
Interactions
Conventional Hydrogen Bond
Carbon Hydrogen Bond
Alkyl
Pi-Alkyl
C
ALA B:166
GLU B:88
ILE B:165
LEU B:76
ASN B:152
ALA B:164
LYS B:74
TRP B:83
LEU B:149
Interactions
Conventional Hydrogen Bond
Pi-Pi Stacked
Amide-Pi Stacked
Pi-Alkyl
D
VAL B:146
GLU B:88
GLY B:87
LEU B:85
TRP B:83
LEU B:76
Interactions
Conventional Hydrogen Bond
Carbon Hydrogen Bond
Pi-Anion
Alkyl
Pi-Alkyl
E
TRP B:83
LEU B:85
GLU B:86
GLY B:87
ASN B:152
GLU B:88
LYS B:74
Interactions
Conventional Hydrogen Bond
Carbon Hydrogen Bond
F
ASN B:167
ALA B:166
LEU B:149
GLY B:87
TRP B:83
LEU B:76
Interactions
Conventional Hydrogen Bond
Carbon Hydrogen Bond
Unfavorable Donor-Donor
Alkyl
Pi-Alkyl

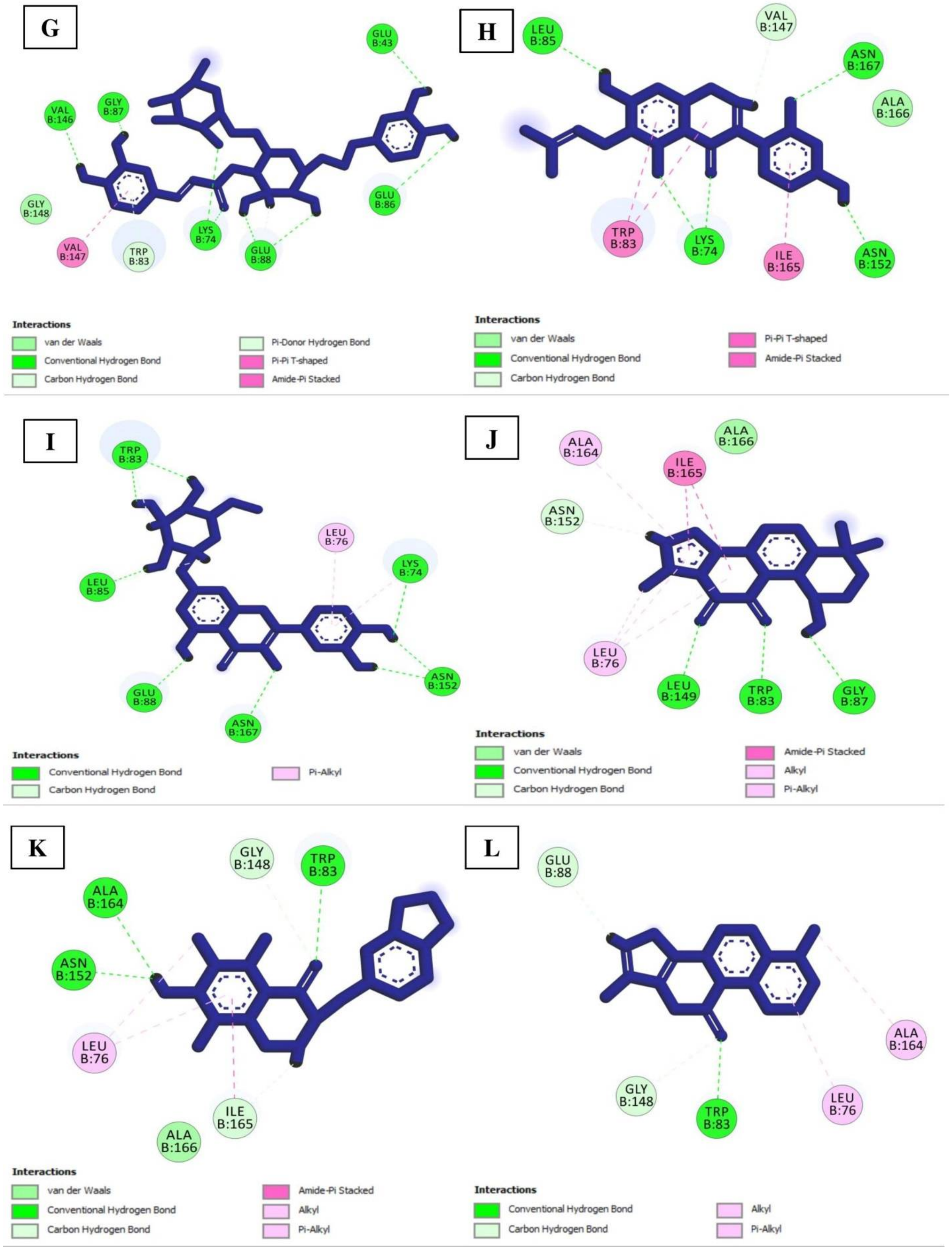
G
GLU B:43
VAL B:146
GLY B:87
GLY B:148
GLU B:86
VAL B:147
TRP B:83
LYS B:74
GLU B:88
Interactions
van der Waals
Conventional Hydrogen Bond
Carbon Hydrogen Bond
Pi-Donor Hydrogen Bond
Pi-Pi T-shaped
Amide-Pi Stacked
H
LEU B:85
VAL B:147
ASN B:167
ALA B:166
TRP B:83
LYS B:74
ILE B:165
ASN B:152
Interactions
van der Waals
Conventional Hydrogen Bond
Carbon Hydrogen Bond
Pi-Pi T-shaped
Amide-Pi Stacked
I
TRP B:83
LEU B:76
LYS B:74
LEU B:85
ASN B:152
GLU B:88
ASN B:167
Interactions
Conventional Hydrogen Bond
Carbon Hydrogen Bond
Pi-Alkyl
J
ALA B:164
ALA B:166
ILE B:165
ASN B:152
LEU B:76
LEU B:149
TRP B:83
GLY B:87
Interactions
van der Waals
Conventional Hydrogen Bond
Carbon Hydrogen Bond
Amide-Pi Stacked
Alkyl
Pi-Alkyl
K
GLY B:148
TRP B:83
ALA B:164
ASN B:152
LEU B:76
ILE B:165
ALA B:166
Interactions
van der Waals
Conventional Hydrogen Bond
Carbon Hydrogen Bond
Amide-Pi Stacked
Alkyl
Pi-Alkyl
L
GLU B:88
ALA B:164
GLY B:148
TRP B:83
LEU B:76
Interactions
Conventional Hydrogen Bond
Carbon Hydrogen Bond
Alkyl
Pi-Alkyl

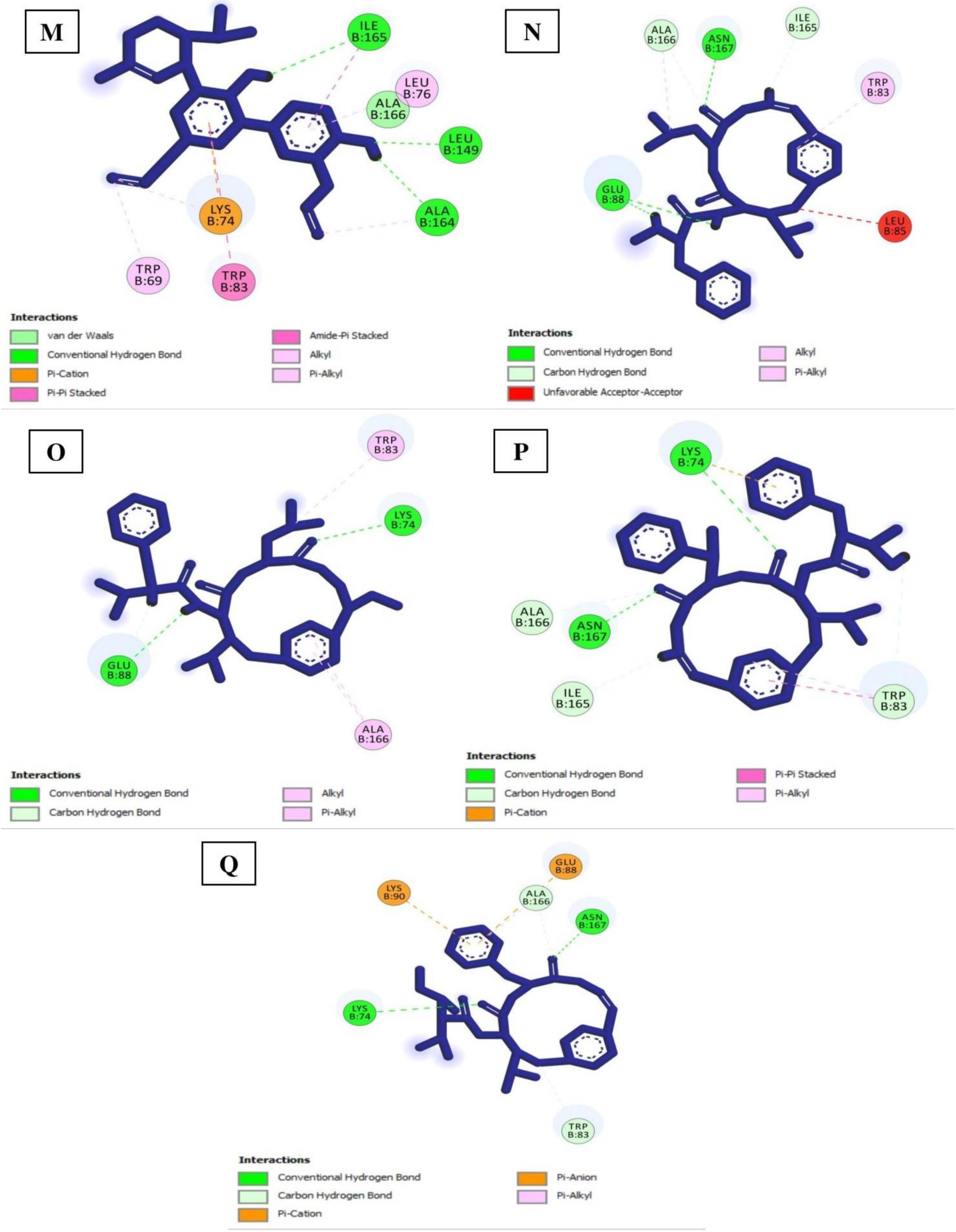
M
ILE B:165
LEU B:76
ALA B:166
LEU B:149
ALA B:164
LYS B:74
TRP B:69
TRP B:83
Interactions
van der Waals
Conventional Hydrogen Bond
Pi-Cation
Pi-Pi Stacked
Amide-Pi Stacked
Alkyl
Pi-Alkyl
N
ALA B:166
ASN B:167
ILE B:165
TRP B:83
GLU B:88
LEU B:85
Interactions
Conventional Hydrogen Bond
Carbon Hydrogen Bond
Unfavorable Acceptor-Acceptor
Alkyl
Pi-Alkyl
O
TRP B:83
LYS B:74
GLU B:88
ALA B:166
Interactions
Conventional Hydrogen Bond
Carbon Hydrogen Bond
Alkyl
Pi-Alkyl
P
LYS B:74
ALA B:166
ASN B:167
ILE B:165
TRP B:83
Interactions
Conventional Hydrogen Bond
Carbon Hydrogen Bond
Pi-Cation
Pi-Pi Stacked
Pi-Alkyl
Q
GLU B:88
LYS B:90
ALA B:166
ASN B:167
LYS B:74
TRP B:83
Interactions
Conventional Hydrogen Bond
Carbon Hydrogen Bond
Pi-Cation
Pi-Anion
Pi-Alkyl

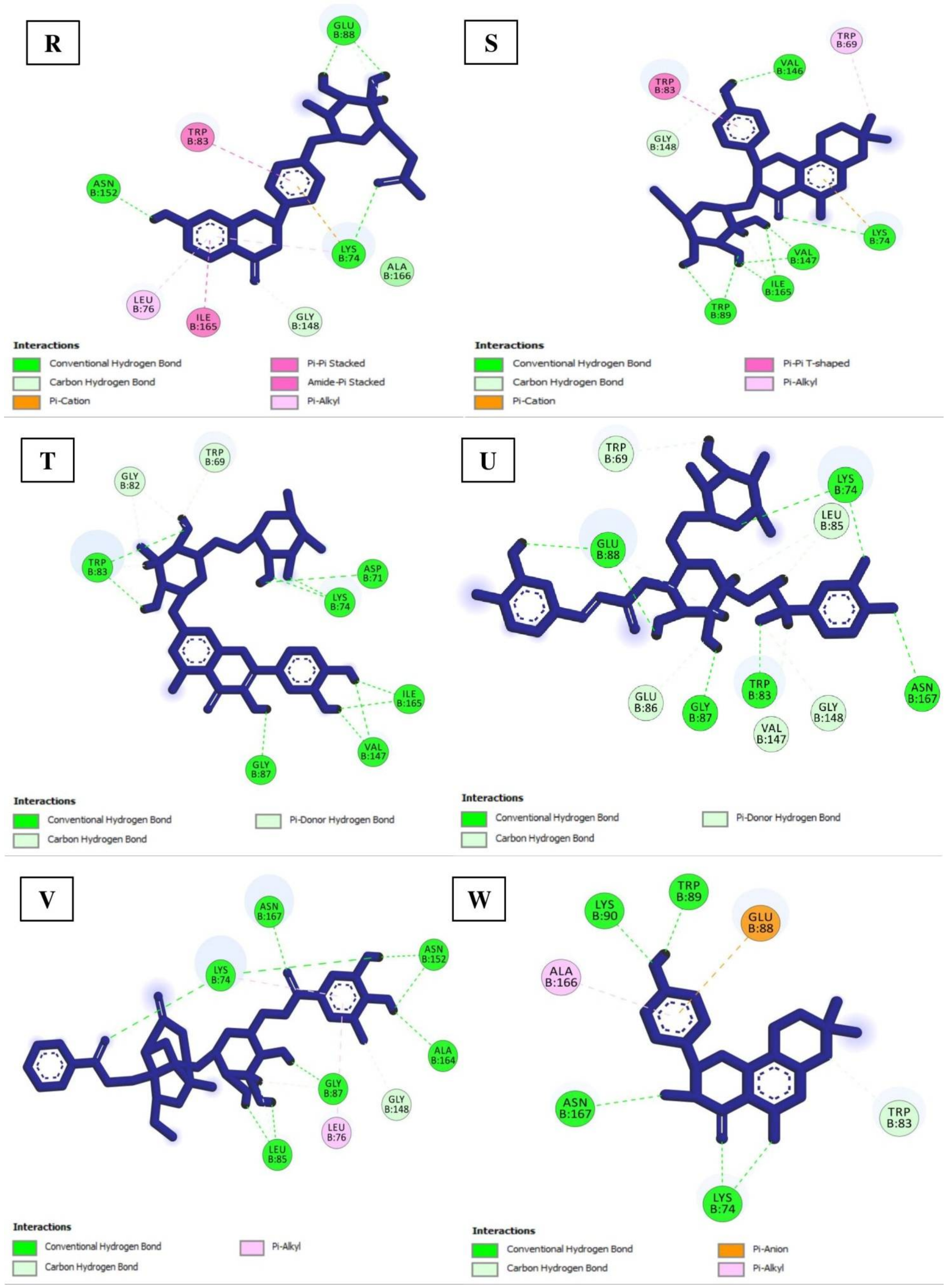
R
GLU B:88
TRP B:83
ASN B:152
LYS B:74
ALA B:166
LEU B:76
ILE B:165
GLY B:148
Interactions
Conventional Hydrogen Bond
Carbon Hydrogen Bond
Pi-Cation
Pi-Pi Stacked
Amide-Pi Stacked
Pi-Alkyl
S
TRP B:69
VAL B:146
TRP B:83
GLY B:148
LYS B:74
VAL B:147
ILE B:165
TRP B:89
Interactions
Conventional Hydrogen Bond
Carbon Hydrogen Bond
Pi-Cation
Pi-Pi T-shaped
Pi-Alkyl
T
TRP B:69
GLY B:82
TRP B:83
ASP B:71
LYS B:74
ILE B:165
VAL B:147
GLY B:87
Interactions
Conventional Hydrogen Bond
Carbon Hydrogen Bond
Pi-Donor Hydrogen Bond
U
TRP B:69
LYS B:74
LEU B:85
GLU B:88
GLU B:86
GLY B:87
TRP B:83
VAL B:147
GLY B:148
ASN B:167
Interactions
Conventional Hydrogen Bond
Carbon Hydrogen Bond
Pi-Donor Hydrogen Bond
V
ASN B:167
ASN B:152
LYS B:74
ALA B:164
GLY B:87
GLY B:148
LEU B:76
LEU B:85
Interactions
Conventional Hydrogen Bond
Carbon Hydrogen Bond
Pi-Alkyl
W
LYS B:90
TRP B:89
GLU B:88
ALA B:166
ASN B:167
TRP B:83
LYS B:74
Interactions
Conventional Hydrogen Bond
Carbon Hydrogen Bond
Pi-Anion
Pi-Alkyl

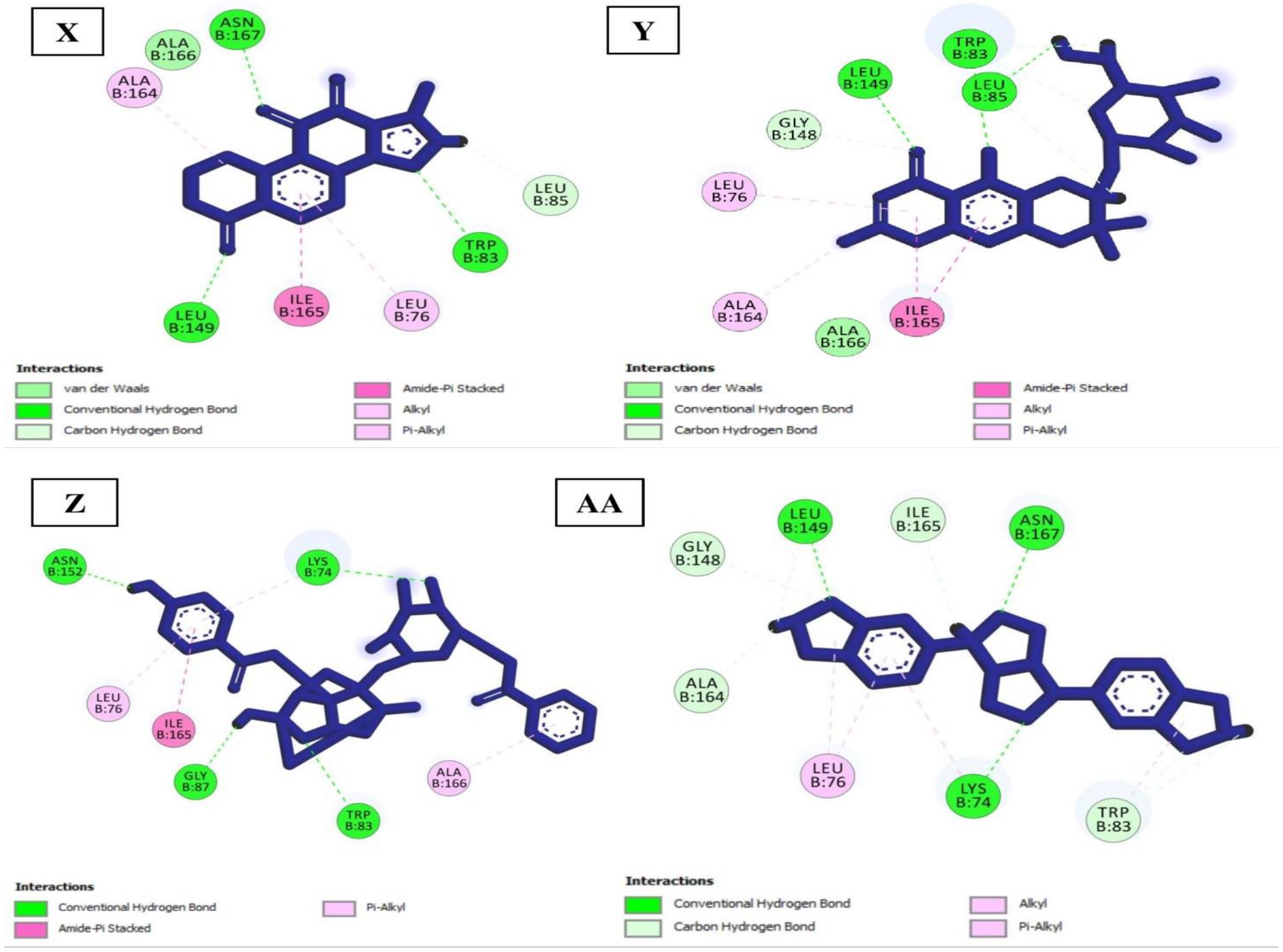


**Figure S1.** 2D representation of the selected compounds against Dengue Virus NS2B/NS3 Protease (PDB: 2FOM). (**A**) Rutaecarpine, (**B**) Sesamin, (**C**) Choerospondin, (**D**) Evodiamine, (**E**) Narirutin, (**F**) Angustidine, (**G**) Forsythoside A, (**H**) Luteone, (**I**) Quercimeritrin, (**J**) Hydroxytanshinone, (**K**) Methylophiopogonanone A, (**L**) Tanshinlactone, (**M**) Piperitylhonokiol, (**N**) Sanjoinine B, (**O**) Sanjoinine D, (**P**) Scutianine D, (**Q**) Scutianine C, (**R**) 6''-*O*-Acetylliquiritin, (**S**) Phellodendroside, (**T**) Quercetin-7-*O*-Rutinoside, (**U**) (*S*)-Suspensaside, (**V**) Paeonidanin B, (**W**) Phellamuretin, (**X**) Nortanshinone, (**Y**) Sec-*O*-Glucosylhamaudol, (**Z**) Benzoyloxypaeoniflorin, and (**AA**) Asarinin.

**Table S1.** Selected plants, their compounds and CID of each compound.

| no | Name | Compounds | CID | Reference |
|---|---|---|---|---|
| | | Methylpluviatilol | 5320622 | |
| | | Pluviatilol | 70695727 | |
| | | Epipinoresinol | 637584 | |
| | | Sesamin | 72307 | |
| | | Piperitol | 10247670 | |
| | | Methyleugenol | 7127 | |
| | | (±)-car-3-ene-2,5-dione | 181910 | |
| | | -N-isobutyl-2,4,8,10-dodecatetraenamide | 57404464 | |
| | | Myristicin | 4276 | |
| | | Safrole | 5144 | |
| | | Asarinol A | 46174001 | |
| | | Asarinol B | 46174002 | |
| | | Asarinol C | 21631051 | |
| | | (+)-Asarinol D | 21631052 | |
| | | 3-Caren-2-one | 13901618 | |
| | | m-Cymene | 10812 | |
| | | p-Cymene | 7463 | |
| | | p-Cymen-7-ol | 325 | 1 |
| | | 2,3-Dehydro-1,8-cineol | 523035 | 1 |
| | | Fenchone | 14525 | 1 |
| | | Myrcenol | 10975 | 1 |
| | Asarum sieboldii | Phellandral | 89488 | 1 |
| 1. | | Sabinyl acetate | 94266 | 1 |
| | (45) | Terpinen-4-yl acetate | 20960 | 1 |
| | | Calarene | 28481 | 1 |
| | | Alpha-Guaiene | 5317844 | 2 |
| | | Beta-Guaiene | 6949 | 2 |
| | | Isoledene | 530426 | 3 |
| | | Sativene | 11830550 | |
| | | 3',4'-Dimethoxycinnamaldehyde | 5375268 | |
| | | 3,5-Dimethoxytoluene | 77844 | |
| | | 2,3,5-Trimethoxytoluene | 170114 | |
| | | Asaricin | 4276 | |
| | | Alpha-Asarone | 636822 | |
| | | Beta-Asarone | 5281758 | |
| | | Croweacin | 5316141 | |
| | | Elemicin | 10248 | |
| | | (E)-Isocroweacin | 14601153 | |
| | | Methyl kakuol | 13672435 | |
| | | 3,4-Methylene-dioxypropiophenone | 95682 | |
| | | Asarinin | 11869417 | |
| | | Asatone | 431129 | |
| | | Tetradecane | 12389 | |
| | | 1-Tridecene | 17095 | |
| 2. | Scutellaria baicalensis | Acetophenone | 7410 | 4 |
| | | l-phenyl-1 | 11062753 | |

| | | | | |
|---|---|---|---|---|
| | (30) | (E)-4-phenyl-3-buten-2- one | 129825247 | |
| | | 3-butanedione | 6409633 | |
| | | palmitic acid | 985 | |
| | | oleic acid | 445639 | |
| | | b-carotene | 5280489 | |
| | | benzyl alcohol | 244 | |
| | | b-sitosterol | 222284 | |
| | | Benzoic acid | 243 | |
| | | Lutein | 5281243 | |
| | | Baicalin | 64982 | |
| | | Baicalein | 5281605 | |
| | | Wogonoside | 3084961 | |
| | | Wogonin | 5281703 | |
| | | Tenaxin-I | 159029 | |
| | | Viscidulin III | 5271991 | |
| | | Viscidulin I | 5320471 | |
| | | Viscidulin II | 5322059 | |
| | | Baicalein-7-O-D-glucoside | 5320313 | |
| | | Norwogonin | 5281674 | |
| | | Chrysin | 5281607 | |
| | | Chrysin-8-C-β-D-glucopyranoside | 44257620 | |
| | | Skullcapflavone II | 124211 | |
| | | Salvigenin | 161271 | |
| | | Oroxylin A | 5320315 | |
| | | Oroxylin A-7-O-glucuronide | 14655551 | |
| | | Eriodictyol | 440735 | |
| | | 5-Hydroxy-7,8-dimethoxyflavone | 188316 | |
| | | Dihydrobaicalin | 14135325 | |
| | | Dihydrooroxylin A | 5316733 | |
| | | Eriodictyol | 440735 | |
| 3. | Cinnamomum cassia (90) | endo-borneol | 1201518 | 5 |
| | | (−)-α-terpineol | 443162 | |
| | | β-bisabolene | 10104370 | |
| | | α-bisabolol | 1616126 | |
| | | curcumene | 92139 | |
| | | δ-cadinene | 12306054 | |
| | | espatulenol | 522266 | |
| | | caryophyllene oxide | 1742210 | |
| | | α-muurolene | 12306049 | |
| | | α-cadinol | 6431302 | |
| | | coniferaldehyde | 5280536 | |
| | | cinnamylalcohol | 5315892 | |
| | | 2-methoxycinnamaldehyde | 641298 | |
| | | 20 -methoxycinnamaldehyde | 641298 | |
| | | benzyl benzoate | 2345 | |
| | | 2-hydroxybenzaldehyde | 6998 | |
| | | 3-phenylpropanol | 31234 | |
| | | Benzaldehyde | 240 | |
| | | phenylethyl alcohol | 6054 | |
| | | benzenepropanal | 7707 | |

| | | | |
|---|---|---|---|
| | 1-terpineol | 11468 | 6 |
| | cis-β-terpineol | 8748 | 6 |
| | caryophyllene | 5281515 | 6 |
| | eugenol | 3314 | 6 |
| | cinnamyl acetate | 5282110 | 6 |
| | linalool | 6549 | 7 |
| | camphene | 6616 | 7 |
| | β-pinene | 440967 | 7 |
| | camphor | 2537 | 7 |
| | geranyl acetate | 1549026 | 7 |
| | copaene | 19725 | 7 |
| | 1-(1,5-dimethyl-4-hexenyl)-4-methylbenzene | 92139 | 7 |
| | Cedrene | 521207 | 7 |
| | α-calacorene | 528708 | 7 |
| | isoledene | 530426 | 7 |
| | cis-cinnamaldehyde | 6428995 | 7 |
| | trans-cinnamaldehyde | 637511 | 7 |
| | ethyl cinnamate | 637758 | 7 |
| | benzene,1,3-dimethyl | 7929 | 7 |
| | styrene | 7501 | 7 |
| | 1,3-pentanediol,2,2,4-trimethyl | 71330332 | 7 |
| | Decanal | 8175 | 7 |
| | dodecane, 2,6,10-trimethyl | 6430052 | 7 |
| | coumarin | 323 | 7 |
| | cinnzeylanol | 44559448 | 8 |
| | anhydrocinnzeylanol | 73099741 | 8 |
| | cinnzeylanone | 101693755 | 8 |
| | Anhydrocinnzeylanine | 131752069 | 8 |
| | cinncassiol A | 46173967 | 9 |
| | cinncassiol B | 71448932 | 9 |
| | cinncassiol C | 75144796 | 9 |
| | cinncassiol E | 139031772 | 9 |
| | cinnacasol | 102421920 | 9 |
| | perseanol | 21672106 | 10 |
| | cinncassiol D1 | 139031768 | 10 |
| | D1 glucoside | 131751606 | 10 |
| | D2 glucoside | 131751607 | 10 |
| | D4 glucoside | 131751659 | 10 |
| | trans-caryophyllene | 5281515 | 11 |
| | germacrene D | 5317570 | 11 |
| | cinnamaldehyde | 637511 | 11 |
| | cis-2-methoxycinnamic acid | 1622530 | 11 |
| | o-methoxycinnamaldehyde | 641298 | 11 |
| | 2,2,4,6,6-pentamethylheptane | 26058 | 11 |
| | 2,5,9-trimethyldecane | 522020 | 11 |
| | 2-ethyl-5-propylphenol | 522454 | 11 |
| | 3,4-dimethoxyphenethyl alcohol | 81911 | 11 |
| | 2,5-dimethylundecane | 28452 | 11 |
| | 2-hydroxycinnamic acid | 637540 | 12 |
| | 2-hydroxycinnamaldehyde | 5318169 | 12 |

| | | | | |
|---|---|---|---|---|
| | | 4-methoxycinnamaldehyde | 641294 | 12 |
| | | cinnamic acid | 444539 | 12 |
| | | cinnacasolide A | 70692598 | 13 |
| | | cinnacasolide B | 70692599 | 13 |
| | | cinnacasolide C | 70694646 | 13 |
| | | rosavin | 9823887 | 13 |
| | | dihydromelilotoside | 5316728 | 13 |
| | | methyl dihydromelilotoside | 46881368 | 13 |
| | | cinnacasside A | 25214691 | 14 |
| | | cinnacasside C | 25214689 | 14 |
| | | cinnacasside B | 102035893 | 15 |
| | | picrasmalignan A | 122395445 | 16 |
| | | (−)-syringaresinol | 11604108 | 16 |
| | | (+)-isolariciresinol | 160521 | 16 |
| | | (−)-secoisolariciresinol | 65373 | 16 |
| | | Lawsonicin | 70698092 | 16 |
| | | 5-methoxylariciresinol | 44559567 | 16 |
| | | (+)-5,5 -dimethoxylariciresinol | 14157882 | 16 |
| | | (1→6)-β-d-glucopyranoside | 476541 | 17 |
| | | (−)-lyoniresinol 3α-O-β-d-glucopyranoside | 10483388 | 17 |
| | | 5R-methyl-3-heptatriacontyl-2(5H)-furanone | 129882098 | 18 |
| | | Echinatin | 6442675 | |
| | | Glycyrin | 480787 | |
| | | Glycyrol | 5320083 | |
| | | Isoangustone A | 21591148 | |
| | | Isoglycyrol | 124050 | |
| | | 7-O-methylluteone | 441251 | |
| | | Glyasperin D | 480860 | |
| | | Dehydroglyasperin D | 10109594 | |
| | | Gancaonin I | 480777 | |
| | Glycyrrhiza uralensis | Semilicoisoflavone B | 5481948 | |
| 4. | | Kumatakenin | 5318869 | 19 |
| | (80) | Licoricone | 5319013 | |
| | | Angustone A | 15664151 | |
| | | Licoflavonol | 5481964 | |
| | | Topazolin | 5481965 | |
| | | Licoisoflavone A | 5281789 | |
| | | Wighteone | 5281814 | |
| | | Allolicoisoflavone B | 10383349 | |
| | | Lupiwighteone | 5317480 | |
| | | Licoarylcoumarin | 10090416 | |
| | | Isolicoflavonol | 5318585 | |
| | | Formononetin | 5280378 | |
| | | Genkwanin | 5281617 | |
| | | Glycycoumarin | 5317756 | |
| | | Isoglabrone | 102597283 | |
| | | Glyurallin A | 15818598 | 19 |
| | | 11b-hydroxy-11b,1-dihydromedicarpin | 44437741 | |
| | | Kaempferol 3-O-methyl ether | 5280862 | |
| | | Glycyrrhiza-isoflavone C | 10546844 | |

| | | | |
|---|---|---|---|
| | Isoliquiritigenin | 638278 | |
| | Isoglycycoumarin | 14187587 | |
| | Genistein | 5280961 | |
| | Kumatakenin B | 5318869 | |
| | Luteone | 5281797 | |
| | Licocoumarone | 503731 | |
| | Uralenol | 5315126 | |
| | Glicoricone | 10361658 | |
| | Pratensein | 5281803 | |
| | Dehydroglyasperin C | 480775 | |
| | Homobutein | 6438092 | |
| | Kaempferol | 5280863 | |
| | Gancaonin L | 14604077 | |
| | Licoisoflavanone | 392443 | 19 |
| | biochanin A | 5280373 | |
| | Liquiritigenin | 114829 | |
| | Licoflavone A | 5319000 | |
| | Glyasperin C | 480859 | |
| | Daidzein | 5281708 | |
| | 1-methoxyphaseollin | 10247433 | |
| | Isolupalbigenin | 26238934 | |
| | 6,8-diprenylgenistein | 480783 | |
| | 2'-hydroxyisolupalbigenin | 14237659 | |
| | Isoderrone | 14237660 | |
| | Licoricidin | 480865 | |
| | Glicophenone | 10021298 | |
| | Licoisoflavone B | 5481234 | |
| | Vicenin-2 | 442664 | |
| | Isoviolanthin | 101422758 | |
| | Liquiritin apioside | 10076238 | |
| | Liquiritin | 503737 | |
| | Ononin | 442813 | |
| | Isoliquiritin | 5318591 | |
| | Glycyroside | 101939210 | |
| | Sophoraflavone B | 5491513 | |
| | Daidzin | 107971 | |
| | Glycyrrhizic acid | 14982 | 20 |
| | lignoceric acid | 11197 | 20 |
| | methoxyficifolinol | 480872 | 20 |
| | docosanol | 12620 | 20 |
| | betulinic acid | 64971 | 20 |
| | docosyl caffeate | 5316952 | 20 |
| | neoglycyrol | 5320083 | 20 |
| | gancaonin H | 5481949 | 20 |
| | 3*R*-vestitol | 439310 | 20 |
| | Licoarylcoumarin | 10090416 | 20 |
| | 7,2',4'-trihydroxy-5-methoxy-3-arylcoumarin | 25015742 | 20 |
| | Isoliquiritigenin | 638278 | 20 |
| | Glycyrin | 480787 | 20 |
| | isoliquiritin apioside | 6442433 | 20 |

| | | | | |
|---|---|---|---|---|
| | | hedysarimcoumestan B | 11558452 | 20 |
| 5. | Astragalus membranaceus (30) | AH-1 | 132525571 | 21 |
| | | astragaloside I | 13996685 | |
| | | Astragaloside-II | 71306915 | |
| | | Astragaloside III | 441905 | |
| | | Astragaloside IV | 13943297 | |
| | | Astragaloside V | 71448939 | |
| | | Astragaloside VI | 71448940 | |
| | | Astragaloside VII | 14241100 | |
| | | Isoastragaloside I | 60148697 | |
| | | Isoastragaloside II | 60148655 | |
| | | Azukisaponin V methyl ester | 101683319 | |
| | | Malonylastragaloside | 50917280 | |
| | | astramembrannin II | 132492418 | |
| | | brachyoside B | 100927164 | |
| | | cyclocanthoside E | 21633193 | |
| | | astramembranoside A | 24795974 | |
| | | astramembranoside B | 24796110 | |
| | | Astragalan | 103026223 | |
| | | Kaempferol | 5280863 | |
| | | Isorhamnetin | 5281654 | |
| | | Rhamnocitrin | 5320946 | |
| | | kumatakenin | 5318869 | |
| | | rhamnocitrin-3-glucoside | 44259544 | |
| | | quercetin-3-glucoside | 5280804 | |
| | | Formononetin | 5280378 | |
| | | Calycosin | 5280448 | |
| | | 2′-hydroxy-3′,4′,7-trimethoxyisoflavone | 44257240 | |
| | | 3,9,10-trimethoxypterocarpan | 15689655 | |
| | | Astragalus polysaccharides (APS) | 2782115 | |
| 6. | Phellodendron amurense (70) | Berberine | 2353 | 22 |
| | | Berberrubine | 72703 | |
| | | Berberastine | 442180 | |
| | | Bis-[4-(dimethylamino)phenyl]methanone | 282874 | |
| | | Evodiamine | 442088 | |
| | | Palmatine | 19009 | |
| | | Tetrahydropalmatine | 5417 | |
| | | Tetrahydroberberine | 34458 | |
| | | Phellodendrine | 3081405 | |
| | | Magnocurarine | 53266 | |
| | | Magnoflorine | 73337 | |
| | | Υ-Fagarine | 107936 | |
| | | Canthin-6-one | 97176 | |
| | | 4-methoxy-N-methyl-2-quinolone | 182073 | |
| | | Oxypalmatine | 10926678 | |
| | | Candicine | 23135 | |
| | | Lotusine | 5274587 | |
| | | (−)-Oblongine | 157128 | |
| | | Tetrahydrojatrorrhizine | 185605 | 22 |
| | | Menisperine | 161487 | |

| | | |
|---|---|---|
| | (+) N-methylcorydine | 52949574 |
| | N-methylflindersine | 72819 |
| | Litcubine | 85243417 |
| | Tetrahydropalmatine | 5417 |
| | Xanthoplanine | 14262868 |
| | N-methylphoebine | 101731569 |
| | Columbamine | 72310 |
| | Epiberberine | 160876 |
| | Pteleine | 159650 |
| | Noroxyhydrastinine | 89047 |
| | Chilenine | 11025386 |
| | Rutecarpine | 65752 |
| | Skimmianine | 6760 |
| | Tembetarine | 167718 |
| | Tetramethyl-O-scutellarin | 96118 |
| | *Y*-hydroxybutenolide | 16221764 |
| | Armepavine | 442169 |
| | Demethyleneberberine | 363209 |
| | 8-oxoberberine | 12039004 |
| | 8-oxoepiberberine | 12799036 |
| | Oxyberberine | 11066 |
| | Oxypalmatine | 10926678 |
| | Kihadanin B | 156766 |
| | Niloticin | 14021529 |
| | Obaculactone or limonin | 179651 |
| | Obacunone or Obacunoic acid | 119041 |
| | Rutaevin | 441805 |
| | Coniferin | 5280372 |
| | Vanilloloside | 44577222 |
| | Herculin | 5318023 |
| | Ferulic acid | 445858 |
| | Quinic acid | 6508 |
| | Neochlorogenic acid | 5280633 |
| | 3-O-feruloylquinic acid | 9799386 |
| | Chlorogenic acid | 1794427 |
| | Methyl 3-*O*-feruloylquinate | 24813764 |
| | Methyl 5-O-feruloylquinate | 102004731 |
| | Sanleng acid | 5321100 |
| | (+/-)-lyoniresinol | 10483388 |
| | Amurensin | 5318156 |
| | Quercetin | 5280343 |
| | Phellamurin | 193876 |
| | Phellatin | 44258781 |
| | Phellavin | 5320517 |
| | Phellodendroside | 101316827 |
| | Icariside-1 | 5745470 |
| | Phellamuretin | 7092536 |
| | Isovaleric acid | 10430 |
| | 7-Dehydrostigmasterol | 12303924 |
| | Syringin | 5316860 |

| | | | | |
|---|---|---|---|---|
| | | Daucosterol | 5742590 | |
| | | α-Pinene | 6654 | |
| | | Sabinene | 18818 | |
| | | β-Myrcene | 31253 | |
| | | d-Limonene | 440917 | |
| | | (Z)-β-Ocimene | 5320250 | |
| | | (E)-β-Ocimene | 5281553 | |
| | Mentha arvensis | 3-Octanol | 11527 | |
| 7. | | l-Menthone | 26447 | 23 |
| | (15) | Isomenthone | 6986 | |
| | | Menthyl acetate | 27867 | |
| | | Neomenthol | 19243 | |
| | | Caryophyllene | 5281515 | |
| | | l-Menthol | 16666 | |
| | | α-Terpineol | 17100 | |
| | | Piperitone | 6987 | |
| | | 5-O-caffeoyl quinic acid | 5280633 | 24 |
| | | chlorogenic acid methyl ester | 6476139 | 24 |
| | | quercetin | 5280343 | 24 |
| | | quercitrin | 5280459 | 24 |
| | | isoquercitrin | 5280804 | 24 |
| | | rutin | 5280805 | 24 |
| | | saikosaponin D | 107793 | 24 |
| | Bupleurum falcatum | saikosaponin A | 167928 | 24 |
| 8. | | saikosaponin C | 131801344 | 24 |
| | (20) | 2,2,4-trimethyl-3-penten-1-ol | 79926 | 25 |
| | | 2,3-dimethyl-3-buten-2-ol | 82652 | 25 |
| | | 2,3-dimethyl-pentane | 11260 | 25 |
| | | 9-octadecenoic acid (z) | 6439696 | 25 |
| | | octadecanoic acid | 5281 | 25 |
| | | nonanedioic acid | 2266 | 25 |
| | | octanedioic acid | 10457 | 25 |
| | | 6-O-acetyl-saikosaponin d | 21637630 | 25 |
| | | Palbinone | 9841735 | |
| | | 13-Methyl tetradecanoic acid | 151014 | |
| | | Peonin (peonidin-3,5-diglucoside) | 44256843 | |
| | | Albiflorin | 24868421 | |
| | | Albiflorin R1 | 5317181 | |
| | | Benzoylpaeoniflorin | 21631106 | |
| | | 8-Debenzoylpaeoniflorin | 71452333 | |
| | Paeonia lactiflora | Paeonidanin B | 102417825 | |
| 9. | | Paeonidanin A | 44253993 | 26 |
| | (45) | Paeonidanin C | 46883513 | |
| | | Paeoniflorigenone | 70698143 | |
| | | Paeoniflorin | 442534 | |
| | | Paeonilactone A | 10081437 | |
| | | Paeonilactone B | 10375422 | |
| | | Paeonilactone C | 10471123 | |
| | | Pyrethrin I | 5281045 | |
| | | Pyrethrin II | 5281555 | |

| | | | | |
|---|---|---|---|---|
| | | β-Sitosterol | 521199 | |
| | | Casuariin | 14035442 | |
| | | Casuarictin | 73644 | |
| | | 5-Desgalloylstachyurin | 10417809 | |
| | | 1-O-galloyl-β-D-glucose | 124021 | |
| | | 3-O-Galloylquinic acid | 442988 | |
| | | 4-O-Galloylquinic acid | 475263 | |
| | | Pedunculagin | 442688 | |
| | | 1,2,3,4,6-Pentagalloylglucose | 65238 | |
| | | Strictinin | 73330 | |
| | | Tellimagrandin I | 442690 | |
| | | 1,3,6-Trigalloyl-β-D-glucose | 452707 | |
| | | 1,2,3-Tri-O-galloyl-β-D-glucose | 13270010 | |
| | | Benzoic acid | 243 | |
| | | Phenol (Carbolic acid) | 996 | |
| | | p-hydroxybenzoic acid | 135 | |
| | | gallic acid | 370 | |
| | | methylgallate | 7428 | |
| | | paeonol | 11092 | |
| | | paeonoside | 442924 | |
| | | (+)-catechin | 9064 | |
| | | Paeoniflorin | 442534 | 27 |
| | | Oxypaeoniflorin | 21631105 | |
| | | Benzoyloxypaeoniflorin | 21631107 | |
| | | Galloylpaeoniflorin | 46882879 | |
| | | betulinic acid | 64971 | |
| | | hederagenin | 73299 | |
| | | oleanolic acid | 10494 | |
| 10. | Atractylodes lancea (30) | Atractylenolide I | 5321018 | 28 |
| | | Atractylenolide II | 14448070 | 28 |
| | | Atractylenolide III | 155948 | 28 |
| | | Atractylone | 3080635 | 28 |
| | | Hinesol | 10878761 | 28 |
| | | β-Eudesmol | 91714393 | 28 |
| | | Atractylodin | 5321047 | 28 |
| | | Stigmasterol | 5280794 | 28 |
| | | coumarin osthol | 10228 | 29 |
| | | atractylochromene | 10244247 | 29 |
| | | Kudtdiol | 26471785 | 29 |
| | | atractyloside A | 71307451 | 29 |
| | | atractyloside B | 71448952 | 29 |
| | | pterocarpol | 12314741 | 29 |
| | | eudesma-4(14),7(11)-dien-8-one | 13986100 | 29 |
| | | (+) eudesma-4(14),7(11)-dien-8-one | 13986100 | 29 |
| | | atractylenolide IV | 132510447 | 29 |
| | | atractyloside C | 71448953 | 29 |
| | | atractyloside D | 71448954 | 29 |
| | | atractyloside E | 71448955 | 29 |
| | | atractyloside G | 71448957 | 29 |
| | | atractyloside I | 10929902 | 29 |

| | | | | |
|---|---|---|---|---|
| | | atractyloside F | 71448956 | 29 |
| | | heterolupeol | 604983 | 29 |
| | | Atractylodinol | 10012964 | 29 |
| | | Acetylatractylodinol | 5315531 | 29 |
| | | 1,3-di-O-caffeoylquinic acid | 6474640 | 29 |
| | | Osthol | 10228 | 29 |
| | | 5-hydroxymethyl furaldehyde | 237332 | 29 |
| | | L-phenylalanine | 6140 | 29 |
| | | diethyl phthalate | 6781 | 29 |
| 11. | Cimicifuga dahurica (25) | Geniposide | 107848 | 30 |
| | | 24-O-acetylhydroshengmanol | 157168 | 30 |
| | | prim-O-glucosylcimifugin | 14034912 | 30 |
| | | acerinol | 73347277 | 30 |
| | | cimigenol | 16020000 | 30 |
| | | Cimiracemoside C | 15541911 | 31 |
| | | Cimigenoside | 16088242 | 31 |
| | | 25-O-Acetylcimigenol | 46881255 | 31 |
| | | 25-Anhydrocimigenol | 15967434 | 31 |
| | | 23-epi-26-Deoxyactein | 21668683 | 31 |
| | | Actein | 10032468 | 31 |
| | | Cimicifugic acid A | 6449879 | 31 |
| | | Cimicifugic acid B | 6449880 | 31 |
| | | Cimicifugic acid D | 11742743 | 31 |
| | | Cimicifugamide | 5318530 | 31 |
| | | Visnagin | 6716 | 31 |
| | | 2-Isoferuloyl piscidic acid | 6450179 | 32 |
| | | Ferulic acid 4-*O*-*β*-D-glucopyranoside | 13916049 | 32 |
| | | Caffeic acid 3-*O*-*β*-D-glucopyranoside | 5281759 | 32 |
| | | Shomaside A | 46209783 | 32 |
| | | Caffeic ester glucoside | 5281761 | 32 |
| | | Caffeic methl ester | 689075 | 32 |
| | | Carboxymethyl isoferulate | 16729361 | 32 |
| | | 3, 4-Dimethoxycinnamic acid | 717531 | 32 |
| | | 2-Feruloyl piscidic acid | 10002902 | 32 |
| | | (*E*)-3-(3-Methyl-2-butenylidene)-2-indolinone | 5319526 | 32 |
| | | (Z)-3-(3-Methyl-2-butenylidene)-2-indolinone | 5249879 | 32 |
| 12. | Cnidium officinale (08) | 6-(1-oxopentyl)-salicylic acid methyl ester | 134612870 | 33 |
| | | Falcarindiol | 5281148 | 33 |
| | | conifenyl ferulate | 6441913 | 33 |
| | | ferulic aldehyde | 5280536 | 33 |
| | | senkyunolide C | 642374 | 33 |
| | | (E)-3-methoxy-1-(3-methoxyphenyl)propane | 15418925 | 33 |
| | | Coniferol | 1549095 | 33 |
| | | p-hydroxyphenol | 785 | 33 |
| 13. | Angelica dahurica (30) | Isoimperatorin | 68081 | 34 |
| | | imperatorin | 10212 | 34 |
| | | bergapten | 2355 | 34 |
| | | osthenol | 5320318 | 34 |
| | | xanthotoxin | 4114 | 34 |
| | | dehydrogeijerin | 620900 | 34 |

| | | | | |
|---|---|---|---|---|
| | | phellopterin | 98608 | 34 |
| | | 7-demethylsuberosin | 5316525 | 34 |
| | | Alloimperatorin | 69502 | 34 |
| | | Xanthotoxol | 65090 | 34 |
| | | Isooxypeucedanin | 625383 | 34 |
| | | Alloisoimperatorin | 5317436 | 34 |
| | | 5-hydroxy-8-methoxypsoralen | 5385192 | 34 |
| | | oxypeucedanin methanolate | 483514 | 34 |
| | | pabulenol | 3009225 | 34 |
| | | byakangelicin | 10211 | 34 |
| | | marmesin | 334704 | 34 |
| | | (+) -decursinol | 442127 | 34 |
| | | Heraclenol | 73253 | 34 |
| | | oxypeucedanin hydrate | 17536 | 34 |
| | | marmesinin | 216283 | 34 |
| | | ulopterol | 176475 | 34 |
| | | uracil | 1174 | 34 |
| | | oxypeucedanin | 160544 | 35 |
| | | byakangelicol | 3055167 | 35 |
| | | isopimpinellin | 68079 | 35 |
| | | pimpinellin | 4825 | 35 |
| | | scopoletin | 5280460 | 35 |
| 14. | Ephedra sinica (55) | 1-Phenylpropane-1,2-dione | 11363 | 36 |
| | | (S)-cathinone | 25203656 | 36 |
| | | (1R,2S)-norephedrine | 10297 | 36 |
| | | (2S,4S,5R)-2,4-dimethyl-5-phenyl oxazolidine | 130754154 | 36 |
| | | (1R,2S)-ephedrine | 6922965 | 36 |
| | | (1R,2S)-N-methylephedrine | 6918907 | 36 |
| | | (1S,2S)-norpseudoephedrine | 6918945 | 36 |
| | | (1S,2S)-pseudoephedrine | 7028 | 36 |
| | | (1S,2S)-N-methylpseudoephedrine | 7059595 | 36 |
| | | proanthocyanidin A4 | 13556387 | 36 |
| | | catechin | 9064 | 37 |
| | | epicatechin | 72276 | 37 |
| | | gallocatechin | 65084 | 37 |
| | | epigallocatechin | 72277 | 37 |
| | | Ephedradine A | 126326 | 37 |
| | | Ephedradine B | 156055 | 38 |
| | | Ephedradine C | 558490 | 38 |
| | | Ephedradine D | 100996000 | 38 |
| | | Feruloylhistamine | 10401784 | 38 |
| | | L(+)-Pseudoephedrine | 7028 | 38 |
| | | D(−)Norephedrine | 62943 | 38 |
| | | D(−)Methylephedrine | 3041454 | 38 |
| | | Ephedroxane | 161171 | 38 |
| | | 2, 3, 4-Trimethyl-5-phenyloxazolidine | 5322112 | 38 |
| | | Hordenine | 68313 | 38 |
| | | Maokonine | 54704413 | 38 |
| | | Tetramethylpyrazine | 14296 | 38 |
| | | N-methylbenzylamine | 7669 | 38 |

| | | | | |
|---|---|---|---|---|
| | | Herbacetin | 5280544 | 38 |
| | | Herbacetin 7-methyl ether | 44259958 | 38 |
| | | Pollenitin | 44259965 | 38 |
| | | Herbacetin 7-O-glucoside | 5318021 | |
| | | Kaempferol 3-O-rhamnoside 7-O-glucoside | 554033 | |
| | | Herbacetin 7-O-neohesperidoside | 102444811 | |
| | | Kaempferol-3-O-glucoside-7-O-rhamnoside | 14035324 | |
| | | Dihydroquercetin | 439533 | |
| | | Naringenin | 932 | |
| | | Hesperidin | 10621 | |
| | | (−)-epicatechin | 72276 | |
| | | (−)-epiafzelechin | 443639 | |
| | | Afzelechin | 442154 | |
| | | Leucocyanidin | 71629 | |
| | | Symplocoside | 44257110 | |
| | | Tricin | 5281702 | 38 |
| | | Apigenin | 5280443 | |
| | | 3-Methoxyherbacetin | 5319442 | |
| | | Swertisin | 124034 | |
| | | Isovitexin-2″-O-rhamnoside | 23844078 | |
| | | Leucodelphinidin | 3081374 | |
| | | Ephedrannin A | 21676348 | |
| | | Ephedrannin B | 25051177 | |
| | | Sesquipinsapol B | 101767126 | |
| | | Ethyl caprylate | 7799 | |
| | | Sitosterol | 222284 | |
| | | Trans-cinnamic acid | 444539 | |
| | | Physcion | 10639 | |
| | | Rhein | 10168 | |
| | | Panaxadione | 25233029 | |
| | | 20(S)-protopanaxatriol | 11468733 | |
| | | 3-keto-20(S)-protopanaxatriol | 44198762 | |
| | | ginsenoside Rg2 | 21599924 | |
| | | ginsenoside Rd | 24721561 | |
| | | phenethyl alcohol- xylopyranosyl(1→6)glucopyranoside | 131129 | |
| | | panaxoside A | 441923 | 39 |
| | | Panaxoside Re | 58774156 | 39 |
| | Panax ginseng (30) | protopanaxadiol | 9920281 | 39 |
| 15. | | panacon | 6325774 | 39 |
| | | panaxadiol | 73498 | 39 |
| | | panaxol | 328778 | |
| | | ginsenoside R0 | 11815492 | 39 |
| | | ginsenoside Rb-1 | 9898279 | 40 |
| | | ginsenoside Rb-2 | 6917976 | |
| | | ginsenoside Rc | 12855889 | |
| | | ginsenoside Rd | 24721561 | |
| | | ginsenoside Rf | 441922 | |
| | | ginsenoside Rg-1 | 441923 | |
| | | ginsenoside Rg-2 | 21599924 | |

| | | | | |
|---|---|---|---|---|
| | | ginsenoside Rg-3 | 9918693 | |
| | | ginsenoside Rh-1 | 12855920 | |
| | | ginsenoside Rh-2 | 119307 | |
| | | panacene | 181799 | |
| | | β-elemene | 6918391 | |
| | | citric acid | 311 | |
| | | fumaric acid | 444972 | |
| | | ketoglutaric acid | 51 | |
| | | choline | 305 | |
| 16. | Zingiber officinale (45) | [6]-gingerol | 442793 | 41 |
| | | [6]-shogaol | 5281794 | |
| | | [4]-gingerol | 5317596 | |
| | | [7]-gingerol | 11472344 | |
| | | [8]-gingerol | 168114 | |
| | | [10]-gingerol | 168115 | |
| | | [6]-Paradol | 94378 | |
| | | [7]- paradol | 13733135 | |
| | | [8]- paradol | 213821 | |
| | | [10]- paradol | 51352076 | |
| | | methyl [6]-paradol | 85807832 | |
| | | [4]- shogaol | 9794897 | |
| | | [8]- shogaol | 6442560 | |
| | | [10]- shogaol | 6442612 | |
| | | [12]-shogaol | 9975813 | |
| | | [6]-isoshogaol | 11694761 | |
| | | [6]- gingerdione | 162952 | |
| | | [8]- gingerdione | 14440537 | |
| | | [10]- gingerdione | 14440539 | |
| | | [12]- gingerdione | 86251913 | |
| | | citral/ geranial | 638011 | 42 |
| | | borneol | 64685 | |
| | | bisabolene | 3033866 | |
| | | Gingerdiol | 11369949 | |
| | | 1-dehydrogingerdione | 9796015 | |
| | | 5-acetoxy [6] gingerdiol | 101419545 | |
| | | Ar-curcumene | 92139 | |
| | | β-bisabolene | 10104370 | |
| | | (-) β-sesquiphellandrene | 519764 | |
| | | 6-methyl-5-hepten-2-one | 9862 | |
| | | α-phellandrene | 7460 | |
| | | β-phellandrene | 11142 | |
| | | limonene | 22311 | |
| | | citronellol | 8842 | |
| | | neral | 643779 | |
| | | geraniol | 637566 | |
| | | bornyl acetate | 6448 | |
| | | 2 undecanone | 8163 | |
| | | citronellyl acetate | 9017 | |
| | | α-copaene | 70678558 | |
| | | geranyl acetate | 1549026 | |

| | | | | |
|---|---|---|---|---|
| | | eucalyptol/ 1,8-cineole | 2758 | |
| | | isobornyl formate | 23623868 | 43 |
| | | $\alpha$-zingiberene | 521253 | 43 |
| | | $\alpha$ farnesene | 5281516 | 43 |
| 17. | Poria cocos (43) | dehydroeburicoic acid | 15250826 | 44 |
| | | 3-epi-dehydrotumulosic acid | 10005581 | 44 |
| | | dehydrotrametenolic acid | 15250826 | 44 |
| | | pachymic acid | 5484385 | 44 |
| | | eburicoic acid | 73402 | 44 |
| | | trametenolic acid | 12309443 | 44 |
| | | $\alpha$-amyrin acetate | 71597151 | 44 |
| | | adenosine | 60961 | 44 |
| | | 16$\alpha$-Hydroxytrametenolic acid | 132285301 | 45 |
| | | 3-O-Acetyl-16$\alpha$-hydroxytrametenolic acid | 15226712 | 45 |
| | | 3-O-Acetyl-16$\alpha$-hydroxydehydrotrametenolic acid | 15226714 | 45 |
| | | Dehydrotrametenonic acid | 44424826 | 45 |
| | | Dehydroeburiconic acid | 11431307 | 45 |
| | | Dehydropachymic acid | 15226717 | 45 |
| | | 3-epi-Dehydropachymic acid | 15226716 | 45 |
| | | Tumulosic acid | 12314446 | 45 |
| | | Dehydrotumulosic acid | 15225964 | 45 |
| | | 3-epi-Dehydrotumulosic acid | 10005581 | 45 |
| | | 15$\alpha$-Hydroxydehydrotumulosic acid | 16736459 | 45 |
| | | Polyporenic acid C | 9805290 | 45 |
| | | 6$\alpha$-Hydroxypolyporenic acid C | 101280198 | 45 |
| | | 29-Hydroxypolyporenic acid C | 139585448 | |
| | | 25-Hydroxy-3-epi-tumulosic acid | 46882675 | |
| | | 5$\alpha$-8$\alpha$-Peroxydehydrotumulosic acid | 16736654 | |
| | | Poricoic acid B | 5471852 | |
| | | 16-Deoxyporicoic acid B | 16736458 | |
| | | Poricoic acid E | 15225966 | |
| | | Poricoic acid BM | 15225967 | |
| | | Poricoic acid G | 5471966 | |
| | | Poricoic acid GM | 44556812 | |
| | | Poricoic acid A | 5471851 | |
| | | Poricoic acid C | 56668247 | |
| | | Poricoic acid D | 44424827 | |
| | | Poricoic acid F | 101928114 | |
| | | Poricoic acid H | 10918099 | |
| | | Poricoic acid CM | 16736060 | |
| | | Poricoic acid DM | 44424830 | |
| | | Poricoic acid HM | 44556877 | |
| | | 6,7-Dehydroporicoic acid H | 44556878 | |
| | | 25-Hydroxyporicoic acid C | 44556811 | |
| | | 25-Hydroxyporicoic acid H | 16736865 | |
| | | 26-Hydroxyporicoic acid DM | 44556810 | |
| | | 25-Methoxyporicoic acid A | 46882628 | |
| 18. | Paeonia suffruticosa | Mudanpioside F | 21631108 | 46 |
| | | Procyanidin B6 | 474540 | |

| | | | |
|---|---|---|---|
| | Oxypaeoniflorin | 21631105 | |
| (60) | Procyanidin B6 | 474540 | |
| | Mudanpioside E | 86278277 | |
| | (þ)-Catechin | 107957 | |
| | Apiopaeonoside | 127509 | |
| | Methyl gallate | 7428 | |
| | Paeonolide | 442923 | |
| | 1,3,6-Tri-O-galloyl-b-D-glucose | 452707 | |
| | Suffruticoside A | 9986231 | |
| | Suffruticoside B | 10258205 | |
| | Suffruticoside C | 10258206 | |
| | Suffruticoside D | 5321547 | |
| | Tetragalloyl glucose | 102012877 | |
| | Suffruticoside A | 9986231 | |
| | Suffruticoside B | 10258205 | |
| | Suffruticoside C | 10258206 | |
| | Suffruticoside D | 5321547 | |
| | Quercetin 7-O-glucoside | 5381351 | |
| | Galloylpaeoniflorin | 46882879 | |
| | Albiflorin | 24868421 | |
| | 1,2,3,4,6-Penta-O-galloyol-beta-D glucose | 65238 | |
| | Mudanpioside H | 71457654 | |
| | Hexagalloyl glucose | 54036807 | |
| | Galloyloxypaeoniflorin | 3036133 | |
| | Benzoyloxypaeoniflorin | 21631107 | |
| | Quercetin 3-O-rhamnosylglucoside | 5491657 | |
| | Mudanpioside C | 21631098 | |
| | Benzoylpaeoniflorin | 21631106 | |
| | Mudanpioside B | 21631102 | |
| | hederagenin | 73299 | 47 |
| | chrysoeriol | 5280666 | 47 |
| | trans-ε-viniferin | 5281728 | 47 |
| | trans-resveratrol | 445154 | 47 |
| | stigmasterol | 5280794 | 47 |
| | β-daucosterol | 5742590 | 47 |
| | quinic acid | 6508 | 48 |
| | galloylquinic acid | 129650210 | 48 |
| | kaempferol 3,7-di-O-glucoside | 6325460 | 48 |
| | isorhamnetin 3,7-di-O-glucoside | 5323537 | 48 |
| | galloyl glucose | 124021 | 48 |
| | pentagalloylglucose | 65238 | 48 |
| | hexagalloylglucose | 129630523 | 48 |
| | heptagalloylglucose | 25202629 | 48 |
| | Paeonisuffrone | 10104180 | 49 |
| | Paeonisuffral | 101050848 | 49 |
| | Deoxypaeonisuffrone | 10035257 | 49 |
| | mudanpioside A | 21631101 | 49 |
| | mudanpioside D | 21631103 | 49 |
| | α-benzoyloxypaeoniflorin | 102597515 | 49 |
| | β-benzoyloxypaeoniflorin | 21631107 | 49 |

| | | | | |
|---|---|---|---|---|
| | | oxypaeonidanin | 71457653 | 49 |
| | | 9-epi-oxypaeonidanin | 71450490 | 49 |
| | | trigalloyl-glucose | 440308 | 49 |
| | | (−)-Epigallocatechin gallate | 65064 | 49 |
| | | uridine | 6029 | 49 |
| | | thymidine | 5789 | 49 |
| | | paeoniflorigenone | 70698143 | 49 |
| 19. | Aconitum carmichaelii Debeaux (65) | Honokiol | 72303 | 50 50 50 50 50 51 |
| | | pinoresinol | 73399 | |
| | | salicylic acid | 338 | |
| | | p-hydroxy-cinnamic acid | 637542 | |
| | | songorine | 71456946 | |
| | | karakoline | 441742 | |
| | | Aconitine | 245005 | |
| | | Mesaconitine | 441747 | |
| | | Hypaconitine | 441737 | |
| | | Talatizamine | 441761 | |
| | | Isotalatizidine | 3084020 | |
| | | Neoline | 120682 | |
| | | Fuziline | 14163819 | |
| | | Isodelphinine | 102146471 | |
| | | Benzoylmesaconine | 24832659 | |
| | | Senbusine A | 158048 | |
| | | Senbusine C | 14163819 | |
| | | Hokbusine A | 24832661 | |
| | | Benzoylaconine | 20055771 | |
| | | Benzoylhypaconine | 78358526 | |
| | | Neojiangyouaconitine | 78358553 | |
| | | Aldohypaconitine | 5317220 | |
| | | Deoxyaconitine | 21598997 | |
| | | Beiwutine | 21627927 | |
| | | Aconifine | 441705 | |
| | | Aconine | 20054813 | |
| | | Yunaconitine | 155569 | |
| | | Chasmanine | 20055812 | |
| | | Foresticine | 154723942 | |
| | | N-deethylaconine | 101552717 | |
| | | Beiwutinine | 101552718 | |
| | | Hypaconine | 101671038 | |
| | | Mesaconine | 76189547 | |
| | | Oxonitine | 6708531 | 51 |
| | | Guiwuline | 70688216 | |
| | | Bullatine B | 120682 | |
| | | Penduline | 179472 | |
| | | Ignavine | 71448929 | |
| | | Delgradine | 139075130 | |
| | | 14-O-Anisoylneoline | 12068477 | |
| | | 14-O-Acetyneoline | 14312993 | |
| | | Foresaconitine | 20055981 | |
| | | Crassicauline A | 157539 | |

| | | | | |
|---|---|---|---|---|
| | | Hetisine | 431673 | |
| | | Songoramine | 14526618 | |
| | | 12-Epinapelline | 3133561 | |
| | | Aconicarchamine A | 102484827 | |
| | | Aconicarchamine B | 102484828 | |
| | | Napelline | 441749 | |
| | | Carmichaeline A | 102144179 | |
| | | Yokonoside | 3085046 | |
| | | Higenamine | 114840 | |
| | | Salsolinol | 91588 | |
| | | Fuzitine | 72201752 | |
| | | Oleracein E | 21574476 | |
| | | 16beta-Hydroxycardiopetaline | 102511299 | |
| | | Columbianine | 101457306 | |
| | | Aconitamide | 15944660 | |
| | | 6″-O-Acetylliquiritin | 101051311 | |
| | | Liquiritigenin | 114829 | |
| | | Isoliquiritigenin | 638278 | |
| | | Liquiritin | 503737 | |
| | | Gracillin | 159861 | |
| | | Glyceryl monopalmitate | 3084463 | |
| | | Fuzinoside | 102339571 | |
| 20. | Salvia miltiorrhiza (60) | Danshensu | 11600642 | 52 |
| | | protocatechuic aldehyde | 8768 | |
| | | lithospermic acid | 6441498 | |
| | | tanshinone IIB | 9926694 | |
| | | dihydrotanshinone I | 11425923 | |
| | | tanshinone I | 114917 | |
| | | miltiradiene | 20837867 | |
| | | isoquercitrin | 5280804 | |
| | | salvianolic acid A | 5281793 | 53 |
| | | salvianolic acid B | 11629084 | |
| | | salvianolic acid C | 13991590 | |
| | | salvianolic acid D | 75412558 | |
| | | salvianolic acid E | 86278266 | |
| | | salvianolic acid G | 11530200 | |
| | | methyl rosmarinate | 6479915 | |
| | | dimethyl lithospermate | 70688393 | |
| | | lithospermic acid B | 6451084 | |
| | | ursolic acid | 64945 | |
| | | tigogenin | 99516 | |
| | | tanshinone IIA | 164676 | |
| | | tanshinone VI | 149138 | |
| | | cryptotanshinone | 160254 | |
| | | isotanshinone I | 623940 | |
| | | isotanshinone II | 44425166 | |
| | | isotanshinone IIB | 184102 | |
| | | isocryptotanshinone | 626608 | |
| | | hydroxytanshinone IIA | 5318349 | |
| | | methyl tanshinonate | 14610613 | |

| | | | | |
|---|---|---|---|---|
| | | danshenxinkun A | 149138 | |
| | | danshenxinkun B | 5320113 | |
| | | danshenxinkun C | 5320114 | |
| | | danshenxinkun D | 127172 | |
| | | dihydroisotanshinone I | 89406 | |
| | | neocryptotanshinone | 389888 | |
| | | deoxyneocryptotanshinone | 15690458 | |
| | | salviol | 13966146 | 53 |
| | | nortanshinone | 10062187 | |
| | | Tanshindiol A | 16730071 | |
| | | Tanshindiol B | 5321620 | |
| | | Tanshindiol C | 126072 | |
| | | Miltirone | 160142 | |
| | | 1,2-Dihydrotanshinqiunone | 105119 | |
| | | Ferruginol | 442027 | |
| | | 4-methylenemiltirone | 14609851 | |
| | | Tanshinlactone | 5321617 | |
| | | danshinspiroketallactone | 5316298 | |
| | | Epidanshenspiroketallactone | 102004791 | |
| | | cryptoacetalide | 46896125 | |
| | | miltiodiol | 11011966 | |
| | | miltipolone | 10086184 | |
| | | norsalvioxide | 14139391 | |
| | | 2-isopropyl-8-methylphenanthrene-3,4-dione | 135872 | |
| | | Dihydrotanshinone | 5316743 | 54 |
| | | Neosalvianen | 11472648 | |
| | | Salvianen | 11186248 | |
| | | Salviadione | 135442608 | |
| | | Lithospermic acid B | 6451084 | |
| | | Dihydrotanshinone I | 11425923 | |
| | | Cryptotanshinone | 160254 | |
| | | Tanshinone IIA | 164676 | |
| 21. | Citrus unshiu (25) | Hesperidin | 10621 | 55 |
| | | Naringin | 442428 | 55 |
| | | Neohesperidin | 442439 | 55 |
| | | Naringenin | 932 | 55 |
| | | Hesperetin | 72281 | 55 |
| | | *p*-coumaric acid | 637542 | 56 |
| | | sinapic acid | 637775 | 56 |
| | | protocatechuic acid | 72 | 56 |
| | | *p*-hydroxybenzoic acid | 135 | 56 |
| | | vanillic acid | 8468 | 56 |
| | | narirutin | 442431 | 56 |
| | | Nobiletin (3′,4′,5,6,7,8-hexamethoxyflavone) | 72344 | 57 |
| | | kaempferol 3-O-rutinoside | 5318767 | 57 |
| | | limocitrin 3-glucoside | 44259988 | 57 |
| | | didymin (4′-methoxyl naringenin 7- O-rutinoside) | 16760075 | 57 |
| | | naringenin 7-Orutinoside | | |
| | | Hesperetin-7-O-glucoside | 85704 | 57 |
| | | Prunin | 20111686 | 58 |

| | | | | |
|---|---|---|---|---|
| | | Sinensetin | 92794 | 58 |
| | | Tangeretin | 145659 | 58 |
| | | γ-terpinene | 68077 | 58 |
| | | 2-β-pinene | 7461 | 59 |
| | | 1-methyl-2-isopropylbenzene | 6654 | 59 |
| | | L-limonene | 10703 | 59 |
| | | β-ocimene | 439250 | 59 |
| | | | 5281553 | 59 |
| 22. | Gardenia jasminoides Ellis (75) | geniposidic acid | 443354 | 60 |
| | | chlorogenic acid | 1794427 | |
| | | genipin-1-β-gentiobioside | 14179128 | |
| | | geniposide | 107848 | |
| | | genipin | 442424 | |
| | | crocin-1 | 5281233 | |
| | | crocin-2 | 9940690 | |
| | | jasminoside I | 102596097 | |
| | | gardenoside | 24721095 | |
| | | gardaloside | 11631807 | |
| | | 5, 7, 3′, 4′, 5′-pentamethoxyflavone | 16044716 | |
| | | shikimic acid | 8742 | |
| | | 1, 2, 4-benzenetriol | 10787 | |
| | | 3, 4-dimethoxy-benzoic acid | 86584921 | |
| | | dibutyl phthalate | 3026 | |
| | | diisobutyl phthalate | 6782 | |
| | | α-gardiol | 101936008 | |
| | | β-gardiol | 57340454 | |
| | | genameside C | 11692460 | |
| | | deacetylasperulosidic acid | 12315350 | |
| | | syringic acid | 10742 | |
| | | syringaldehyde | 8655 | |
| | | vanillic acid | 8468 | |
| | | 3-hydroxy-vanillic acid | 129848169 | |
| | | 3, 4, 5-trimethoxy-phenol | 69505 | |
| | | 4-methoxy-benzaldehyde | 31244 | |
| | | 7-hydroxy-5-methoxy-chromone | 129848159 | |
| | | 5, 7, 3′-trihydroxy-6, 4′, 5′-trimethoxyflavone | 5496475 | |
| | | 3beta,23- dihydroxyurs-12-en-28-oic acid | 14136881 | |
| | | emodin | 3220 | |
| | | physcion | 10639 | |
| | | stearic acid | 5281 | |
| | | Gardenal | 4763 | |
| | | Geniposide | 107848 | |
| | | 6-α-hydroxy geniposide | 6325021 | 60 |
| | | Feretoside | 442433 | |
| | | genipin-1-β-gentiobioside | 14179128 | |
| | | shanzhiside | 11948668 | |
| | | lamalbidic acid | 101434718 | |
| | | picrocrocinic acid | 5320582 | |
| | | jasminoside A | 98050833 | |

| | | | | |
|---|---|---|---|---|
| | | epijasminoside A | 76551288 | |
| | | jasminoside R | 71552546 | |
| | | jasminoside S | 71552547 | |
| | | jasminoside T | 71552548 | |
| | | chikusetsusaponin IVa methyl ester | 637855 | 60 |
| | | chikusetsusaponin IVa butyl ester | 44566502 | |
| | | protocatechuic acid | 72 | |
| | | genipin-gentiobioside | 3082301 | |
| | | 7alpha-hydroxy sitosterol | 146158661 | |
| | | jasminosides R | 71552546 | |
| | | z-3-hexenyl tiglate | 5352469 | |
| | | trans-beta-ocimene | 5281553 | |
| | | genipin 1-O-beta-D-gentiobioside | 3082301 | |
| | | scandoside methyl ester | 442433 | |
| | | deacetylasperulosidic acid methyl ester | 6325021 | |
| | | 10-O-succinoylgeniposide | 44255239 | |
| | | 6′-O-acetylgeniposide | 44253991 | |
| | | 10-O-acetylgeniposide | 6324916 | |
| | | Jasminodiol | 24896698 | |
| | | jasminoside H | 102596096 | |
| | | jasminoside I | 102596097 | |
| | | imperatorin | 10212 | |
| | | isoimperatorin | 68081 | |
| | | crocetin | 5281232 | |
| | | sudan III | 62331 | |
| | | crocin-3 | 10461942 | |
| | | methyl 5-O-caffeoyl-3-O-sinapoylquinate | 11671431 | 60 |
| | | ethyl 5-O-caffeoyl-3-O-sinapoylquinate | 11699888 | |
| | | methyl 5-O-caffeoyl-4- O-sinapoylquinate | 11635556 | |
| | | ethyl 5-O-caffeoyl-4-O-sinapoylquinate | 11512664 | |
| | | methyl 3,5-di-O-caffeoyl-4-O-(3- hydroxy-3-methyl) glutaroylquinate | 11693219 | |
| | | Ixoroside | 44566558 | |
| | | 8-epiapodantheroside | 11372643 | |
| | | gardenate A | 10611205 | |
| | | 2-hydroxyethylgardenamide A | 10825707 | |
| | | Jasminoside F | 10807517 | |
| 23. | Gentiana scabra Bunge (15) | β-amyrin | 225687 | 61 |
| | | β-amyrin acetate | 345510 | 61 |
| | | uvaol | 92802 | 61 |
| | | gentiopicroside | 88708 | 62 |
| | | 6beta-hydroxyswertiajaposide A | 11153922 | 62 |
| | | Gelidoside | 46174003 | 62 |
| | | Trifloroside | 101688128 | 62 |
| | | Scabraside | 159134 | 62 |
| | | Durvillonol | 634965 | 63 |
| | | masilinic acid | 73659 | 63 |
| | | urjinolic acid | 146156259 | 63 |
| | | 3beta-erythrodiol | 101761 | 63 |
| | | corosolic acid | 6918774 | 63 |

| | | | | |
|---|---|---|---|---|
| | | pygenic acid C | 69049736 | 63 |
| | | chiratenol | 14831162 | 63 |
| 24. | Magnoliae Officinalis Cortex (105) | Magnolol | 72300 | 64 |
| | | Honokiol | 72303 | |
| | | O-methylhonokiol | 155160 | |
| | | Piperitylmagnolol | 11732171 | |
| | | Dipiperitylmagnolol | 101612410 | |
| | | Piperitylhonokiol | 13337242 | |
| | | Bornylmagnolol | 13337239 | |
| | | Eudesmagnolol | 14587417 | |
| | | Eudeshonokiol A | 14587421 | |
| | | Eudeshonokiol B | 21726646 | |
| | | Clovanemagnolol | 10323149 | |
| | | Caryolanemagnolol | 21726647 | |
| | | Magnolignan A | 5319201 | |
| | | Magnolignan B | 5319202 | |
| | | Magnolignan C | 5319203 | |
| | | Magnolignan D | 5319204 | |
| | | Magnolignan A−2-O-beta-D-glucopyranoside | 71720154 | |
| | | Randainal/Magnaldehyde B | 5320888 | |
| | | Randaiol | 13337243 | |
| | | Magnatriol B | 5319191 | |
| | | Magnaldehyde D | 5319189 | |
| | | Magnaldehyde E | 5319190 | 64 |
| | | 4′-methoxymagnaldehyde B | 23657448 | |
| | | 4′-methoxymagnaldehyde E | 23657447 | |
| | | Magnolignan E | 15714551 | |
| | | Coumanolignan | 24796112 | |
| | | Magnaldehyde C | 5319188 | |
| | | 4′-methoxymagndialdehyde | 23634517 | |
| | | Manglieside D | 102473624 | |
| | | Magnolignan F | 5319207 | |
| | | Magnolignan I | 373768 | |
| | | Isomagnolol | 159137 | |
| | | Obovatol | 100771 | |
| | | Eudesobovatol A | 442837 | |
| | | Eudesobovatol B | 10346031 | |
| | | Obovatal | 6439677 | |
| | | Magnolignan G | 15714552 | 64 |
| | | Magnolignan H | 373767 | |
| | | Icariside E5 | 91884923 | |
| | | Lariciresinol | 332427 | |
| | | (+)-Syringaresinol | 443023 | |
| | | Pinoresinol−4-O-beta-D-glucopyranoside | 486614 | |
| | | Magnoloside A | 21629882 | |
| | | Magnoloside B | 14018784 | |
| | | Magnoloside C | 14018786 | |
| | | Magnoloside D | 131676053 | |
| | | Crassifolioside | 102193658 | |
| | | Acteoside | 5281800 | |

| | | | |
|---|---|---|---|
| | Tachioside | 11962143 | |
| | Syringaldehyde | 8655 | |
| | Sinapic aldehyde | 5280802 | |
| | Isosyringinoside | 57399043 | |
| | Erigeside C | 14132346 | |
| | Coniferaldehyde | 5280536 | |
| | Coniferyl alcohol | 1549095 | |
| | p-Coumaric acid | 637542 | |
| | Caffeic acid methyl ester | 689075 | |
| | Coniferin | 5280372 | |
| | Magnolianone | 16739270 | |
| | O-methyleugenol | 7127 | |
| | Chavicol | 68148 | |
| | p-Hydroxybenzaldehyde | 126 | 64 |
| | Loliolide | 100332 | |
| | Blumenol A | 5280462 | |
| | Blumenol B | 14135402 | |
| | S-(+)-dehydrovomifoliol | 688492 | |
| | Grasshopper ketone | 13922639 | |
| | (S)-tembetarine | 167718 | |
| | Reticuline | 439653 | |
| | Magnoflorine | 73337 | |
| | (+)-Laurifoline | 12305611 | |
| | (+)-Menisperine | 30358 | |
| | (+)-Xanthoplanine | 5315336 | |
| | Asimilobine | 160875 | |
| | Lysicamine | 122691 | |
| | Roemerine | 119204 | |
| | Anonaine | 160597 | |
| | Anolobine | 164710 | |
| | Liriodenine | 10144 | |
| | Nornantenine | 3084228 | |
| | Anaxagoreine | 13891860 | |
| | N-nornuciferine | 12313579 | |
| | 10-demethylcryptaustoline | 100969440 | |
| | N-methylisosalsoline | 40091 | 64 |
| | N-Feruloylputrescine(trans) | 92339985 | |
| | Indole–3-aldehyde | 10256 | |
| | Betaine | 247 | |
| | Limonene | 22311 | |
| | Caryophyllene | 5281515 | |
| | Caryophyllene epoxide | 14350 | |
| | β- eudesmol | 91457 | |
| | γ- eudesmol | 6432005 | |
| | Cryptomeridiol | 165258 | |
| | β-sitostenone | 60123241 | |
| | Stigmasta– 4,22-dien– 3-one | 6442194 | |
| | Hyperoside | 5281643 | |
| | Afzelin | 5316673 | |
| | Choerospondin | 157745 | |

| | | | |
|---|---|---|---|
| | Isorhamnetin− 3-O-β-D-glucoside | 5318645 | |
| | Choerospondin | 157745 | |
| | Ethyl palmitate (Ethyl hexadecanoate) | 12366 | |
| | Ethyl stearate (Ethyl octadecanoate) | 8122 | |
| | Arachidic acid (Icosanoic acid) | 10467 | |
| | 1-hexacosanol (hexacosan−1-ol) | 68171 | |
| | Palmitone (hentriacontan−16-one) | 94741 | |
| | Nonacosylic acid (Nonacosanoic acid) | 20245 | |
| 25. Ophiopogon japonicus (30) | glycoside C<br>nolinospiroside F<br>ophiopogonin B<br>ophiopogonin D<br>ophiopogonin A<br>diosgenin<br>ophiopogonin C′<br>ophiopogonin D′<br>ophiopogonin P<br>ophiopogonin Q<br>sprengerinin A<br>cixiophiopogon A<br>ophiopojaponin C<br>ophiopogonin R<br>ophiopogonin S<br>prazerigenin A | 44566500<br>86289919<br>46173857<br>46173859<br>46173858<br>99474<br>4483248<br>10033524<br>102195415<br>71522132<br>102081298<br>102004869<br>146160153<br>71523896<br>71523897<br>13833781 | 65 |
| | methylophiopogonone B | 23259413 | 65 |
| | ophiopogonone B | 14826840 | 65 |
| | methylophiopogonone A | 10065830 | 65 |
| | 6-aldehydo-isoophiopogonone A | 5317207 | 65 |
| | ophiopogonone A | 10087732 | 65 |
| | ophiopogonone C | 11142766 | 65 |
| | ophiopogonanone E | 5316797 | 65 |
| | methylophiopogonanone B | 46886723 | 65 |
| | methylophiopogonanone A | 5319741 | 65 |
| | ophiopogonanone A | 9996586 | 65 |
| | ophiopogonanone C | 10871974 | 65 |
| | Cryptomeridiol | 165258 | 66 |
| | Pennogenin | 12314056 | 66 |
| | Sprengerinin C | 44583957 | 66 |
| 26. Perilla frutescens L. (200) | 3-Epicorosolic acid<br>3-Epimaslinic acid<br>Augustic acid<br>Corosolic acid<br>Hyptadienic acid<br>Pomolic acid<br>Tormentic acid<br>Triacylglycerol<br>Pentadecanoic acid | 15917998<br>25564831<br>15560128<br>6918774<br>14605533<br>382831<br>73193<br>11146<br>13849 | 67 |
| | Stearic acid | 5281 | |
| | β-Cholestanol | 6665 | |

| | | |
|---|---|---|
| | Docosanol | 12620 |
| | Eicosanol | 12404 |
| | Heneicosanol | 85014 |
| | Heptacosanol | 74822 |
| | Hexacosanol | 68171 |
| | Octacosanol | 68406 |
| | Tetracosanol | 10472 |
| | Triacontanol | 68972 |
| | Tricosanol | 18431 |
| | β-Tocopherol | 6857447 |
| | γ-Tocopherol | 92729 |
| | δ-Tocopherol | 92094 |
| | Rosmarinic acid methyl ester | 3012090 |
| | Apigenin 7-O-glucuronide | 5319484 |
| | Apigenin 7-O-diglucuronide | 126843388 |
| | Cimidahurinine | 5315870 |
| | Chrysoeriol | 5280666 |
| | Luteolin 7-O-diglucuronide | 146036993 |
| | Luteolin 7-O-glucoside | 5280637 |
| | Luteolin 7-O-glucuronide | 5280601 |
| | Luteolin-5-O-glucoside | 5317471 |
| | Scutellarein | 5281697 |
| | Chrysontenin | 197081 |
| | Cyanin | 441688 |
| | Malonylshisonin | 11972402 |
| | Shisonin | 5282068 |
| | (E,E)-α-Farnesene | 5281516 |
| | (Z)-3-Hexenyl acetate | 5363388 |
| | (Z,E)-α-Farnesene | 5362889 |
| | 1,10-Decanediol | 37153 |
| | 1,2-Benzenedicarboxylic acid | 1017 |
| | 1,6-Cyclodecadiene | 5365639 |
| | 10-Undecyn-1-ol | 76015 |
| | 1-Cyclohexene-1-methanol | 317542 |
| | 1-Octen-3-ol | 18827 |
| | 2,2-Dimethylpentane | 11542 |
| | 2,4,6-Triisopropylphenol | 82158 |
| | 2,4-Hexadienal | 637564 |
| | 2-Acetyl-5-methyl furan | 14514 |
| | 2-Acetylfuran | 14505 |
| | 2-Butylamine | 24874 |
| | 2-Cyclopentenone | 13588 |
| | 2-Ethyladamantane | 139758 |
| | 2-Hexanoylfuran | 61738 |
| | 2-Hexenal | 5281168 |
| | 2-Hydroxypyridine | 8871 |
| | 2-Isopropylidene-3-methylhexa-3,5-dienal | 5368460 |
| | 2-Methoxy-3-propenyl-phenol | 74069607 |
| | 2-Methyl-2-cyclopentenone | 14266 |
| | 2-Methylcyclopentanone | 14265 |

| | | |
|---|---|---|
| | 2-Nonyne | 140536 |
| | 3,5-Diethyl-toluene | 16302 |
| | 3-Octanol | 11527 |
| | 4,4-Dimethyl-2-cyclopenten-1-one | 140955 |
| | 4-Tert-pentylphenol | 6643 |
| | Acetophenone | 7410 |
| | Acetyl eugenol | 7136 |
| | a-Cubebene | 86609 |
| | Alloaromadendrene | 91354 |
| | All-trans-squalene | 638072 |
| | Anisole | 7519 |
| | Apiol | 10659 |
| | Asarone | 636822 |
| | a-Terpinyl acetate | 111037 |
| | Benzene acetaldehyde | 998 |
| | Bornyl acetate | 6448 |
| | Cadina-3,9-diene | 10657 |
| | Calarene | 28481 |
| | Camphane | 92108 |
| | Carvone | 7439 |
| | Caryophyllene | 5281515 |
| | Caryophyllene oxide | 1742210 |
| | cis-Asarone | 5281758 |
| | cis-Geraniol | 643820 |
| | cis-Lanceol | 6536796 |
| | cis-Nerolidol | 5320128 |
| | cis-Ocimene | 5320250 |
| | cis-Verbenol | 164888 |
| | Cosmene | 5368451 |
| | Cuminaldehyde | 326 |
| | Curlone | 196216 |
| | Cycloheptane | 9265 |
| | Cyclohexanone | 7967 |
| | Decane | 15600 |
| | Dihydrocarveol | 12072 |
| | Dihydrocarveol acetate | 30248 |
| | Dodecane | 8182 |
| | Egomaketone | 42978 |
| | Elemicin | 10248 |
| | Elixene | 94254 |
| | Elsholtziaketone | 521240 |
| | Eremophilene | 12309744 |
| | Eucalyptol | 2758 |
| | Farnesol | 445070 |
| | Furfuryl alcohol | 7361 |
| | Geraniol | 637566 |
| | Germacrene D | 5317570 |
| | Germacrene D-4-ol | 5352847 |
| | Heneicosane | 12403 |
| | Hexadecane | 11006 |

| | |
|---|---|
| Hexahydrofarnesyl acetone | 10408 |
| Humulene epoxide II | 10704181 |
| Isobornyl acetate | 247573 |
| Isocaryophyllene | 5281522 |
| Isoegomaketone | 5318556 |
| Isoelemicin | 5318557 |
| Isoeugenol | 853433 |
| Isolimonene | 521268 |
| Isomenthone | 6986 |
| Isopulegone | 34645 |
| Limonene oxide | 91496 |
| Limonene oxide, trans | 8029780 |
| Linalool oxide trans | 6432254 |
| Linalyl oxide cis | 6428573 |
| Longifolene | 289151 |
| Longipinocarvone | 535296 |
| Massoia lactone | 39914 |
| Menthol | 1254 |
| Menthone | 26447 |
| Methyl chavicol | 8815 |
| Methyl eugenol | 7127 |
| Methyl geranate | 5365910 |
| Methyl isoeugenol | 7128 |
| Methyl thymyl ether | 14104 |
| M-Mentha-6,8-diene | 102625 |
| Naginata ketone | 564412 |
| Nerol acetate | 1549025 |
| n-Heptadecane | 12398 |
| Nonacosane | 12409 |
| Nonane | 8141 |
| n-Tricosane | 12534 |
| Octacosane | 12408 |
| Patchoulane | 29408 |
| p-Cymene | 7463 |
| Pentacosane | 12406 |
| Perilla ketone | 68381 |
| Perillaldehyde | 16441 |
| Perillene | 68316 |
| Perillic acid | 1256 |
| Perillyl alcohol | 10819 |
| Piperitenone | 381152 |
| p-Menth-1-en-4-ol | 11230 |
| p-Menth-1-en-8-ol | 17100 |
| p-Mentha-3,8-diene | 521851 |
| Pulegone | 442495 |
| Phytol | 5280435 |
| Phytone | 10408 |
| Sabinene | 18818 |
| Santolina triene | 519872 |
| Spathulenol | 92231 |

| | | | | |
|---|---|---|---|---|
| | | Terpinen-4-ol | 11230 | |
| | | Terpinolene | 11463 | |
| | | Thujyl alcohol | 10550 | |
| | | trans-Nerolidol | 5284507 | |
| | | trans-Shisool | 519954 | |
| | | Triacontane | 12535 | |
| | | Tridecane | 12388 | |
| | | Valencene | 9855795 | |
| | | Valeric acid, pent-2-en-4-ynyl ester | 5353036 | |
| | | Viridiflorene | 10910653 | |
| | | Viridiflorol | 11996452 | |
| | | α-Bulnesene | 520826 | |
| | | α-Cadinol | 10398656 | |
| | | α-Caryophyllene | 5281520 | |
| | | α-Citral | 638011 | |
| | | α-Copaene | 70678558 | |
| | | α-Farnesene | 5281516 | |
| | | α-Fenchene | 12309839 | |
| | | α-Patchoulene | 521710 | |
| | | α-Santalol | 5281531 | |
| | | β-Cubebene | 93081 | |
| | | β-Bourbonene | 324224 | |
| | | β-Cadinene | 10657 | |
| | | β-Cyclocitral | 9895 | |
| | | β-Elemene | 6918391 | |
| | | β-Farnesene | 5281517 | |
| | | β-Guaiene | 6949 | |
| | | β-Gurjunene | 6450812 | |
| | | β-Ionone | 638014 | |
| | | β-Pinene | 14896 | |
| | | β-Selinene | 442393 | |
| | | β-Phellandrene | 11142 | |
| | | β-Terpinene | 66841 | |
| | | γ-Pyronene | 578237 | |
| | | δ-Cadinene | 441005 | |
| | | δ-Elemene | 12309449 | 67 |
| 27. | Aralia cordata (10) | Methyl-α-D-fructofuranoside | 6325664 | 68 |
| | | Methyl-β-D-fructofuranoside | 128889 | 68 |
| | | 3,5-Di-O-caffeoylquinic acid | 6474310 | 68 |
| | | Pimaric acid | 220338 | 69 |
| | | (-)-Kaur-16-en-19-oic acid | 73062 | 69 |
| | | Falcarindiol | 5281148 | 69 |
| | | 17-Hydroxy-ent-kaur-15-en-19-oic acid | 169654 | 69 |
| | | Alpha-mono palmitin | 14900 | 69 |
| | | Kaurenoic acid | 73062 | 70 |
| | | Continentalic acid | 10086296 | 70 |
| 28. | Anemarrhena asphodeloides (40) | Neomangiferin | 6918448 | 71 |
| | | Mangiferin | 5281647 | |
| | | Isomangiferin | 5318597 | |
| | | Vitexin | 5280441 | |

| | | | | |
|---|---|---|---|---|
| | | Isosakuranetin | 160481 | |
| | | Macrostemonoside J | 101669618 | |
| | | Asparagoside G | 3042722 | |
| | | Tomatoside A | 426059 | |
| | | Timosaponin BII | 44575945 | |
| | | Officinalisinin-I | 441889 | |
| | | Timosaponin D | 132545772 | |
| | | Filicinoside-A | 190854 | |
| | | Timosaponin F | 101005526 | |
| | | Anemarrhenasaponin I | 101672279 | |
| | | Anemarrhenasaponin II | 101672380 | |
| | | Anemarrhenasaponin III | 101672280 | |
| | | F-gitonin | 44559009 | |
| | | Timosaponin AIV | 154572708 | |
| | | Timosaponin AIII | 15953793 | |
| | | Desgalactotigonin | 162401 | |
| | | Timosaponin AI | 71767755 | |
| | | cis-hinokiresinol | 5281830 | |
| | | monomethyl-cis-hinokiresinol | 5319736 | |
| | | Foliamangiferoside A | 46206548 | |
| | | 2,6,4′-trihydroxy-4- methoxybenzophenone | 10467773 | |
| | | Phytosphingosine | 122121 | |
| | | Dimethisterone | 6607 | |
| | | Macrostemonoside F | 192523 | 72 |
| | | Platycodin D | 162859 | 72 |
| | | Platycoside A | 50900942 | 72 |
| | | Platycodin D2 | 53317652 | 72 |
| | | Polygalacin D2 | 53325781 | 72 |
| | | Platycodin D3 | 70698293 | 72 |
| | | trans-Hinokiresinol | 12310493 | 72 |
| | | Isosarsasapogenin | 91439 | 73 |
| | | Markogenin | 12304414 | 73 |
| | | Neogitogenin | 12304409 | 73 |
| | | Sarsasapogenin | 92095 | 73 |
| | | Diosgenin | 99474 | 73 |
| 29. | Arctium lappa (10) | Arctigenin | 64981 | 74 |
| | | Arctiin | 100528 | 74 |
| | | Trachelogenin | 452855 | 74 |
| | | Lappaol F | 73425459 | 74 |
| | | Diarctigenin | 16215736 | 74 |
| | | Methyl palmitate | 8181 | 75 |
| | | Methyl linoleate | 5284421 | 75 |
| | | Methyl oleate | 5364509 | 75 |
| 30. | Artemisia princeps (20) | Hexanal | 6184 | 76 |
| | | Benzeneacetaldehyde | 998 | |
| | | cis-Sabinene hydrate | 101629835 | |
| | | trans-Sabinene hydrate | 12315151 | |
| | | cis-Verbenol | 164888 | |
| | | trans-Verbenol | 89664 | |
| | | cis-Chrysanthenol | 527032 | |

| | | | | |
|---|---|---|---|---|
| | | Verbenone | 29025 | |
| | | Chrysanthenyl acetate | 162747 | |
| | | Lavandulyl acetate | 30247 | |
| | | Silphiperfol-5-ene | 91747334 | |
| | | Germacrene D | 5317570 | |
| | | Zingiberene | 92776 | |
| | | Germacrene D 4-ol | 5352847 | |
| | | Neophytadiene | 10446 | |
| | | Hexadecanoic acid | 985 | |
| | | Phytol | 5280435 | |
| | | Nonacosane | 12409 | |
| | | Hentriacontane | 12410 | |
| | | Dotriacontane | 11008 | |
| | | Tritriacontane | 12411 | |
| 31. | Pinellia ternate (75) | Isolariciresinol | 160521 | 77 |
| | | isolariciresinol 9-O-beta-D-glucopyranoside | 85210374 | 77 |
| | | pinoresinol-4-O-beta-D glucoside | 486614 | 77 |
| | | dehydrodiconiferyl alcohol 4-O-b-D-glucopyranoside | 5316442 | 77 |
| | | neo-olivil | 9976812 | 77 |
| | | medioresinol | 181681 | 77 |
| | | americanol A | 637304 | 77 |
| | | pinoresinol | 73399 | 77 |
| | | burselignan | 11631864 | 77 |
| | | L-Ephedrine | 9294 | 78 |
| | | Inosine | 135398641 | 78 |
| | | Cytidine | 6175 | 78 |
| | | Pedatisectine B | 190 | 78 |
| | | 8-Octadecenoic acid | 5282758 | 78 |
| | | Pentadecanoic acid | 13849 | 78 |
| | | 9-Hexadecenoic acid | 5282745 | 78 |
| | | Hexadecanoic acid | 985 | 78 |
| | | Heptadecanoic acid | 10465 | 78 |
| | | 7-Hexadecenoic acid | 543268 | 78 |
| | | Octadecanoic acid | 5281 | 78 |
| | | 9-Oxo-nonanoic acid | 75704 | 78 |
| | | 11-Eicosenoic acid | 5282768 | 78 |
| | | Eicosanoic acid | 10467 | |
| | | 10,13-Eicosadienoic acid | 72444876 | |
| | | Docosanoic acid | 8215 | |
| | | Pinellic acid | 9858729 | |
| | | Succinic acid | 1110 | |
| | | 3-Methyleicosane | 98417 | |
| | | 3-Decyne | 75425 | |
| | | 2-Methyldecane | 23415 | |
| | | Octadecane | 11635 | |
| | | 2,6,10-Trimethyltetradecane | 85785 | |
| | | 2,5-Dimethyltetradecane | 41836 | |
| | | Vinylcyclohexane | 12757 | |
| | | 1-Octene | 8125 | |

| | | | | |
|---|---|---|---|---|
| | | 6-Methyl-2-heptanone | 13572 | |
| | | 3-Nonanone | 61235 | |
| | | Cis-4-decenal | 5362620 | |
| | | 2-Undecanone | 8163 | |
| | | 9-Heptadecanol | 136435 | |
| | | Anethole | 637563 | |
| | | Citronellal | 7794 | |
| | | Aromandendrene | 91354 | |
| | | Farnesane | 19773 | |
| | | β-Patchoulene | 101731 | |
| | | $\alpha$-Elemol | 92138 | |
| | | 1-Methyl-4-(1-methylethenyl)-cyclohexene | 6453304 | |
| | | Dibutyl phthalate | 3026 | |
| | | 2,6-Di-tert-butyl-4-methylphenol | 31404 | |
| | | 2-Pentylfuran | 19602 | |
| | | Furfural | 7362 | |
| | | 2,4-Dimethyl furan | 19462 | 78 |
| | | Anisic acid | 7478 | |
| | | Pulegone | 442495 | |
| | | Isopulegol | 170833 | |
| | | 3-Nonyne | 140649 | |
| | | 2-Methylnonane | 13379 | |
| | | 3,4-Dihydroxycinnamyl alcohol | 5282096 | |
| | | Sachaliside 1 | 14048613 | |
| | | Stigmast-4-en-3-one | 5484202 | |
| | | Cycloartenol | 92110 | |
| | | Protocatechuic aldehyde | 8768 | |
| | | Shogaol | 5281794 | |
| | | Gingerol | 442793 | |
| | | Erythritol | 222285 | |
| | | Melissane | 182192 | |
| | | Nonacosane | 12409 | |
| | | Homogentisic acid | 780 | |
| | | Benzene-1,4-diol | 785 | |
| | | Benzene-1,2-diol | 289 | |
| | | Monogalactosyldiacylglycerol | 90657729 | |
| | | Soyacerebroside I | 11104507 | |
| | | Soyacerebroside II | 15599558 | |
| | | N-acetylglutamate | 70914 | |
| 32. | Platycodon grandifloras (55) | Platycoside A | 50900942 | 79 |
| | | Platycoside B | 101937508 | |
| | | Platycoside C | 101937509 | |
| | | Platycoside D | 100974704 | |
| | | Platycoside E | 70698202 | |
| | | Platycoside G1 | 70698289 | |
| | | Platycoside F | 101048500 | |
| | | Platycoside G2 | 102217526 | |
| | | Platycoside G3 | 70698263 | |
| | | Platycoside H | 11665339 | |
| | | Platycoside I | 11622299 | |

| | | | |
|---|---|---|---|
| | Platycoside J | 11528185 | |
| | Platycoside K | 102004765 | |
| | Platycoside L | 11556931 | |
| | Platycoside M-1 | 101403595 | |
| | Platycoside M-2 | 101403596 | |
| | Platycoside M-3 | 101403597 | |
| | Platycodin A | 46173910 | |
| | Platycodin C | 46173919 | |
| | Platycodin D | 162859 | |
| | Deapioplatycodin D | 70698266 | |
| | Platycodin D2 | 53317652 | |
| | Deapioplatycodin D2 | 50900942 | |
| | Platycodin J | 102052427 | |
| | Platycodin K | 102052428 | |
| | Platycodin L | 102052429 | |
| | 3-O-β-D-glucopyranosylplatycodigenin | 102185205 | |
| | Polygalacin D | 46173909 | |
| | 20-O-acetylPolygalacin D | 53321792 | |
| | 30-O-acetylPolygalacin D | 53321793 | |
| | Polygalacin D2 | 53325781 | |
| | 30-O-acetylPolygalacin D2 | 53321794 | |
| | Platyconic acid A | 70698300 | |
| | Platyconic acid B lactone | 50900852 | |
| | Deapioplatyconic acid B lactone | 50900941 | 79 |
| | Platycogenic acid A | 20056221 | |
| | Platyconic acid B | 101495504 | |
| | Platyconic acid C | 102052424 | |
| | Platyconic acid D | 102052425 | |
| | Platyconic acid E | 102052426 | |
| | Platycogenic acid B | 69570716 | |
| | Platycogenic acid C | 69569686 | |
| | Platyconin | 90659256 | |
| | (2R, 3R) taxifolin | 439533 | 79 |
| | quercetin-7-O-glucoside | 5282160 | 79 |
| | quercetin-7-O-rutinoside | 101764560 | 79 |
| | luteolin-7-O-glucoside | 5280637 | 79 |
| | apigenin-7-O-glucoside | 12304093 | 79 |
| | p-hydroxybenzoic acid | 135 | 79 |
| | 2,3-dihydroxybenzoic acid | 19 | 79 |
| | 2-hydroxy4-methoxybenzoic acid | 75231 | 79 |
| | homovanillic acid | 1738 | 79 |
| | spinasterol | 5281331 | 79 |
| | botulin | 49895112 | 79 |
| | δ-7-stigmastenone-3 | 5748344 | 79 |
| | Apigenin | 5280443 | 79 |
| | Luteolin | 5280445 | 79 |
| | Platycoside | 101403597 | 79 |
| | Flavoplatycoside | 10416329 | 79 |
| | 3,4-dimethoxycinnamic acid | 717531 | 79 |
| | Isoferulic acid | 736186 | 79 |

| | | | | |
|---|---|---|---|---|
| | | m-coumaric acid | 637541 | 79 |
| | | p-coumaric acid | 637542 | 80 |
| | | α-resorcylic acid | 7424 | 80 |
| | | Chlorogenic acid | 1794427 | 80 |
| | | Lobetyolin | 53486204 | 80 |
| | | Lobetyolinin | 5459227 | 80 |
| | | Iobetyol | 5807986 | 80 |
| | | Betulin | 72326 | 80 |
| | | β-sitosterol | 521199 | 80 |
| | | Inulin | 24763 | 80 |
| | | Grandoside | 10341593 | 80 |
| 33. | Rehmannia glutinosa (45) | Lariciresinol | 332427 | 81 |
| | | hierochin D | 85382912 | 81 |
| | | yemuoside 1 | 195850 | 81 |
| | | darendoside B | 21668724 | 81 |
| | | decaffeoyl acteoside | 13889681 | 81 |
| | | jionoside $B_1$ | 5281782 | 81 |
| | | catalpol | 91520 | 81 |
| | | ajugol | 6325127 | 81 |
| | | 6-*O*-vanilloylajugol | 14396664 | 81 |
| | | 6-*O*-*E*-feruloylajugol | 6325178 | 81 |
| | | Rehmapicroside | 21637711 | 81 |
| | | Rehmapicrogenin | 15693863 | 82 |
| | | hydroferulic acid | 14340 | 82 |
| | | aucubin | 91458 | 82 |
| | | isoacteoside | 6476333 | 82 |
| | | acteoside | 5281800 | 82 |
| | | ethyl β-D-fructofuranoside | 138113708 | 82 |
| | | eleutheroside C | 9859136 | 82 |
| | | mannitol | 6251 | 82 |
| | | raffinose | 439242 | 82 |
| | | stachyose | 439531 | 82 |
| | | glutinolic acid | 53248310 | 82 |
| | | paulownin | 3084131 | 82 |
| | | monopalmitin | 14900 | 82 |
| | | pinellic acid | 9858729 | 82 |
| | | jio-cerebroside | 10169092 | 82 |
| | | aeginetic acid 5-O-beta-D-quinovoside | 53248311 | 83 |
| | | aeginetoyl ajugol 5''-O-beta-D-quinovoside | 101790066 | 83 |
| | | rehmaionoside A | 10023290 | 83 |
| | | dihydroxy-beta-ionone | 15693868 | 83 |
| | | rehmaglutin D | 5320906 | 83 |
| | | ajugoside | 9865184 | 83 |
| | | martynoside | 5319292 | 84 |
| | | leucosceptoside A | 10394343 | 84 |
| | | 5-hydroxymethyl furfural | 237332 | 84 |
| | | Tyrosol | 10393 | 84 |
| | | Isomartynoside | 91895373 | 84 |
| | | purpureaside C | 11953944 | 84 |
| | | jionoside A1 | 6325450 | 84 |

| | | | | |
|---|---|---|---|---|
| | | ferulic acid methyl ester | 5357283 | 85 |
| | | coniferin | 5280372 | 85 |
| | | leonoside F | 57325811 | 85 |
| | | echinacoside | 5281771 | 85 |
| | | diincarvilone A | 60155233 | 85 |
| | | pterolactam | 181561 | 85 |
| | | 3-indolecarboxylic acid | 69867 | 85 |
| 34. | Saposhnikovia divaricate (45) | Ledebouriellol | 5318962 | 86 |
| | | Hamaudol | 164722 | |
| | | sec-O-glucosylhamaudol | 10478277 | |
| | | Divaricatol | 9974111 | |
| | | Cimifugin | 441960 | |
| | | prim -O – glucosylcimifugin | 14034912 | |
| | | 5-O-methylvisamminol | 441970 | |
| | | 4'-O-β-D-glucosyl-5-O-methylvisamminol | 21670038 | |
| | | Norcimifugin | 46240156 | |
| | | undulatoside A | 5321494 | |
| | | wogonin | 5281703 | |
| | | bergapten | 2355 | |
| | | byakangelicin | 10211 | |
| | | deltoin | 906525 | |
| | | imperatorin | 10212 | |
| | | isoimperatorin | 68081 | |
| | | isobergapten | 68082 | |
| | | marmesin | 334704 | |
| | | nodakenetin | 26305 | |
| | | oxypeucedanin hydrate | 17536 | |
| | | phellopterin | 98608 | |
| | | psoralen | 6199 | |
| | | xanthotoxin | 4114 | |
| | | 5-hydroxy-8-methoxypsoralen | 5385192 | |
| | | Nodakenin | 73191 | |
| | | Xanthoarnol | 11482406 | |
| | | Fraxidin | 3083616 | |
| | | Isofraxidin | 5318565 | |
| | | Scopoletin | 5280460 | |
| | | Umbelliferone | 5281426 | |
| | | Anomalin | 10251869 | |
| | | Decursinol | 442127 | |
| | | decursinol angelate | 776123 | |
| | | praeruptorin B | 5319259 | |
| | | praeruptorin F | 51668830 | |
| | | cis-3', 4'-disenecioylkhellactone | 1119128 | |
| | | (–)-cis-khellactone | 455821 | |
| | | (3'S)-hydroxydeltoin | 455821 | |
| | | Panaxynol | 5281149 | |
| | | Falcarindiol | 5281148 | |
| | | (9Z)-1-methoxy9-heptadecene-4,6-diyn-3-ol | 146026404 | |
| | | Marmesinin | | |
| | | tectochrysin | 216283 | |

| | | | | |
|---|---|---|---|---|
| | | glyceryl monooleate | 5281954 | |
| | | 8'-epicleomiscosin A | 5283468 | |
| | | | 11200016 | 86 |
| 35. | Glycyrrhiza uralensis (30) | lignoceric acid | 11197 | 87 |
| | | licorisoflavan A | 196831 | |
| | | methoxyficifolinol | 480872 | |
| | | docosanol | 12620 | |
| | | betulinic acid | 64971 | |
| | | licoricidin | 480865 | |
| | | kumatakenin | 5318869 | |
| | | docosyl caffeate | 5316952 | |
| | | neoglycyrol | 5320083 | |
| | | gancaonin H | 5481949 | |
| | | isoglycyrol | 124050 | |
| | | liquiritigenin | 114829 | |
| | | lupiwighteone | 5317480 | |
| | | 3*R*-vestitol | 439310 | |
| | | Echinatin | 6442675 | |
| | | Glycycoumarin | 5317756 | |
| | | Licoarylcoumarin | 10090416 | |
| | | 7,2',4'-trihydroxy-5-methoxy-3-arylcoumarin | 25015742 | |
| | | Glicoricone | 10361658 | |
| | | Isoliquiritigenin | 638278 | |
| | | Formononetin | 5280378 | |
| | | Licoricone | 5319013 | |
| | | Glycyrin | 480787 | |
| | | formononetin-7-*O*-*β*-*D*-glucoside | 101350244 | |
| | | liquiritin | 503737 | |
| | | isoliquiritin apioside | 6442433 | |
| | | glycyrrhizic acid | 14982 | |
| | | liquiritin apioside | 10076238 | |
| | | hedysarimcoumestan B | 11558452 | |
| 36. | Ziziphus jujuba (70) | Mauritine A | 11353668 | 88 |
| | | Mucronine D | 5373023 | |
| | | Amphibine H | 51029223 | |
| | | Nummularine A | 131750852 | |
| | | Nummularine B | 51017057 | |
| | | sativanine-B | 5281595 | |
| | | frangulanine | 5281587 | |
| | | sativanine F | 102156891 | |
| | | coclaurine | 160487 | |
| | | isoboldine | 133323 | |
| | | norisoboldine | 14539911 | |
| | | asimilobine | 160875 | |
| | | sanjoinenine | 14729078 | |
| | | amphibine-D | 5318120 | |
| | | sanjoinine-B | 14729076 | |
| | | sanjoinine-D | 44566617 | |
| | | sanjoinine-F | 14729081 | |

| | | | |
|---|---|---|---|
| | Jubanine-A | 101316794 | |
| | Jubanine-B | 101316795 | |
| | Scutianine-C | 45268911 | |
| | Scutianine-D | 45267179 | |
| | Jubanine-C | 131752168 | |
| | Zizyphine-A | 6324833 | |
| | 6'''- sinapoylspinosin | 101616450 | 88 |
| | 6'''-feruloylspinosin | 21597353 | 88 |
| | 6'''-pcoumaroylspinosin | 5316122 | 88 |
| | jujuboside A | 51346169 | 88 |
| | jujuboside B | 24721031 | 88 |
| | jujuboside A1 | 177396 | 88 |
| | jujuboside B1 | 73156987 | 88 |
| | jujuboside C | 71448946 | 88 |
| | acetyljujuboside B | 73093046 | 88 |
| | protojujuboside A | 71448943 | 88 |
| | protojujuboside B | 71448944 | 88 |
| | protojujuboside B1 | 71448945 | 88 |
| | Puerarin | 5281807 | 88 |
| | 6'''-feruloylisospinosin | 10795344 | 88 |
| | Isospinosin | 11801844 | 88 |
| | Kaempferol 3-O-robinobioside | 15944778 | 88 |
| | Kaempferol 3-O-rutinoside | 5318767 | 88 |
| | colubrinic acid | 21672700 | 88 |
| | alphitolic acid | 12305768 | 88 |
| | zizyberenalic acid | 15958448 | 88 |
| | betulinic acid | 64971 | 88 |
| | 3-*O*-(*cis*-*p*-coumaroyl)-alphitolic acid | 5316118 | 89 |
| | 3-beta-*O*-(*trans*-*p*-coumaroyl)maslinic acid | 14335962 | 89 |
| | pomonic acid | 12314449 | 89 |
| | 2-oxopomolic acid | 44593379 | 89 |
| | benthamic acid | 3838010 | 89 |
| | terminic acid | 132568257 | 89 |
| | traumatic acid | 5283028 | 89 |
| | (*Z*)-4-oxotetradec-5-enoic acid | 132568256 | 89 |
| | Magnoflorine | 73337 | 89 |
| | Franganine | 45270592 | 90 |
| | Spinosyn | 155692 | |
| | 3beta,6beta-stigmast-4-en-3,6-diol | 146156493 | |
| | Maslinic acid | 73659 | |
| | Ceanothic acid | 161352 | |
| | Vitexin | 5280441 | |
| | Oleamide | 5283387 | |
| | Ent-epicatechinoceanothic acid A | 132967447 | |
| | Lupeol | 259846 | |
| | 2-alpha-hydroxyursolic acid | 6918774 | |
| | Ziziphin | 441957 | |
| | Squalene | 638072 | |
| | epiceanothic acid | 23631167 | |
| | ceanothenic acid | 71451218 | |

| | | | | |
|---|---|---|---|---|
| | | zizyberanalic acid | 21672700 | |
| | | ursonic acid | 9890209 | |
| 37. | Pueraria lobata (50) | apigenin triacetate | 18721 | 91 |
| | | daidzein diacetate | 10359753 | |
| | | daidzein dimethyl ether | 136419 | |
| | | daidzin | 107971 | |
| | | puerarin | 5281807 | |
| | | daidzein | 5281708 | |
| | | formononetin | 5280378 | |
| | | puerarol | 44257531 | |
| | | 3'-Hydroxypuerarin | 5748205 | |
| | | 3'-Methoxydaidzein | 5319422 | |
| | | 3'-methoxydaidzin | 10527347 | |
| | | 3′-methoxypuerarin | 5319485 | |
| | | 6''-O-D-Xylosylpuerarin | 101776119 | |
| | | biochanin A | 5280373 | |
| | | 4'-Methoxypuerarin | 5319486 | |
| | | 3'-Methoxypuerarin | 5319485 | |
| | | mirificin | 21676217 | |
| | | ononin | 442813 | |
| | | puerarin-4'-O-glucoside | 44257212 | |
| | | irisolidone | 5281781 | |
| | | kaikasaponin I | 102120183 | |
| | | soyasaponin I | 122097 | 91 |
| | | astragaloside VIII | 124491170 | 91 |
| | | azukisaponin I | 14103656 | 91 |
| | | baptisiasaponin I | 102317160 | 91 |
| | | genistein | 5280961 | 91 |
| | | genistin | 5281377 | 91 |
| | | glycitein | 5317750 | 91 |
| | | glycitin | 187808 | 91 |
| | | kaikasaponin II | 101538997 | 91 |
| | | kaikasaponin III | 188384 | 91 |
| | | kakkalide | 5490351 | 91 |
| | | kakkasaponin I | 100945993 | 91 |
| | | kakkasaponin II | 102500447 | 91 |
| | | kakkasaponin III | 102500448 | 91 |
| | | luteolin | 5280445 | 91 |
| | | phaseoside IV | 101679107 | 92 |
| | | soyasaponin IV | 24721354 | 92 |
| | | tectorigenin | 5281811 | 92 |
| | | tectorigenin-7-O-xylosylglucoside | 100968221 | 92 |
| | | nicotiflorin | 5318767 | 92 |
| | | robinin | 5281693 | 92 |
| | | beta-sitosterol palmitate | 13747834 | 92 |
| | | lupeone | 92158 | 92 |
| | | diisobutyl phthalate | 6782 | 92 |
| | | bis(2-ethylhexyl) phthalate | 8343 | 92 |
| | | sophoracoumestan A | 14630492 | 92 |
| | | coumestrol | 5281707 | 92 |

| | | | | |
|---|---|---|---|---|
| | | allantion | 439714 | 92 |
| | | Sissotorin | 73407546 | 92 |
| | | (6S,9R)-roseoside | 9930064 | 92 |
| 38. | Coix lacryma-jobi (15) | Coixenolide | 46173943 | 93 |
| | | γ-Tocopherol | 14985 | 93 |
| | | Docosanol | 12620 | 93 |
| | | 4-Ketopinoresinol | 44578390 | 93 |
| | | p-Hydroxy benzaldehyde | 126 | 93 |
| | | Tangeretin | 68077 | 93 |
| | | Naringenin | 932 | 93 |
| | | Nobiletin | 72344 | 93 |
| | | Neolignan | 261166 | 93 |
| | | 5-Amino-1-(quinolin-8-yl)-1,2,3-triazole-4-carboxamide | 26435789 | 94 |
| | | Pyridine | 1049 | 94 |
| | | Propenamide | 6579 | 94 |
| | | N-methoxy-N-methyl-3,4-dihydro-2H-thiopyran6-carboxamide | 24813996 | 94 |
| | | 1,3-dioctanoin | 150911 | 94 |
| | | tetradecanoic acid | 11005 | 94 |
| 39. | Rheum palmatum (22) | aloe-emodin | 10207 | 95 |
| | | chrysophanol | 10208 | |
| | | emodin | 3220 | |
| | | emodin-6-O-beta-D-glucopyranoside | 5317038 | |
| | | physcion | 10639 | |
| | | physcion-8-O-beta-D-glucopyranoside | 5319323 | |
| | | rhein | 10168 | |
| | | rhein-8-O-β-D-glucopyranoside | 146156489 | |
| | | palmidin A | 5320384 | |
| | | palmidin B | 5320385 | |
| | | palmidin C | 5320386 | |
| | | rheinoside A | 13888123 | |
| | | rheinoside C | 13888128 | |
| | | sennidin C | 5321255 | |
| | | sennoside A | 73111 | |
| | | sennoside B | 91440 | |
| | | sennoside C | 46173829 | |
| | | sennoside D | 46173830 | |
| | | rhapontigenin | 5320954 | |
| | | gallic acid 3-O-β-D-glucopyranoside | 101683334 | |
| | | gallic acid 4-O-β-D-glucopyranoside | 10088114 | |
| | | coumaroyl-O-galloyl-glucose | 146170723 | |
| 40. | Uncaria rhynchophylla (30) | Hirsutine | 3037884 | 96 |
| | | Hirsuteine | 3037151 | |
| | | epi-allo-corynantheine | 101603230 | |
| | | corynantheidine | 3000341 | |
| | | dihydrocorynantheine | 3039336 | |
| | | akuammigine | 1268096 | |
| | | yohimbine | 8969 | |
| | | angustine | 441983 | |

| | | | | |
|---|---|---|---|---|
| | | angustoline | 3084765 | |
| | | angustidine | 3084770 | |
| | | corynoxeine | 44568160 | |
| | | 18,19-dehydrocorynoxinic acid | 24970642 | |
| | | Isorhynchophylline | 3037048 | |
| | | Isocorynoxeine | 3037448 | |
| | | 18,19-dehydrocorynoxinic acid B | 24970641 | |
| | | cadambine | 398038 | |
| | | 3 Alpha-dihydrocadambine | 162138 | |
| | | vincoside lactam | 44567645 | |
| | | rhynchophine | 11972452 | |
| | | strictosidine | 161336 | |
| | | strictosamide | 10345799 | |
| | | 6beta-hydroxyursolic acid | 20055642 | |
| | | uncarinic acid C | 44583694 | |
| | | uncarinic acid D | 44583694 | |
| | | uncarinic acid B | 100967916 | |
| | | uncarinic acid E | 10746421 | |
| | | trifolin | 5282149 | |
| | | hyperin | 5281643 | |
| | | cleomiscosin B | 156875 | |
| | | cleomiscosin D | 13965876 | |
| 41. | Forsythia suspensa (90) | (+)-pinoresinol | 73399 | 97 |
| | | (+)-phillygenin | 4166098 | |
| | | Phillyrin | 101712 | |
| | | (+) epipinoresinol | 637584 | |
| | | Pinoresinol | 73399 | |
| | | 8- hydroxypinoresinol | 3010930 | |
| | | benzenebutanoic acid | 4775 | |
| | | Arctigenin | 64981 | |
| | | (−) dimethylmatairesinol | 1286 | |
| | | Arctiin | 100528 | |
| | | (–)- matairesinoside | 486612 | |
| | | Olivil | 5273570 | |
| | | (+) lariciresinol | 332427 | |
| | | forsythialan A | 44453412 | |
| | | forsythialan B | 44453332 | |
| | | (–)-egenine | 189685 | |
| | | (–)-bicuculline | 185838 | |
| | | Rutaecarpine | 65752 | |
| | | p-hydroxyphenylacetic acid | 127 | |
| | | suspenolic acid | 10774324 | |
| | | Trans-nerolidol | 5284507 | |
| | | 2,4-di-tert-butylphenol | 7311 | |
| | | estragole | 8815 | |
| | | p-tyrosol | 10393 | |
| | | hydroxytyrosol | 82755 | |
| | | Sasanquin | 5317538 | |
| | | tannic acid | 16129778 | |
| | | esculetin | 5281416 | |

| | | |
|---|---|---|
| | cedrusin | 11210164 |
| | glochidioboside | 24982202 |
| | icariside E4 | 21589939 |
| | Isolariciresinol | 160521 |
| | (+)-isoolivil | 5316262 |
| | Forsythiaside (forsythoside A) | 5281773 |
| | forsythoside D | 24721571 |
| | forsythoside E | 69634125 |
| | salidroside | 159278 |
| | forsythoside I | 23958169 |
| | calceolarioside A | 5273566 |
| | Suspensaside A | 132550846 |
| | R-suspensaside | 102228838 |
| | S-suspensaside | 102228839 |
| | forsythoside B | 23928102 |
| | forsythoside F | 6442994 |
| | forsythoside G | 101231533 |
| | forsythoside H | 129449684 |
| | calceolarioside B | 5273567 |
| | plantainoside A | 5320625 |
| | plantainoside B | 9847922 |
| | calceolarioside C | 45360240 |
| | Wogonin-7-O-glucoside | 51136398 |
| | Hesperidin | 10621 |
| | Hyperin | 5281643 |
| | taraxasterol acetate | 13889352 |
| | nigaichigoside F1 | 16118969 |
| | quadranoside IV | 10372074 |
| | esculentic acid | 9898760 |
| | corosolic acid | 6918774 |
| | onjisaponin F | 10701737 |
| | arjunglucoside I | 14658050 |
| | arjunglucoside II | 52951052 |
| | hovenic acid | 70698002 |
| | alphitolic acid | 12305768 |
| | isobauerenyl acetate | 181096 |
| | Ocotillone | 12313665 |
| | garcinielliptone Q | 102452579 |
| | agatholic acid | 101306703 |
| | 3- oxoanticopalic acid | 13858184 |
| | haplopappic acid | 102117108 |
| | dehydropinifolic acid | 22568711 |
| | adoxosidic acid | 13892717 |
| | Alpha-thujene | 17868 |
| | sabinene | 18818 |
| | $\gamma$-terpinene | 7461 |
| | terpinolene | 11463 |
| | (+)-carene | 442461 |
| | $\alpha$-ocimene | 5320249 |
| | terpinen-4-ol | 11230 |

| | | | | |
|---|---|---|---|---|
| | | p-cymen-8-ol | 14529 | |
| | | trans-carveol | 94221 | |
| | | (-)-myrtenal | 1201529 | |
| | | cornoside | 11809239 | |
| | | rengyolone | 10725564 | |
| | | forsythenside A | 11797786 | |
| | | forsythenside B | 10576163 | |
| | | Rengyoside B | 11045420 | |
| | | (6S,9R)- roseoside | 129316932 | |
| | | Rengyol | 363707 | |
| | | Rengyoxide | 14353410 | |
| | | rengyoside A | 10958239 | |
| | | rengynic acid | 54033324 | |
| 42. | Alisma orientale (60) | Alisol A | 15558616 | 98 |
| | | Alisol B | 15558620 | |
| | | Alisol C | 46173914 | |
| | | Alisol E (epi-alisol A) | 101297679 | |
| | | Alisol F | 76310822 | |
| | | Alisol G ( 25-Anhydro-alisol A) | 76314443 | |
| | | Alisol O | 102004738 | |
| | | Alisol P | 101840124 | |
| | | Alisol A 23-acetate | 70690607 | |
| | | Alisol B 23-acetate | 14036811 | |
| | | Alisol C 23-acetate | 14036813 | |
| | | Alisol E 23-acetate | 101661272 | |
| | | Alisol J 23-acetate | 100936564 | |
| | | Alisol K 23-acetate | 100936565 | |
| | | Alisol L 23-acetate | 100936566 | |
| | | Alisol M 23-acetate | 70688546 | |
| | | Alisol N 23-acetate | 100936567 | |
| | | Alisol A 24-acetate | 76336194 | |
| | | Alismaketone A 23-acetate | 11800363 | |
| | | Alismaketone B 23-acetate | 100941986 | |
| | | Alismaketone C 23-acetate | 100941987 | |
| | | Alismalactone 23-acetate | 101701562 | |
| | | Alisolide | 101840122 | |
| | | 11-Deoxy-alisol B 23-acetate | 101661275 | |
| | | 11-Deoxy-alisol C 23-acetate | 101664412 | |
| | | 13β,17β-Epoxy-alisol B | 101664542 | |
| | | 25-Anhydro-alisol A 11-acetate | 5318945 | |
| | | 25-Anhydro-alisol F | 102004739 | |
| | | 16-Oxo-alisol A | 9983614 | |
| | | 16,23-Oxido-alisol B | 9847547 | |
| | | 25-O-methyl-alisol A | 102004721 | |
| | | Orientalol E | 637282 | |
| | | Orientalol F | 11020886 | |
| | | Orientanone | 11727780 | |
| | | Sulfoorientalol B | 5321551 | |
| | | Sulfoorientalol C | 5321552 | |
| | | Sulfoorientalol D | 146033487 | |

| | | | | |
|---|---|---|---|---|
| | | Alismorientol A | 16742799 | |
| | | Alismorientol B | 16742798 | |
| | | Alismol | 5318916 | |
| | | Alismoxide | 10988340 | |
| | | Clovandiol | 76319362 | |
| | | Germacrene D | 5317570 | |
| | | Germacrene C | 25244915 | |
| | | Oplopanone | 10466745 | |
| | | Oriediterpenol | 5320304 | |
| | | Oriediterpenoside | 6325764 | |
| | | Robustaflavone | 5281694 | |
| | | Amentoflavone | 5281600 | |
| | | Calycosin | 5280448 | |
| | | 7-Hydroxy-coumarin | 5281426 | |
| | | Emodin | 3220 | |
| | | Falcalindiol | 6436239 | |
| | | Isoimperatorin | 68081 | |
| | | Seselin | 68229 | |
| | | Dulcitol | 11850 | |
| | | Stachyose | 439531 | |
| | | Verbascose | 441434 | |
| | | Manninotriose | 5461026 | |
| | | Verbascotetraose | 102273024 | |
| 43. | Amomum xanthioides (45) | (+)-5-endo-hydroxycamphor | 9543191 | 99 |
| | | protocatechuic acid methyl ester | 287064 | 99 |
| | | betulabuside A | 14484636 | 99 |
| | | hedychiol A | 10105633 | 100 |
| | | pygmol | 14167388 | 100 |
| | | dihydroyashabushiketol | 10265808 | 100 |
| | | Tricyclene | 79035 | 101 |
| | | o-Cymene | 10703 | 101 |
| | | 1,8-Cineole | 2758 | 101 |
| | | (E)-β-Ocimene | 5281553 | 101 |
| | | α-Terpinolene | 11463 | 101 |
| | | Camphor | 2537 | 101 |
| | | 2,6-Dimethyl-2,4,6-octatriene | 5368821 | 101 |
| | | Terpinen-4-ol | 11230 | 101 |
| | | Fenchyl acetate | 107217 | 101 |
| | | Bornyl acetate | 6448 | 101 |
| | | Bicycloelemene | 56842786 | 101 |
| | | Dodecamethyl-cyclohexasiloxane | 10911 | 101 |
| | | α-Cubebene | 442359 | 101 |
| | | β-Cubebene | 93081 | 101 |
| | | Alpha-Gurjunene | 15560276 | |
| | | Beta-Gurjunene | 6450812 | |
| | | Aromadendrene | 91354 | |
| | | γ-Gurjunene | 15560285 | 101 |
| | | Germacrene D | 5317570 | |
| | | α-Amorphene | 12306052 | |
| | | Beta-Selinene | 442393 | |

| | | | | |
|---|---|---|---|---|
| | | Epi-bicyclosesquiphellandrene | 91747125 | |
| | | Cadina-1,4-diene | 6427091 | |
| | | Bicyclogermacrene | 13894537 | |
| | | Endo-1-bourbonanol | 12301996 | |
| | | β-Maaliene | 101596917 | |
| | | Nerolidol | 5284507 | |
| | | Spathulenol | 92231 | |
| | | Caryophyllene oxide | 1742210 | |
| | | Guaiol | 227829 | |
| | | Beta-Himachalene | 11586487 | |
| | | τ-Muurolol | 51394521 | |
| | | Bulnesol | 90785 | |
| | | Farnesol | 445070 | |
| | | Farnesyl acetate | 94403 | |
| | | Benzyl benzoate | 2345 | |
| | | Benzyl salicylate | 8363 | |
| 44. | Areca catechu (35) | Arecoline | 2230 | 102 |
| | | Arecaidine | 10355 | |
| | | Arecolidine | 5319882 | |
| | | methyl nicotinate | 7151 | |
| | | ethyl nicotinate | 69188 | |
| | | ethyl N-methylpiperidine-3-carboxylate | 97981 | |
| | | isoguvacine | 3765 | |
| | | homoarecoline | 34167 | |
| | | isorhamnetin | 5281654 | |
| | | chrysoeriol | 5280666 | |
| | | liquiritigenin | 114829 | |
| | | jacareubin | 5281644 | |
| | | procyanidin A1 | 9872976 | |
| | | procyanidin B1 | 11250133 | |
| | | procyanidin B2 | 122738 | |
| | | arecatannin A1 | 13752000 | |
| | | arecatannin B1 | 14237657 | |
| | | arecatannin C1 | 9876038 | |
| | | arecatannin A2 | 16142155 | |
| | | arecatannin A3 | 16201011 | |
| | | arecatannin B2 | 71448962 | |
| | | ursonic acid | 9890209 | |
| | | Arborinol | 12305177 | |
| | | arborinol methyl ether | 101600057 | |
| | | fernenol | 12305178 | |
| | | arundoin | 12308619 | |
| | | cycloartenol | 92110 | |
| | | chrysophanol | 10208 | |
| | | physcion | 10639 | |
| | | p-hydroxybenzoic acid | 135 | |
| | | epoxyconiferyl alcohol | 57403796 | |
| | | isovanillic acid | 12575 | |
| | | de-O-methyllasiodiplodin | 14562693 | |
| | | cyclo-(Leu-Tyr) | 15550385 | |

| | | | | |
|---|---|---|---|---|
| 45. | Cannabis sativa (90) | Cannabisol | 102487751 | 103 |
| | | Cannabigerol | 5315659 | |
| | | $\alpha$-cadinyl-cannabigerolate | 24862530 | |
| | | carmagerol | 44586785 | |
| | | Sesquicannabigerol | 54669855 | |
| | | Cannabichromene | 30219 | |
| | | Cannabidiol | 644019 | |
| | | cannabidiolic acid | 160570 | |
| | | cannabidiol monomethyl ether | 164905 | |
| | | (-)-cannabidivarin | 11601669 | |
| | | cannabidivarinic acid | 59444387 | |
| | | Cannabinodiol | 11551346 | |
| | | cannabielsoic acid A | 59444405 | |
| | | cannabielsoin | 162113 | |
| | | cannabielsoic acid B | 59444401 | |
| | | Cannabicyclol | 30607 | |
| | | cannabicyclolic acid | 71437560 | |
| | | 8-hydroxycannabinolic acid A | 44139742 | |
| | | 8-hydroxycannabinol | 44241652 | |
| | | Dehydrocannabifuran | 59444381 | |
| | | Cannabifuran | 9966466 | |
| | | cannabicitran | 186149 | |
| | | cannabicoumaronone | 625303 | |
| | | cannabiripsol | 192007 | |
| | | Cannabimovone | 46217279 | |
| | | cannflavin C | 25141335 | |
| | | chrysoeriol | 5280666 | |
| | | 6-prenylapigenin | 10382485 | |
| | | Docosanoic acid methyl ester | 13584 | |
| | | acetyl stigmasterol | 129671096 | |
| | | $\alpha$-spinasterol | 5315190 | |
| | | quebrachitol | 151108 | |
| | | Isocannabispiradienone | 101802953 | |
| | | Cannabinol | 2543 | |
| | | Tetrahydrocannabivarin | 93147 | |
| | | tetrahydrocannabinolic acid | 98523 | |
| | | cannabinolic acid | 3081990 | |
| | | cannabigerolic acid | 6449999 | |
| | | cannabigerolic acid monomethyl ether | 24739091 | 104 |
| | | cannabigerovarinic acid | 59444383 | |
| | | cannabigerovarin | 59444407 | |
| | | cannabichromevarin | 6451726 | |
| | | cannabidivarin | 11601669 | |
| | | cannabidiorcol | 20586765 | |
| | | cannabicyclol | 30607 | |
| | | Cannabinolic acid A | 3081990 | |
| | | cannabinol methylether | 628150 | |
| | | cannabivarin | 622545 | |
| | | cannabichromanon | 25105340 | |
| | | trigonelline | 5570 | |

| | | | | |
|---|---|---|---|---|
| | | muscarine | 9308 | |
| | | neurine | 10042 | |
| | | Piperidine | 8082 | |
| | | Hordenine | 68313 | |
| | | n-propylamine | 7852 | |
| | | n-butylamine | 8007 | |
| | | diethylamine | 8021 | |
| | | pyrrolidine | 31268 | |
| | | Cannabisativine | 442846 | |
| | | Alanine | 602 | |
| | | galacturonic acid | 439215 | |
| | | erythritol | 222285 | |
| | | galactitol | 11850 | |
| | | ribitol | 6912 | |
| | | Malonic acid | 867 | |
| | | Phosphoric acid | 1004 | |
| | | Pyroglutamic acid | 7405 | |
| | | Quinic acid | 6508 | |
| | | Behenic acid | 633932 | |
| | | Sativic acid | 92800 | |
| | | hexyl caproate | 22873 | |
| | | campest-4-en-3-one | 11988279 | |
| | | camphenehydrate | 22155067 | |
| | | carvacrol | 10364 | |
| | | carvone | 7439 | |
| | | citronellol | 8842 | |
| | | dihydrocarveyl acetate | 30248 | |
| | | dihydrocarvone | 24473 | |
| | | pinocarveol | 102667 | |
| | | pinocarvone | 121719 | |
| | | allo-aromadendrene | 42608158 | |
| | | longifolene | 289151 | |
| | | nerolidol | 5284507 | |
| | | epi-beta-santalene | 11106484 | |
| | | vomifoliol | 5280462 | |
| | | dihydrovomifoliol | 129317062 | |
| | | cannabispiradienone | 90475437 | |
| | | beta-cannabispiranol | 194174 | |
| | | cannabispirenone | 10105874 | |
| | | cannabispirone | 162936 | |
| | | canniprene | 53439651 | |
| | | orientin | 5281675 | |
| 46. | Carthamus tinctorius (35) | Carthamin | 135565560 | 105 |
| | | Safflor yellow A | 71463725 | |
| | | Safflor yellow B | 131751452 | |
| | | Saffloflavonesides A | 101878345 | |
| | | Saffloflavonesides B | 101878346 | |
| | | Safflomin A (Hydroxysafflor yellow A) | 6443665 | |
| | | Tinctormine | 42607657 | |
| | | Safflomin C | 42607658 | |

| | | | | |
|---|---|---|---|---|
| | | Precarthamin | 101928647 | |
| | | Saffloquinoside A | 45276863 | |
| | | Saffloquinoside B | 101501319 | |
| | | Cartormin | 131751684 | |
| | | Anhydrosafflor yellow B | 102240413 | |
| | | Acacetin 7-O-alpha-L-rhamnopyranoside | 44257896 | |
| | | Luteolin 7-O-beta-D-glucopyranoside | 13093777 | |
| | | Acacetin | 5280442 | |
| | | Isorhamnetin | 5281654 | |
| | | Umbelliferone | 5281426 | |
| | | Daphnoretin | 5281406 | |
| | | N-feruloylserotonin | 5969616 | |
| | | N-(p-coumaroyl)serotonin | 5458879 | |
| | | Serotobenine | 11725426 | |
| | | N-feruloyltryptamine | 5458878 | |
| | | 1-Tridecene-3,5,7,9,11-pentayne | 441552 | |
| | | Sinapic acid | 637775 | |
| | | Roseoside | 9930064 | |
| | | Methylsyringin | 131752679 | |
| | | Coniferyl alcohol | 1549095 | |
| | | Sinapyl alcohol | 5280507 | |
| | | Secoisolariciresinol | 65373 | |
| | | Matairesinol | 119205 | |
| | | Arctigenin | 64981 | |
| | | Trachelogenin | 452855 | |
| 47. | Syzygium aromaticum (20) | Guaiacol | 460 | 106 |
| | | Phloroglucinol | 359 | 106 |
| | | Phenylacetic acid | 999 | 106 |
| | | Gentisic acid | 3469 | 106 |
| | | Naringenin Chalcone | 5280960 | 106 |
| | | Glycitein | 5317750 | 106 |
| | | Delphinidin | 68245 | 106 |
| | | Malvidin | 159287 | 106 |
| | | Allo Ocimene | 5368821 | 106 |
| | | Pinene-2-OL | 22013424 | 106 |
| | | Linalyl Acetate | 8294 | 106 |
| | | Elemicin | 10248 | 106 |
| | | 5-Hexene-2-one | 7989 | 107 |
| | | Guaiol | 227829 | 107 |
| | | Benzene-1-butylheptyl | 20661 | 107 |
| | | Nootkatin | 238797 | 107 |
| | | 9,17-Octadecadienal | 6431297 | 107 |
| | | Octadecanoic acid butyl ester | 31278 | 107 |
| 48. | Cornus officinalis (105) | Tellimagrandin II | 151590 | 108 |
| | | Cornusiin A | 16129730 | |
| | | Cornusiin B | 16131156 | |
| | | Cornusiin C | 16132407 | |
| | | Gemin D | 471119 | |
| | | Isoterchebin | 442685 | |
| | | Tellimagrandin I | 442690 | |

| | | | |
|---|---|---|---|
| | Cornusiin G | 102061533 | |
| | Cornusiin D | 101826455 | |
| | Zoomeric acid | 445638 | |
| | Nervonic acid | 5281120 | |
| | Lignoceric acid | 11197 | |
| | Phthalic anhydride | 6811 | |
| | Trans-9-Octadecenoic acid | 637517 | |
| | 3, 5-Bis (1, 1-dimethylethyl)phenol | 70825 | |
| | 4-Methyloctanoic acid | 62089 | |
| | 2-Dibenzofuransulfonic acid | 522803 | |
| | Morroniside | 11228693 | |
| | Cornuside | 11228694 | |
| | Loniceroside | 179500 | |
| | Kingiside | 12304884 | |
| | Sweroside | 161036 | |
| | Secoxyloganin | 162868 | |
| | 8-epikingiside | 12304886 | |
| | Secologanoside | 14136854 | |
| | Cornin (Verbenalin) | 73467 | |
| | Hastatoside | 92043450 | |
| | Loganin | 87691 | |
| | Loganic acid | 89640 | |
| | 7-O-methyl-morroniside | 127258930 | |
| | 7-O-ethyl-morroniside | 74399180 | |
| | 10-hydroxycornin | 101637374 | |
| | 10-hydroxyhastatoside | 100952769 | |
| | Secologanin | 161276 | |
| | Arjunglucoside II | 52951052 | |
| | Linalyl propionate | 61098 | |
| | 6, 10-Dimethyl-2-undecanone | 95495 | |
| | p-Menth-1-ene-7, 8-diol | 110662 | |
| | Cubebol | 11276107 | |
| | Tachioside | 11962143 | |
| | 5-hydroxymethyl-2-furfural | 237332 | |
| | Glycidol | 11164 | |
| | 3-Vinyl-1-cyclobutene | 556371 | |
| | 1,3-Cyclohexadiene | 11605 | |
| | Methacrolein | 6562 | |
| | 2-Vinylfuran | 73881 | |
| | 1-Ethoxypropan-2-yl acetate | 171378 | |
| | Pyridine | 1049 | |
| | Pyrrole | 8027 | 109 |
| | 3-Pyrrolidinol | 98210 | |
| | 3-Furaldehyde | 10351 | |
| | 3-Furanmethanol | 20449 | |
| | Propargylamine | 239041 | |
| | Furfural | 7362 | |
| | 2-Furanmethanol | 7361 | |
| | Cis-bicyclo[4.2.0]octane | 643590 | |
| | 4-Cyclopentene-1,3-dione | 70258 | |

| | | | |
|---|---|---|---|
| | Cyclopent-4-ene-1,3-dione | 70258 | |
| | 1-Nonene | 31285 | |
| | Pentanoic acid | 7991 | |
| | 2-Formylhistamine | 541600 | |
| | 2(5H)-Furanone | 10341 | |
| | 2-Cyclohexen-1-one | 13594 | |
| | 1-Methylpyrazol-3-amine | 137254 | |
| | 2H-Pyran-2-one | 68154 | |
| | 1H-Pyrrole-2-carboxaldehyde | 13854 | |
| | 4-Methyl-5H-furan-2-one | 145832 | |
| | p-Cresol | 2879 | |
| | 2-Pentyne | 12310 | |
| | Maltol | 8369 | |
| | Benzyl nitrile | 8794 | |
| | Dehydromevalonic lactone | 557445 | |
| | E-7-Tetradecenol | 5362726 | |
| | Creosol | 7144 | |
| | Catechol | 289 | |
| | 5-Hydroxymethylfurfural | 237332 | |
| | 2-Coumaranone | 68382 | |
| | 4,6-Dioxadodecane | 57645665 | |
| | 1-Tridecene | 17095 | |
| | Kessane | 11310616 | |
| | Oxaceprol | 65784 | |
| | 2-Methoxy-4-vinylphenol | 332 | |
| | 1,2,3-Benzenetriol | 1057 | 109 |
| | 1,2,4-Benzenetriol | 10787 | |
| | 3,4-Dimethyl-o-phenylenediamine | 521077 | |
| | 3,5-Dimethoxy-4-hydroxytoluene | 240925 | |
| | 4-Methoxycinnamaldehyde | 641294 | |
| | D-Allose | 439507 | |
| | 1-Pentadecene | 25913 | |
| | Pentadecane | 12391 | |
| | Cyclotetradecane | 67524 | |
| | Butyrovanillone | 14035098 | |
| | Palmitoleic acid | 445638 | |
| | Tridecanoic acid | 12530 | |
| | 1-Heptadecene | 23217 | |
| | 9-Hexadecenoic acid | 5282745 | |
| | 5-Amino-2-thiocyanoacetophenone | 284762 | |
| | Cyanoacetic acid, dodecyl ester | 12606407 | |
| | 1,5-Dodecadiene | 5363382 | |
| | cis-1-Chloro-9-octadecene | 5367784 | |
| | 1-Nonadecene | 29075 | |
| | 1-Octadecene | 8217 | |
| | Methyl 13-methyl-eicosanoate | 85964992 | |
| | Tricosane | 12534 | |
| | Oxacycloheptadecan-2-one | 7984 | |
| | 1-Docosene | 74138 | |

| | | | | |
|---|---|---|---|---|
| 49. | Eucommia ulmoides (55) | (−)-olivil | 5273570 | 110 |
| | | Lariciresinol | 332427 | |
| | | Balanophonin | 23252258 | |
| | | vladinol D | 70698172 | |
| | | (+)-cyclo-olivil | 5316262 | |
| | | (+)-medioresinol | 181681 | |
| | | (+)-pinoresinol | 73399 | |
| | | (+)-syringaresinol | 443023 | |
| | | (+)-epipinoresinol | 637584 | |
| | | Arctiin | 100528 | |
| | | citrusin B | 131752580 | |
| | | threo-dihydroxydehydrodiconiferyl alcohol | 5317204 | |
| | | hedyotol C | 21636185 | |
| | | genipin | 442424 | |
| | | aucubin | 91458 | |
| | | geniposide | 107848 | |
| | | geniposidic acid | 443354 | |
| | | asperulosidic acid | 11968867 | |
| | | asperulosidic acid ethyl ester | 21580986 | |
| | | Ajugoside | 9865184 | |
| | | Reptoside | 44584096 | |
| | | eucommiol | 154373 | |
| | | eucommioside II | 6325167 | |
| | | 1-deoxyeucommiol | 5316559 | |
| | | Epieucommiol | 101714787 | |
| | | ulmoidoside A | 14540382 | |
| | | ulmoidoside B | 14540384 | |
| | | asperuloside | 84298 | 110 |
| | | ulmoside | 11968295 | |
| | | eucomoside A | 102382672 | |
| | | eucomoside B | 23624546 | |
| | | eucomoside C | 23625086 | |
| | | daphylloside | 21602024 | |
| | | scandoside methyl ester | 442433 | |
| | | loganin | 87691 | |
| | | 7-epi-loganin | 10548420 | |
| | | artselaenin C | 100930978 | |
| | | harpagide | 10044294 | |
| | | catalpol | 91520 | |
| | | avicularin | 5490064 | |
| | | oroxylin A | 5320315 | |
| | | licochalcone A | 5318998 | |
| | | thunberginol C | 10333412 | |
| | | C-veratroylglycol | 15765124 | |
| | | alternariol | 5359485 | |
| | | salicifoliol | 10955962 | |
| | | eucophenoside | 102044905 | |
| | | Uvaol | 92802 | |
| | | eleganoside A | 101085911 | |
| | | borreriagenin | 44583980 | |

| | | | | |
|---|---|---|---|---|
| | | 3-O-acetylerythrodiol | 118796402 | 111 |
| | | aplyolide D | 10708956 | |
| | | Umbelliferone | 5281426 | |
| | | Eriodictyol | 440735 | |
| | | Homoeriodictyol | 73635 | |
| | | Aromadendrol | 122850 | |
| | | cyclo(Pro-Pro) | 529063 | |
| 50. | Foeniculum vulgare (55) | Anisketone | 31231 | 112 |
| | | Apiol | 10659 | |
| | | 4-Carene | 530422 | |
| | | 3-Carene | 26049 | |
| | | Cathine | 441457 | |
| | | 2-Propyn-1-ol | 7859 | |
| | | 2,6-Dimethyl-2,4,6-octatriene | 5368821 | |
| | | Sabinene hydrate | 62367 | |
| | | Fenchyl acetate | 107217 | |
| | | Dicyclopropyl carbinol | 84336 | |
| | | Estragole | 8815 | |
| | | *trans*-p-2,8-menthadien-1-ol | 155626 | |
| | | 1,4-Dimethoxybenzene | 9016 | |
| | | *trans*-Anethole | 637563 | |
| | | Allantoic acid | 203 | |
| | | 1-Undecanol | 8184 | |
| | | Benzothiazole | 7222 | |
| | | 1,6-Hexanediol | 12374 | |
| | | Mephenesin | 4059 | |
| | | 4-Fluorohistamine | 541569 | |
| | | 1-(3-Methoxyphenyl)-1-propanone | 584765 | |
| | | 1,5 dicaffeoylquinic acid | 122685 | 113 |
| | | Hesperidin | 10621 | |
| | | 3- caffeoylquinic acid | 102111217 | |
| | | 4- caffeoylquinic acid | 9798666 | |
| | | imperatorin | 10212 | |
| | | psoralen | 6199 | |
| | | bergapten | 2355 | |
| | | xanthotoxin | 4114 | |
| | | isopimpinellin | 68079 | |
| | | Cuminal | 326 | |
| | | cis-Anethole | 1549040 | |
| | | p-Anisaldehyde | 31244 | |
| | | 2-methyl-3-oxoestran- 17-yl acetate | 22212600 | |
| | | Vetivenene | 529892 | |
| | | Anthracene | 8418 | |
| | | 2-Methoxy-4-ethylphenol | 62465 | |
| | | 1,2-Dimethoxy-4-methylbenzene | 68126 | |
| | | Cedran-9-one | 21723990 | |
| | | 2,2-dimethyl-3-phenylpropanoate | 6947002 | |
| | | 3,3,6-Trimethyl-1-indanone | 594538 | |
| | | o-Benzenedicarboxylic acid | 1017 | |
| | | 3,4-Dimethyl-1,5-cyclooctadiene | 5365753 | |

| | | |
|---|---|---|
| | capric acid | 2969 |
| | undecanoic acid | 8180 |
| | pentadecadienoic acid | 19047235 |
| | erucic acid | 5281116 |
| | n-Hexacosane | 12407 |
| | n-Heptacosane | 11636 |
| | n-Octacosane | 12408 |
| | n-Nonacosane | 12409 |
| | n-Triacontane | 12535 |
| | n-Dotriacontane | 11008 |
| | n-Tetratriacontane | 26519 |
| | n-Hexatriacontane | 12412 |
| | n-Octatriacontane | 23599 |
| | n-Tetracontane | 20149 |

References:


1. Anti-inflammatory and PPAR transactivational effects of secondary metabolites from the roots of Asarum sieboldii
2. Fumigant Toxicity of Phenylpropanoids Identified in *Asarum sieboldii* Aerial Parts to *Lycoriella ingenua* (Diptera: Sciaridae) and *Coboldia fuscipes* (Diptera: Scatopsidae)
3. The Genus Asarum L.: A Phytochemical and Ethnopharmacological Review
4. *Scutellaria baicalensis* Georgi. (Lamiaceae): a review of its traditional uses, botany, phytochemistry, pharmacology and toxicology
5. Cinnamomum cassia Presl: A Review of Its Traditional Uses, Phytochemistry, Pharmacology and Toxicology
6. Antiinflammatory effects of essential oil from the leaves of Cinnamomum cassia and cinnamaldehyde on lipopolysaccharide-stimulated J774A. 1 cells
7. Analysis and evaluation of essential oil components of cinnamon barks using GC–MS and FTIR spectroscopy
8. Anti-diabetic nephropathy compounds from Cinnamomum cassia
9. Five new diterpenoids from the barks of Cinnamomum cassia (L.) J. Presl
10. Diterpenoids with Immunosuppressive Activities from *Cinnamomum cassia*
11. Chemical composition and tyrosinase inhibitory activity of Cinnamomum cassia essential oil
12. Differentiating parts of *Cinnamomum cassia* using LC-qTOF-MS in conjunction with principal component analysis
13. Xanthine oxidase inhibitory activity of constituents of Cinnamomum cassia twigs
14. Tissue-specific chemical profiling and quantitative analysis of bioactive components of Cinnamomum cassia by combining laser-microdissection with UPLC-Q …
15. Two new geranylphenylacetate glycosides from the barks of *Cinnamomum cassia*
16. Nitric oxide inhibitory constituents from the barks of Cinnamomum cassia
17. Identification of compounds from the water soluble extract of Cinnamomum cassia barks and their inhibitory effects against high-glucose-induced mesangial cells
18. A New Lactone from the Twigs of *Cinnamomum cassia*
19. Bioactive Constituents of *Glycyrrhiza uralensis* (Licorice): Discovery of the Effective Components of a Traditional Herbal Medicine
20. Studies on chemical constituents on roots of Glycyrrhiza uralensis
21. Recent Advances in *Astragalus membranaceus* Anti-Diabetic Research: Pharmacological Effects of Its Phytochemical Constituents
22. Phellodendri Cortex: A Phytochemical, Pharmacological, and Pharmacokinetic Review
23. Enzyme-assisted Extraction of Bioactive Phytochemicals from Japanese Peppermint (Mentha arvensis L. cv. 'Hokuto')
24. Chemical Constituents from the Aerial Parts of Bupleurum falcatum L. and Biological Evidences
25. Chemical constituents analysis on the seeds of Bupleurum Falcatum L.
26. A Pharmacological Review of Bioactive Constituents of Paeonia lactiflora Pallas and Paeonia veitchii Lynch
27. Platelet anti-aggregatory and blood anti-coagulant effects of compounds isolated from Paeonia lactiflora and Paeonia suffruticosa
28. Pharmacological effects of medicinal components of *Atractylodes lancea* (Thunb.) DC.
29. Atractylodis Rhizoma: A review of its traditional uses, phytochemistry, pharmacology, toxicology and quality control
30. Chemical Constituents from Cimicifuga dahurica and Their Anti-Proliferative Effects on MCF-7 Breast Cancer Cells
31. Traditional uses, phytochemistry, pharmacology and toxicology of the genus Cimicifuga: A review

32. Assessment of phenolics contents and antioxidant properties in *Cimicifuga dahurica* (Turcz.) Maxim during drying process
33. Anti-inflammatory activity of compounds from the rhizome of Cnidium officinale
34. Chemical constituents from lipophilic parts in roots of Angelica dahurica var. formosana cv. Chuanbaizhi
35. Coumarins from the roots of Angelica dahurica cause anti-allergic inflammation
36. Composition and stereochemistry of ephedrine alkaloids accumulation in Ephedra sinica Stapf
37. A-Type Proanthocyanidins from the Stems of Ephedra sinica (Ephedraceae) and Their Antimicrobial Activities
38. Phytochemistry and pharmacology of genus Ephedra
39. Chemical Constituents from Seeds of Panax ginseng: Structure of New Dammarane-Type Triterpene Ketone, Panaxadione, and HPLC Comparisons of Seeds and Flesh
40. The Chemical Constituents of Ginseng Plants
41. Some phytochemical, pharmacological and toxicological properties of ginger (Zingiber officinale Roscoe): A review of recent research
42. PHYCHEMISTRY, PHYTOCHEMICAL, PHARMACOLOGICAL AND MOLECULAR STUDY OF ZINGIBER OFFICINALE ROSCOE: A REVIEW
43. Plants of the Genus Zingiber as a Source of Bioactive Phytochemicals: From Tradition to Pharmacy
44. Phytochemical and chemotaxonomic study of Poria cocos (Schw.) Wolf
45. Chemical Constituents and Pharmacological Properties of Poria cocos
46. Phytochemical variation among the traditional Chinese medicine Mu Dan Pi from Paeonia suffruticosa (tree peony)
47. Studies on Chemical Constituents from Seeds of Paeonia suffruticosa Andr.
48. Chemical constituents, antibacterial activity and mechanism of Paeonia suffruticosa Andr. buds extract against Staphylococcus aureus and Escherichia coli O157:H7
49. Origins, Phytochemistry, Pharmacology, Analytical Methods and Safety of Cortex Moutan (*Paeonia suffruticosa* Andrew): A Systematic Review
50. Chemical constituents of lateral roots of Aconitum carmichaelii Debx
51. A review on phytochemistry and pharmacological activities of the processed lateral root of Aconitum carmichaelii Debeaux
52. Comparative Analysis of the Major Chemical Constituents in *Salvia miltiorrhiza* Roots, Stems, Leaves and Flowers during Different Growth Periods by UPLC-TQ-MS/MS and HPLC-ELSD Methods
53. Salvia miltiorrhiza: Chemical and pharmacological review of a medicinal plant
54. Bioactive phytochemicals from shoots and roots of Salvia species
55. Major Phytochemical Composition of 3 Native Korean Citrus Varieties and Bioactive Activity on V79-4 Cells Induced by Oxidative Stress
56. Phenolic Compounds and Antioxidant Activity of Extracts from Ultrasonic Treatment of Satsuma Mandarin (*Citrus unshiu* Marc.) Peels
57. Flavonoids and a Limonoid from the Fruits of Citrus unshiu and Their Biological Activity
58. Semi-Continuous Subcritical Water Extraction of Flavonoids from Citrus unshiu Peel: Their Antioxidant and Enzyme Inhibitory Activities
59. Chemical Composition and Anti-inflammation Activity of Essential Oils from *Citrus unshiu* Flower
60. Phytochemistry, Pharmacological Activities and Intellectual Property Landscape of Gardenia jasminoides Ellis: a Review
61. Chemical Study on Aerial Parts of Gentiana scabra.
62. The anti-inflammatory secoiridoid glycosides from Gentianae Scabrae Radix: the root and rhizome of Gentiana scabra
63. Triterpenoids isolated from the rhizomes and roots of Gentiana scabra and their inhibition of indoleamine 2,3-dioxygenase
64. A review of the phytochemistry and pharmacological activities of Magnoliae officinalis cortex
65. Ophiopogon japonicus—A phytochemical, ethnomedicinal and pharmacological review
66. Rapid Screening and Identification of Chemical Constituents From Ophiopogon japonicus by High-Performance Liquid Chromatography Coupled to Electrospray Ionization and Quadrupole Time-of-Flight Mass Spectrometry
67. Ethnomedicinal, Phytochemical and Pharmacological Investigations of Perilla frutescens (L.) Britt.
68. Isolation of Phenolics, Nucleosides, Saccharides and an Alkaloid from the root of Aralia cordata
69. Inhibitory Constituents against Cyclooxygenases from Aralia cordata Thunb
70. Quantitative Determination of Diterpenoids from the Roots of Aralia cordata
71. Comprehensive profiling and characterization of chemical constituents of rhizome of Anemarrhena asphodeloides Bge.
72. Anti-Inflammatory Activities of Compounds Isolated from the Rhizome of Anemarrhena asphodeloides
73. The genus Anemarrhena Bunge: A review on ethnopharmacology, phytochemistry and pharmacology
74. A review of the pharmacological effects of *Arctium lappa* (burdock)
75. Inhibitory Compounds of $\alpha$-Glucosidase Activity from *Arctium lappa* L.
76. Analysis of Seasonal Variations of the Volatile Constituents in Artemisia princeps (Japanese Mugwort) Leaves by Metabolomic Approach
77. Chemical constituents from the tubers of Pinellia ternata (Araceae) and their chemotaxonomic interest
78. The ethnobotanical, phytochemical and pharmacological profile of the genus Pinellia

79. Platycodon grandiflorus – An Ethnopharmacological, phytochemical and pharmacological review
80. The Pharmacological Effects and Health Benefits of Platycodon grandiflorus—A Medicine Food Homology Species
81. A new megastigmane from fresh roots of *Rehmannia glutinosa*
82. Phytochemical Studies on Rehmanniae Radix
83. Phytochemical studies of Rehmannia glutinosa rhizomes
84. Chemical constituents from Rehmannia glutinosa
85. Study on Chemical Constituents of Immunosuppressive Parts from the Roots of Rehmannia glutinosa
86. Saposhnikovia divaricata—An Ethnopharmacological, Phytochemical and Pharmacological Review
87. Studies on chemical constituents on roots of Glycyrrhiza uralensis
88. ZIZIPHUS JUJUBA: A PHYTOPHARMACOLOGICAL REVIEW
89. Chemical characterization of the main bioactive constituents from fruits of *Ziziphus jujuba*
90. Ziziphus jujuba Mill., a plant used as medicinal food: a review of its phytochemistry, pharmacology, quality control and future research
91. A Promising View of Kudzu Plant, Pueraria montana var. lobata (Willd.) Sanjappa & Pradeep: Flavonoid Phytochemical Compounds, Taxonomic Data, Traditional Uses and Potential Biological Activities for Future Cosmetic Application
92. Chemical constituents from roots of Pueraria lobata
93. Phytochemistry and health promoting effects of Job's tears (Coix lacryma jobi) - A critical review
94. Phytochemical Screening and Antibacterial Activity Coix lacryma-jobi Oil
95. Chemistry, pharmacology and processing method of rhubarb (Rheum species): a review
96. Medicinal uses, phytochemistry and pharmacology of the genus Uncaria
97. Phytochemistry, pharmacology, quality control and future research of Forsythia suspensa (Thunb.) Vahl: A review
98. Alisma orientale: Ethnopharmacology, Phytochemistry and Pharmacology of an Important Traditional Chinese Medicine
99. Phytochemical Constituents of Amomum xanthioides
100. Terpene Glycosides and Cytotoxic Constituents from the Seeds of *Amomum xanthioides*
101. Chemical Composition of Essential Oil of Amomum xanthioides Wall. ex Baker from Northern Vietnam
102. Areca catechu L. (Arecaceae): A review of its traditional uses, botany, phytochemistry, pharmacology and toxicology
103. Phytochemistry of Cannabis sativa L.
104. CONSTITUESTS OF CAS-TABIS SATIVA L. XT'II. Ai REVIEIT OF THE XXTURXL CONSTITUENTS
105. Phytochemistry and Pharmacology of Carthamus tinctorius L.
106. Phytochemical Screening and Antimicrobial Evaluation of Syzygium aromaticum Extract and Essential oil
107. Chemical Constituents of Clove (Syzygium aromaticum, Fam. Myrtaceae) and their Antioxidant Activity
108. Ethnopharmacology, phytochemistry, and pharmacology of Cornus officinalis Sieb. et Zucc
109. Molecules and functions of Cornus officinalis bark volatiles
110. Ethnobotany, Phytochemistry and Pharmacological Properties of Eucommia ulmoides: A Review
111. Phytochemical constituents, chemotaxonomic significance and anti-arthritic effect of Eucommia ulmoides Oliver staminate flowers
112. Foeniculum vulgare Mill: A Review of Its Botany, Phytochemistry, Pharmacology, Contemporary Application, and Toxicology
113. The chemical constituents and pharmacological effects of Foeniculum vulgare - A review
